# Gold(I)-Mediated Rapid Cyclization of Propargylated Peptides via Imine Formation

**DOI:** 10.1021/jacs.1c12906

**Published:** 2022-03-08

**Authors:** Rajeshwer Vanjari, Deepanjan Panda, Shaswati Mandal, Ganga B. Vamisetti, Ashraf Brik

**Affiliations:** Schulich Faculty of Chemistry, Technion − Israel Institute of Technology, Haifa 3200008, Israel

## Abstract

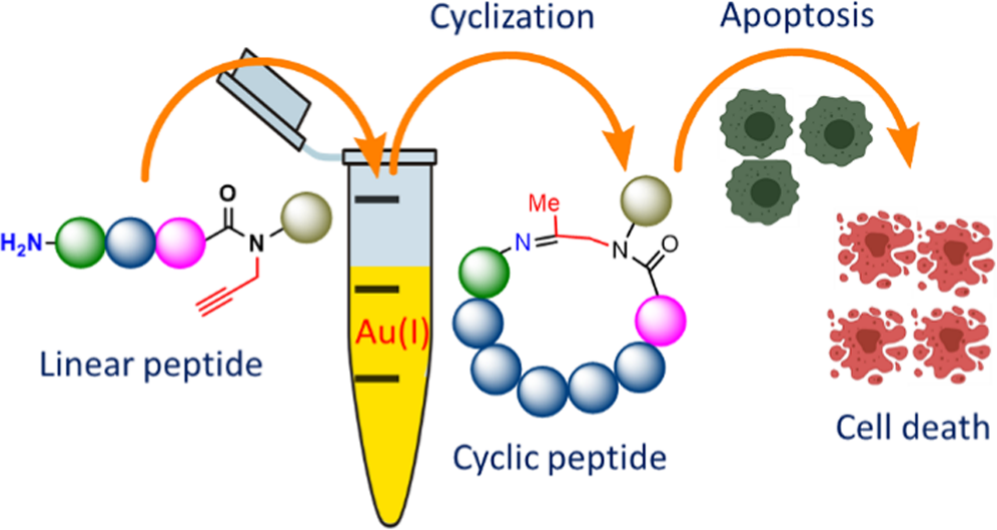

In fundamental research and drug discovery, there is still a need for effective and straightforward chemical approaches for generating cyclic peptides. The divergent synthesis of cyclic peptides remains a challenge, in particular when cyclization is carried out in the presence of unprotected side chains and a nonpeptidic component within the cycle is needed. Herein, we describe a novel and efficient strategy based on Au(I)-mediated cyclization of unprotected peptides through rapid (30–60 min) amine addition on a propargyl group to generate an imine linkage. Mechanistic insights reveal that the reaction proceeds via regioselective Markovnikov’s addition of the amine on the Au(I)-activated propargyl. This strategy was successfully applied to prepare efficiently (56–94%) over 35 diverse cyclic peptides having different sequences and lengths. We have also achieved stereoselective reduction of cyclic imines employing chiral ligands. The practicality of our method was extended for the synthesis of cyclic peptides that bind Lys48-linked di-ubiquitin chains with high affinity, leading to apoptosis of cancer cells.

## Introduction

Owing to their high efficacy in targeting protein–protein interactions, cyclic peptides have sparked a lot of interest. The conformational restraint enforced by cyclization of the peptides often increases their binding to the target and render them less susceptible to enzymatic degradation, thus increasing their biostability and bioavailability.^1^ Therefore, the increased molecular weight of these compounds is often balanced by their high specificity and binding toward the targets. As a result, cyclic peptides fill the “space” that could not be filled by small molecules in the so-called “undruggable targets”, involving protein–protein interactions. Cyclic peptides, as of today, constitute a powerful mimic to biologics, showing great potential in drug discovery and basic research.^2,3^

While biological approaches allow for the rapid discovery of cyclic peptides from large libraries,^4−8^ their laboratory synthesis can be challenging. The reduced entropy upon cyclization is a major impediment for the synthesis of cyclic peptides, in addition to other issues such as C-terminal epimerization and oligomerization.^9,10^ Moreover, the incorporation of nonpeptidic scaffolds into macrocycles is highly desirable for tuning the cyclic peptide activity and physical properties.^11^ As a result, new synthetic approaches that can overcome these challenges and incorporate desirable features into cyclic peptides are still required.^12^

**Scheme 1:**
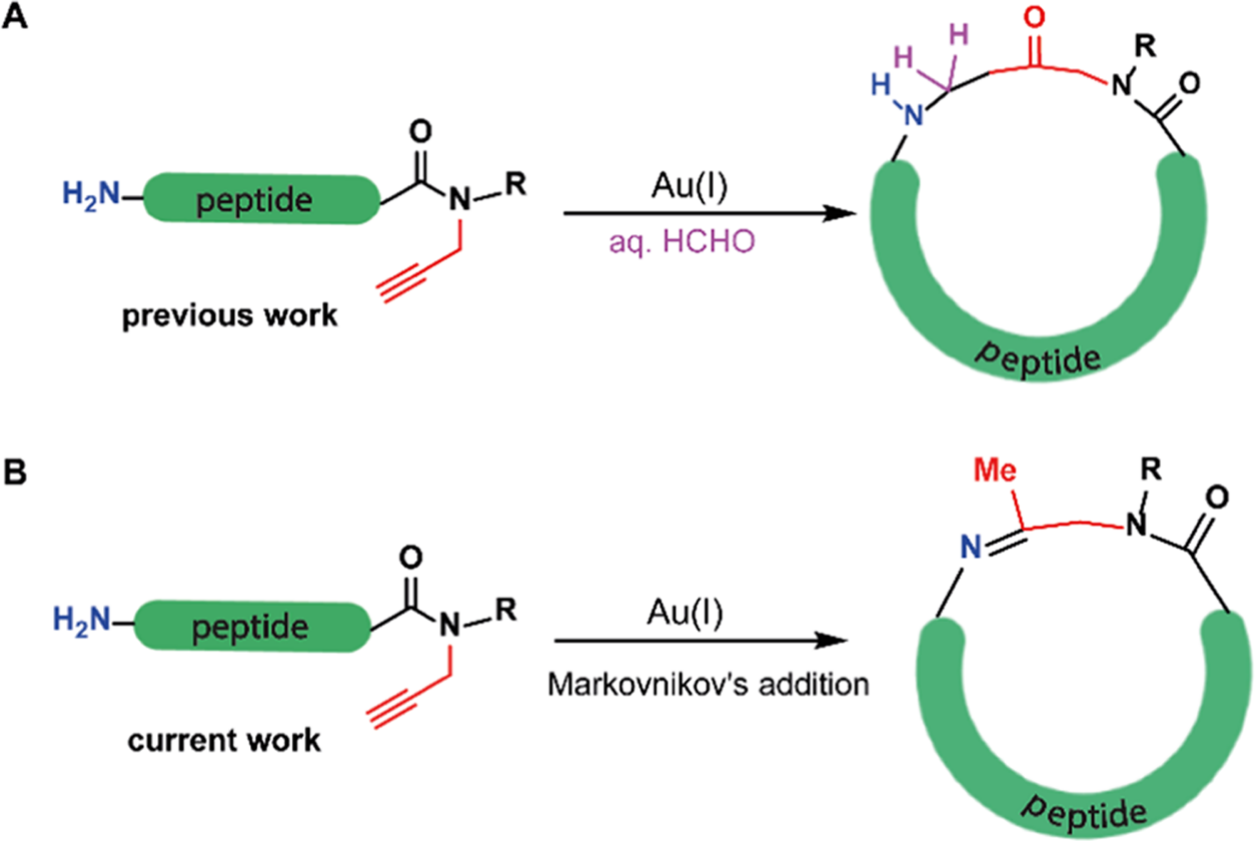
(A) Peptide Cyclization via Gold(I)-Mediated Keto Linkage Formation Between the *N*-Terminus and Propargylated Amide and (B) Peptide Cyclization via Gold(I)-Mediated Imine Formation Between the *N*-Terminus and Propargylated Amide

Despite the use of gold complexes in organic transformations,^13^ their applications in peptide and protein chemistry have been limited, in contrast to other transition metals.^14−22^ Expanding the utility of gold complexes in these areas can be fruitful,^23−25^ in particular when one takes advantage of their unique reactivities, stability, and water compatibility. We have recently started to apply gold chemistry in peptide/protein syntheses and reported Au(I)Cl-promoted depropargylation/amide bond cleavage of *N*-propargylated peptide.^26^ In the context of peptide cyclization, we have also reported the use of (JohnPhos)Au(I)(ACN)SbF_6_ to facilitate cyclization of peptides bearing propargyl functionality and a free amine in the presence of formaldehyde as a reactant^27^ (Scheme 1A).

We present here the development of a novel direct imine-forming cyclization method for unprotected peptides using gold-mediated cyclization of backbone-propargylated peptides (Scheme 1B). Structurally diverse cyclic peptides of different sizes and sequences have been prepared from their linear counterparts using the current method. We also examined the diastereoselective reduction of cyclic imines in the presence of chiral ligands. We then used our method to create cyclic peptides to modulate Lys48-linked di-Ub chains *in vitro*, demonstrating the added value of the new cyclization approach in inducing apoptosis of cancer cells.

## Results and Discussion

Despite the efficiency of our previous cyclization reaction and its utility in the discovery of cyclic peptides with enhanced binding affinity against ubiquitin chains, we wondered whether in the absence of formaldehyde, as an reactant, a free amine in the peptide could act as a nucleophile on the gold-activated alkyne to form different connectivities.^28^ Our rationale also relies on the previous observation of water addition on the propragyl-activated gold. Therefore, a nucleophilic amine could act similar to water and add to the alkyne. As a result, this could lead to cyclization via imine functionality and generate a new linkage^29^ with different properties such as potency of binding, conformational space, and permeability of the cyclic peptides.

To test our assumption and whether in the absence of formaldehyde, a free amine group in a peptide would act as a nucleophile on the gold-activated alkyne, we planned a reaction of *N*-propargylated peptide in the presence of the JohnPhosAu(MeCN)SbF_6_ complex. We postulated that the attack of the *N*-terminus amine at the β-position (Markovnikov’s addition) of the Au-activated propargyl, followed by the protodeauration event, would lead to selective cyclization.

**Figure 1:**
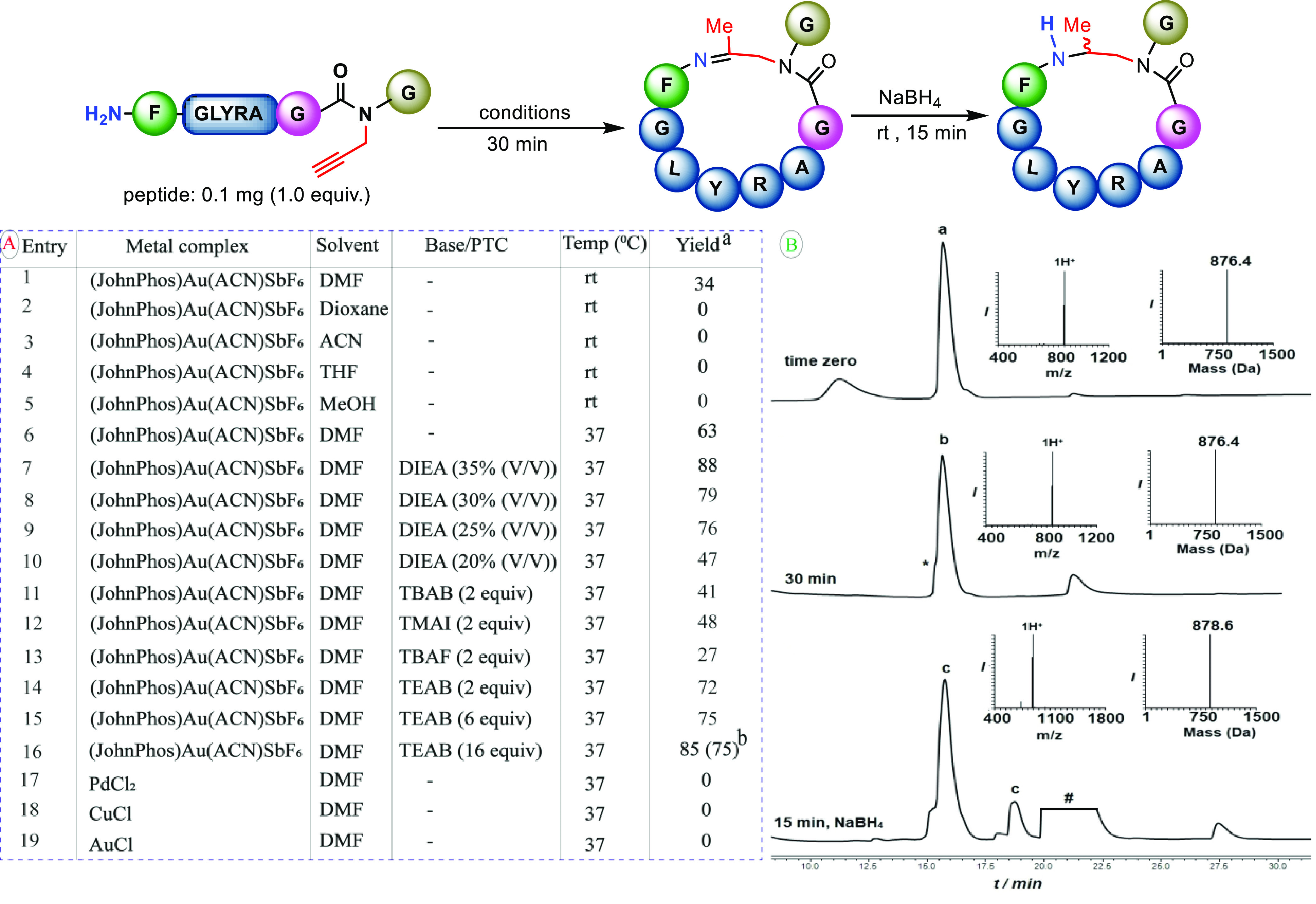
(A) Reaction optimization: metal complex (2.0 equiv), solvent (8.0 mM) at rt or 37 °C for 30 min, and followed by NaBH_4_ (10.0 equiv) reduction for 15 min at rt^a,b^. (B) FGLYRAG(prop)G peptide reaction with JohnPhosAu(ACN)SbF_6_ after 30 min of incubation at 37 °C. Peak a corresponds to FGLYRAG(prop)G with the observed mass of 876.4 ± 0.0 Da (calcd 876.9 Da). Peak b corresponds to the cyclized product with the observed mass of 876.4 ± 0.0 Da (calcd 876.9 Da). Peak c corresponds to the reduced cyclized products (diastereomers) after 15 min of incubation at rt with NaBH_4_ with the observed mass of 878.6 ± 0.0 Da (calcd 878.9 Da). * refers to partial hydrolysis of the imine during the HPLC analysis. # refers to the metal complex. ^a^Yields based on LC-MS analysis of the crude reaction mixture. ^b^Isolated yield in the parenthesis.

Our investigation was initiated by performing a reaction on a model peptide (FGLYRAG(Prop)G) in DMF at room temperature. Pleasantly, the desired cyclization was observed with 34% conversion yield (Figure 1A, entry 1). Subsequently, we investigated the cyclization reaction by experimenting various conditions, such as employing different metal complexes, additives, solvents, and temperatures. Of the tested conditions, the (JohnPhos)Au(ACN)SbF_6_ revealed encouraging results at 37 °C in DMF (Figure 1A, entries 2–6). The reaction efficiency was further improved by adding *N*,*N*-diisopropylethylamine (DIEA), (35% v/v), which resulted in product formation with 88% conversion yield (Figure 1A, entry 7). Next, we attempted to decrease the amount of DIEA, but the reaction yield significantly dropped (Figure 1A, entries 8–10). This is probably due to the role of DIEA in stabilizing the counter ion by forming a quaternary ammonium complex with SbF_6_ (DIEA + HSbF_6_); thereby, the solubility of the catalyst and stability of SbF_6_ would increase. Therefore, decreasing the amount of DIEA in the reaction mixture could decrease the stability of the counter ion, leading to the observed significant drop in the reaction efficiency.

To further support this notion, we thought to employ known quaternary ammonium salts, usually used as phase-transfer catalysts (PTCs). For this, several salts were checked in the presence of JohnPhosAu(MeCN)SbF_6_ in DMF (Figure 1A, entries 11–15). After testing several salts, we found that addition of tetraethylammonium bromide (TEAB, 16 equiv, compared to 35% v/v DIEA) assisted the reaction and gave the desired product in 85% conversion yield and 75% isolated yield, within 30 min (Figure 1A, entry 16 and Figure 1B).

Examining other metal complexes for this reaction did not promote product formation (Figure 1A, entries 17–19). Another interesting observation we made during optimization is that the imine linkage was stable during HPLC analysis. Only in some cases, the cyclic product was partially hydrolyzed to the linear peptide in the acidic solvent system. To further stabilize the cyclized peptides, one-pot reduction was performed by adding NaBH_4_ to the reaction mixture. This reaction afforded two different diastereomers of the cyclic peptide, which often separated in the HPLC analysis (Figure 1B).

### Mechanistic Insights

**Figure 2:**
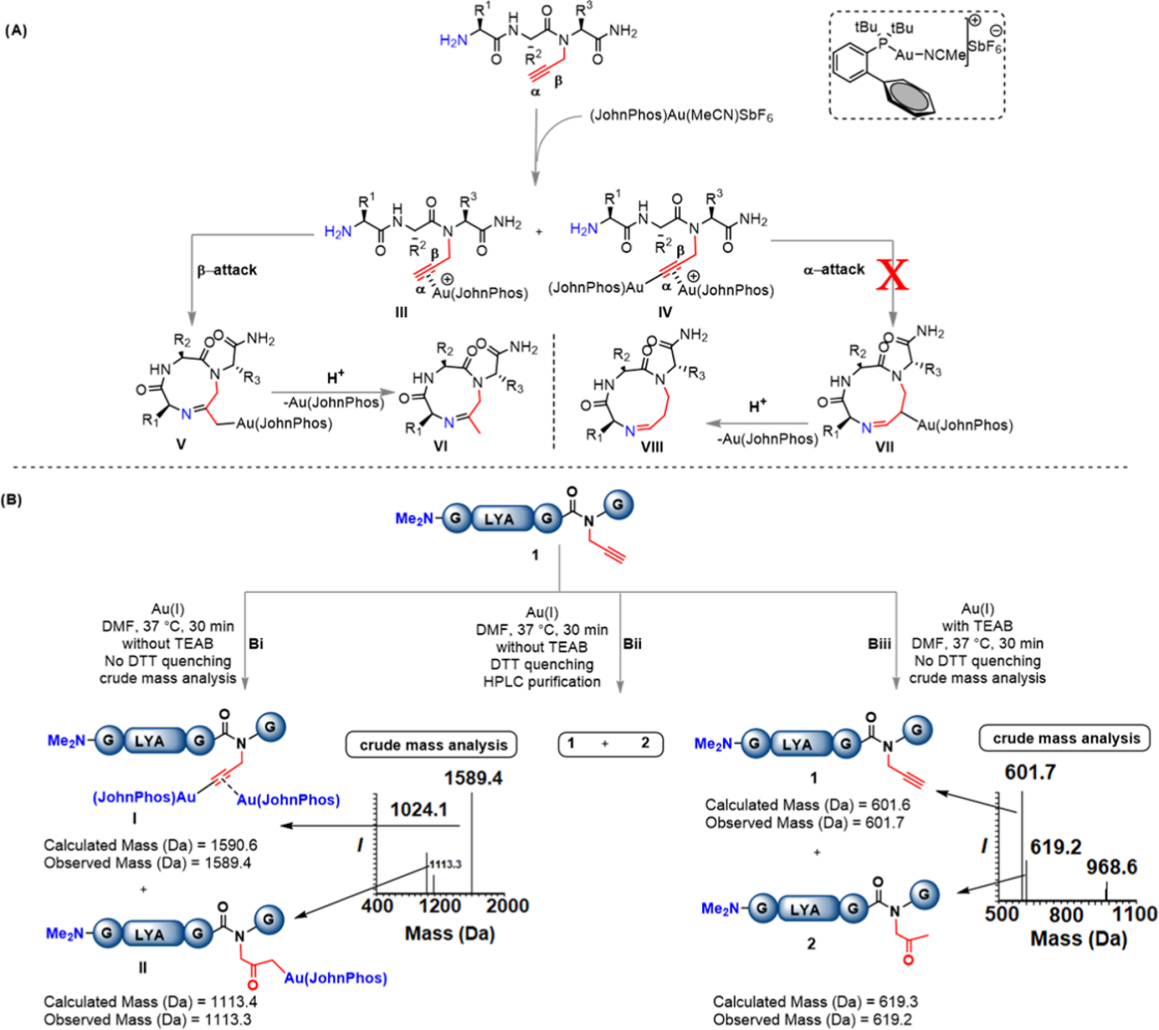
(A) Proposed mechanism for the gold-mediated cyclization. (B) Control experiments of the dimethylated *N*-terminal model peptide **1**. (B(I)) Peptide **1** was incubated at 37 °C for 30 min with (JohnPhos)Au(ACN)SbF_6_ (2.0 equiv), without TEAB and analyzed the crude with ESI-Mass without DTT quenching. (B(II)) Peptide **1** was incubated at 37 °C for 30 min with (JohnPhos)Au(ACN)SbF_6_ (2.0 equiv), without TEAB and after DTT quenching; analyzed with HPLC and mass spectrometry. (B(III)) Peptide **1** was incubated at 37 °C for 30 min with (JohnPhos)Au(ACN)SbF_6_ (2.0 equiv) and TEAB followed by crude analysis using ESI-mass spectrometry without DTT quenching.

Based on our experimental observations and the previous literature on gold chemistry,^30−35^ we propose a plausible mechanism for the cyclization reaction (Figure 2A). The reaction starts by coordinating the gold to the alkyne functionality and forming the π-alkyne–gold(I) complex **III**. The terminal alkyne would then be deprotonated by the counter ion SbF6^–^, followed by the coordination of a second gold complex, yielding a σ-alkynyl gold(I) complex **IV**. It is unclear which complex, **III** or **IV**, is the active species that initiates the Markovnikov addition of the amine (β-attack) to form intermediate **V**, which after protodeauration yields the cyclized product **VI**. Anti-Markovnikov’s addition of the amine (α-attack) to form the intermediate **VII**, via a similar gold intermediate, followed by cyclization to form product **VIII** was not observed (Supporting Information, S47, confirmed by NMR).

To support our proposed mechanism, we attempted to capture the reaction intermediate(s) by protecting the *N*-terminal amine, therefore inhibiting the cyclization (Figure 2A). Hence, *N*-terminally protected model peptide **1**, (*N*(Me)_2_-GLYAG(Prop)G), was synthesized. Peptide **1** was subjected to different reaction conditions, and the crude mixture was further analyzed by HPLC and mass spectrometry, separately (Figure 2B). In the first experiment, the reaction was performed in the presence of gold without TEAB in DMF for 30 min. The crude mixture was filtered; an aliquot of the reaction mixture was diluted with 50% MeOH in H_2_O and analyzed with mass spectrometry without quenching with dithiothreitol (DTT). Here, we observed the dinuclear gold complex with the peptide (**I**) and the gold-acylated peptide intermediate, **II** (Figure 2B(I)). In the second experiment, the reaction was performed in the presence of gold without TEAB in DMF for 30 min, followed by quenching with DTT. HPLC analysis and purification revealed the starting material (**1**) and the water addition product (**2**), as was shown by mass analysis (Figure 2B(II)). In the third experiment, the reaction was carried for 30 min in the presence of the gold complex and TEAB. The crude mixture was filtered, and an aliquot of the reaction mixture was diluted with 50% MeOH in H_2_O and analyzed with mass spectrometry without quenching with DTT. Mass analysis showed the corresponding masses of the starting material (**1**) and the water addition product (**2**) (Figure 2B(III)). Together, these observations of the different intermediates with and without gold support the proposed mechanism. The observation of gold intermediates in the absence of TEAB could be attributed to the crucial role of TEAB in taking the reaction to the next step for cyclization.

### Scope of the Gold-Mediated Cyclization

**Figure 3:**
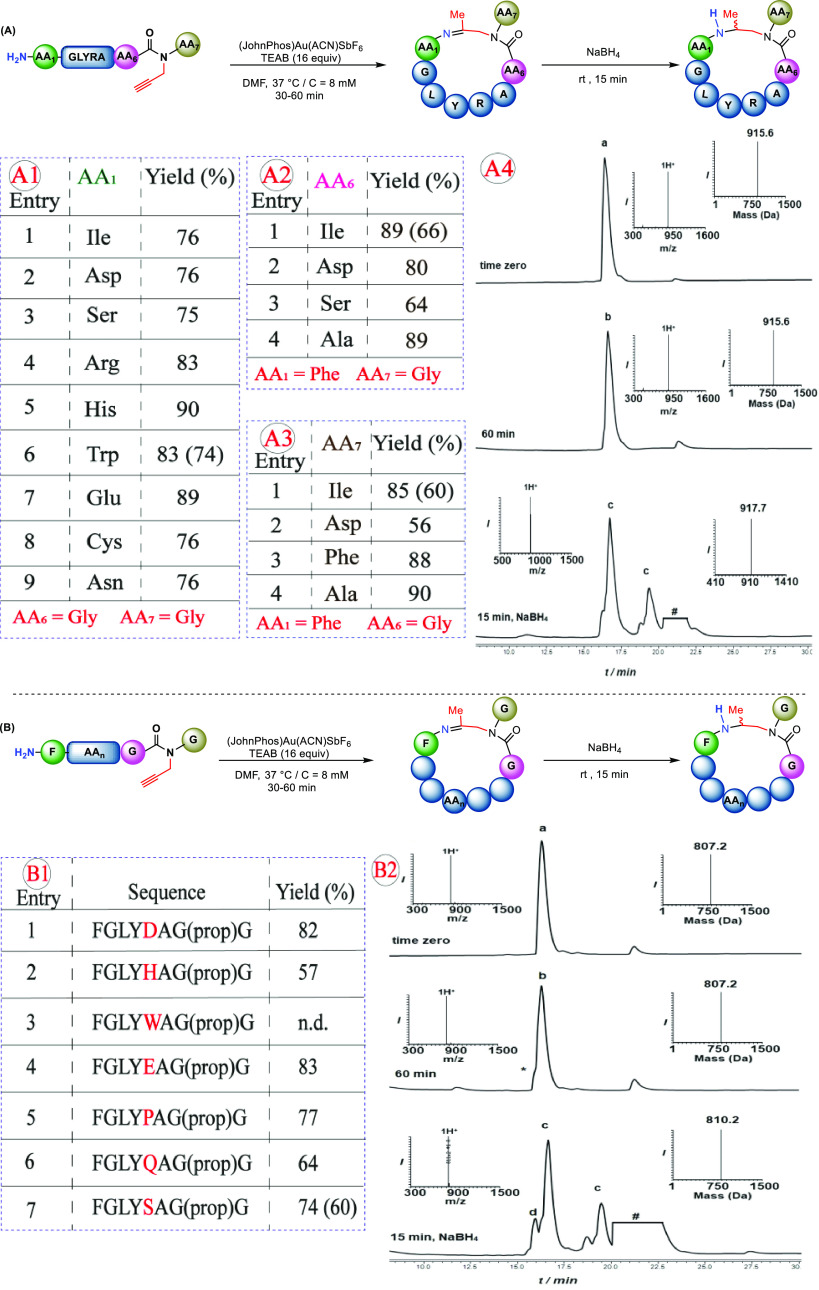
Substrate scope: (A) Scope of different cyclization junctions: (A1) Scope of the *N*-terminal amino acid. (A2) Cyclization at AA–Gly junctions. (A3) Cyclization at Gly–AA junctions. (A4) Analytical HPLC–mass analysis for the cyclization reaction: peak a corresponds to the starting peptide **W**GLYRAG(prop)**G** with the observed mass of 915.6 ± 0.0 Da (calcd 916.0 Da); peak b corresponds to the cyclized product with the observed mass of 915.6 ± 0.0 Da (calcd 916.0 Da); peak c corresponds to the reduced cyclized products (diastereomers), with the observed mass of 917.7 ± 0.0 Da (calcd 918.0 Da). (B) (B1) Scope of cyclization of different AA in the middle of the sequence. (B2) Analytical HPLC–mass analysis for the cyclization reaction: peak a corresponds to the starting peptide FGLY**S**AG(prop)G with the observed mass of 807.2 ± 0.0 Da (calcd 807.8 Da); peak b corresponds to the cyclized product with the observed mass of 807.2 ± 0.0 Da (calcd 807.8 Da); peak c corresponds to the reduced cyclized products (diastereomers) with the observed mass of 810.2 ± 0.0 Da (calcd 809.8 Da). Yields were based on LC-MS analysis of the crude reaction mixture. * refers to partial hydrolysis of the imine during HPLC analysis. # refers to the metal complex. n.d. = yield is not determined. Isolated yields of the reduced cyclic peptides were in parentheses.

To investigate the impact of different amino acids on cyclization efficacy, three distinct model peptide libraries were prepared. The first is made up of different *N*-terminal amino acids (H_2_N-**AA1**-GLYRAG(prop)G) (**AA1** = Ile, Asp, Ser, Arg, His, Trp, Glu, Cys, and Asn) (Figure 3A1). All of these peptides were cyclized under our optimal conditions, and the cyclic products were obtained in moderate to good conversion yields (75–89%). While amino acids such as Ile and Arg exert steric hindrance (Figure 3A1, entries 1 and 4), Asp, Ser, His, Trp, Glu, and Cys could coordinate with the gold and hamper the reaction efficiency (Figure 3A1, entries 2, 3, 5–9). Notably, none of these amino acids had a significant impact on the reaction outcome, demonstrating the cyclization method’s broad tolerance (Figure 3A1).

Subsequently, we proceeded to look into the influence of the propargylation sites on the cyclization step (Figure 3A2), assuming that steric factors and conformational differences at this position could affect cyclization. As a result, model peptides with different amino acids containing a propargylated amide at AA–Gly junctions (H_2_N-FGLYRA-**AA6**-(prop)G) (**AA6** = Ile, Asp, Ser, and Ala) were synthesized and tested. Subjecting all of these peptides to our optimized conditions led to the cyclized products in 64–89% conversion yields (Figure 3A2, entries 1–4). In general, amino acids having bulky groups such as Ile exert steric hindrance, whereas amino acids having reactive functional groups such as Asp and Ser could coordinate with the metal, therefore influencing the reaction. However, none of these factors appeared to have had a significant impact on the reaction, and the reaction yield remained acceptable even for the most affected one, e.g., Ser–Gly junction (Figure 3A2, entry 3). This observation is probably due to the strong coordination character of the alkyne group toward Au(I), which might be difficult to compete with by other side chains, therefore keeping the reaction with minimal interference.

The effect of different propargylation sites, such as Gly–AA, on the cyclization reaction was also investigated (H_2_N-FGLYRAG(prop)**AA7**); (**AA7** = Ile, Asp, Phe, and Ala). The peptides with amino acids Ile, Asp, Phe, and Ala at the propargylation site were prepared and tested (Figure 3A3). Our findings revealed that sterically hindered amino acids such as Ile and Phe worked well and produced the desired products in 85 and 88% conversion yields, respectively (Figure 3A3, entries 1 and 3). Furthermore, a model peptide containing Asp, as a potentially metal-coordinating group, was cyclized to give the desired product in 56% conversion yield (Figure 3A3, entry 2).

We likewise investigated the effect of different amino acids in the middle of the sequence, which might affect the ring closure. The selected model peptides were tested, each with a different amino acid in the middle of the sequence (Asp, His, Trp, Glu, Pro, Gln, and Ser). All reactions went smoothly, yielding the cyclized products in 57–83% conversion yields (Figure 3B1, entries 1–7).

**Figure 4:**
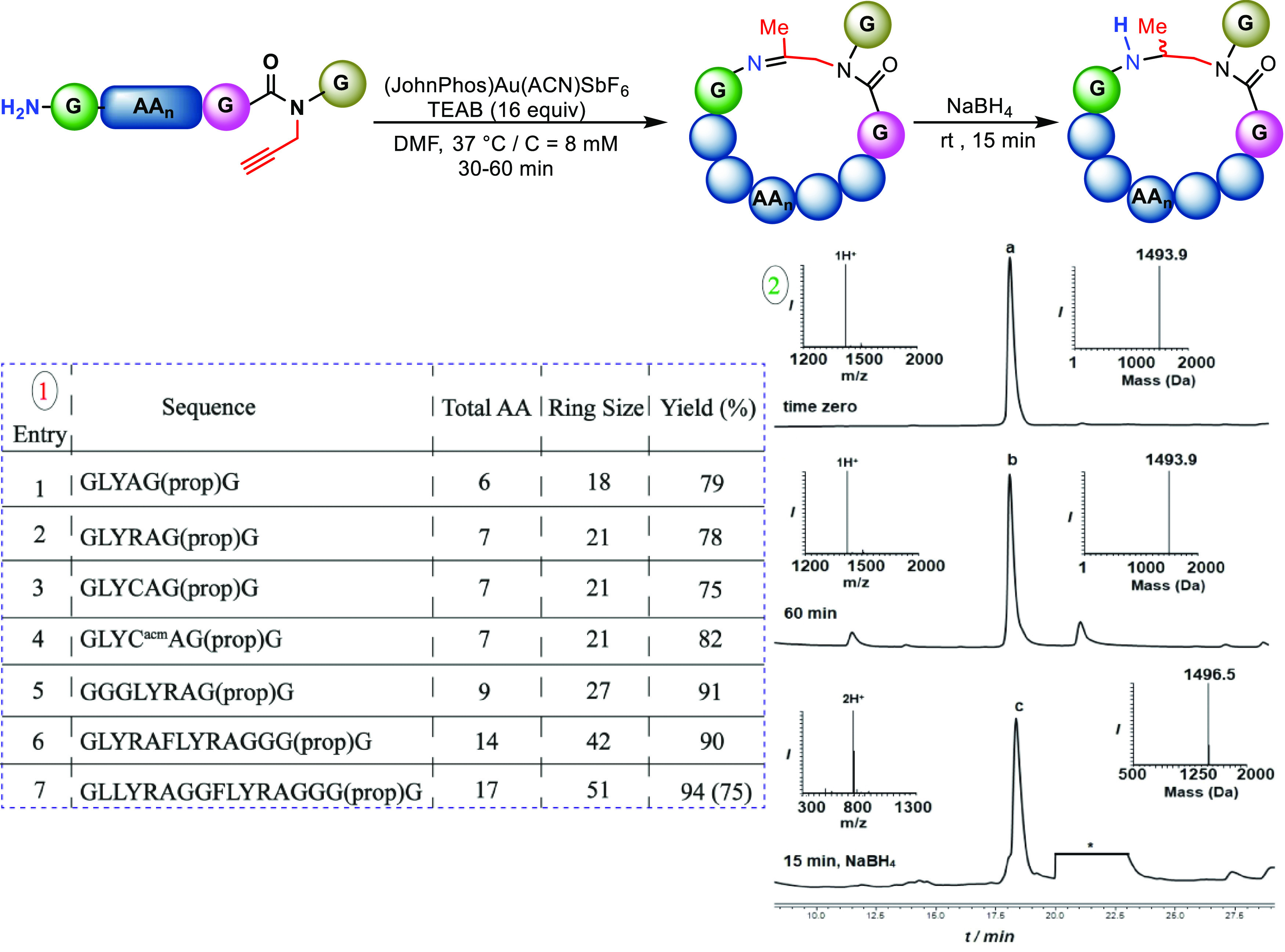
Substrate scope: (1) Scope of cyclization of peptides with various lengths. (2) Analytical HPLC–mass analysis for the cyclization reaction: peak a corresponds to the starting peptide GLYRAFLYRAGGG(prop)G with the observed mass of 1493.9 ± 0.0 Da (calcd 1494.6 Da); peak b corresponds to cyclized product with the observed mass of 1493.9 ± 0.0 Da (calcd 1494.6 Da); peak c corresponds to the reduced cyclized product with the observed mass of 1496.5 ± 0.0 Da (calcd 1496.6 Da). Yield based on LC-MS analysis of the crude reaction mixture. Isolated yield of the reduced cyclic peptides was in parentheses. * refers to the metal complex.

At this stage, we aimed to test the effect of the chain’s length on the cyclization reaction (Figure 4). We therefore prepared model peptides with sequences of 6–17 amino acids, which upon cyclization would cover ring sizes ranging from 18 to 51 atoms. Our study revealed that the length of the peptide had no effect on the efficacy of the cyclization reaction. The peptide with six amino acids (18-membered ring) produced the cyclic product with a conversion yield of 79% (Figure 4, entry 1). The peptide with seven amino acids (GLYRAG(prop)G, GLYCAG(prop)G, GLYC^acm^AG(prop)G) gave the cyclic product in 78, 75, and 82% conversion yields, respectively (Figure 4, entries 2–4). Model peptides of 9, 12, and 17 amino acids also performed well in the reaction, yielding cyclized products in 91, 90, and 94% conversion yields, respectively (Figure 4, entries 5–7). Notably, the Acm-protected group on the Cys side chain was stable during these reaction conditions, in contrast to our previous report using aqueous formaldehyde and the DMF/dioxane solvent system.^27^

**Figure 5:**
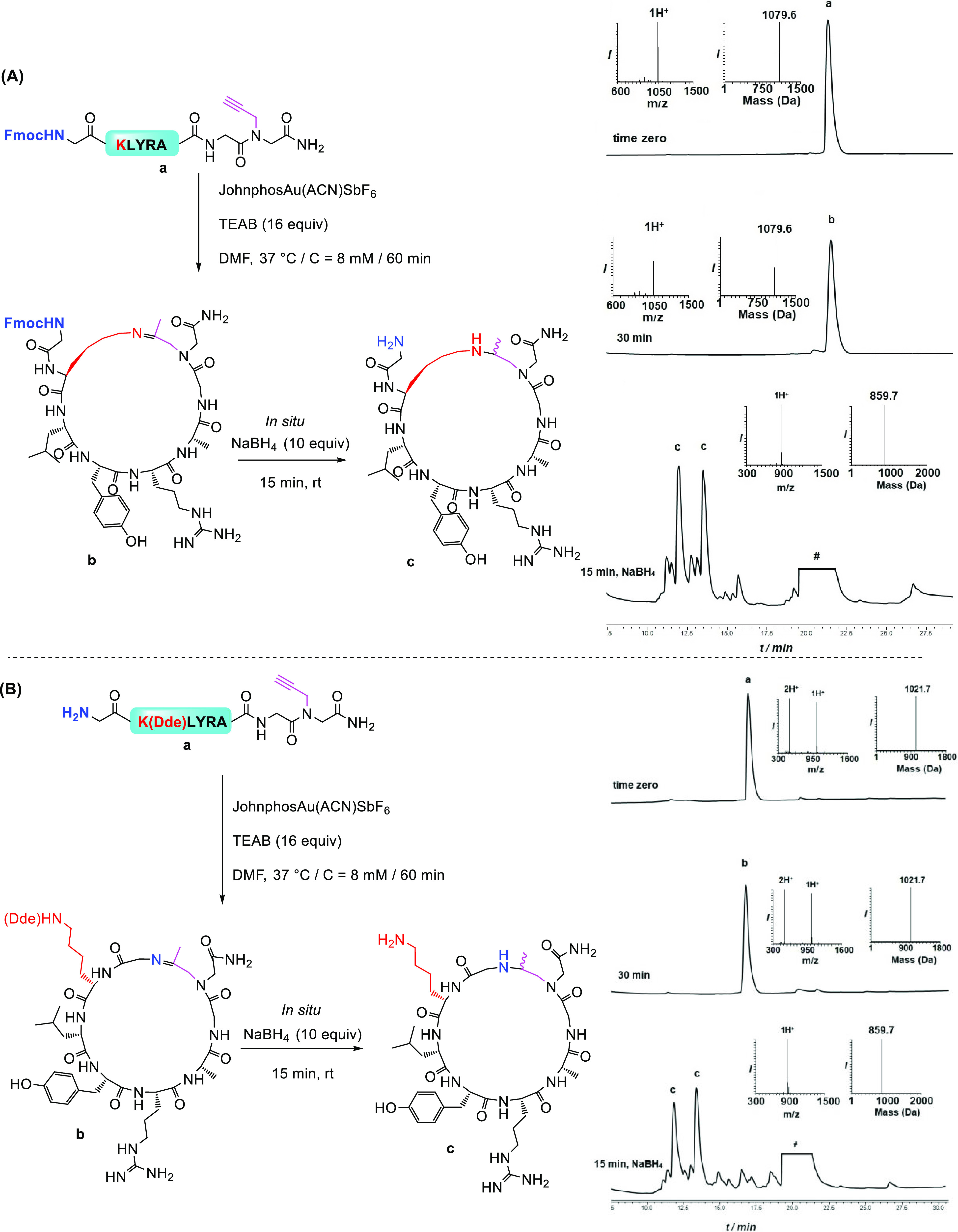
(A) Cyclization of Fmoc-GKLYRAG(prop)G model peptide. (Right) Analytical HPLC–mass analysis for the reaction of Fmoc-GKLYRAG(prop)G. Peak a corresponds to the starting peptide with observed mass of 1079.6 ± 0.0 Da (calcd 1079.9 Da); peak b corresponds to the cyclized product with the observed mass of 1079.6 ± 0.0 Da (calcd 1079.9 Da); and peak c corresponds to the reduced cyclized products (diastereomers) with the observed mass of 859.7 ± 0.0 Da (calcd 859.9 Da). Isolated yield of the reduced cyclic peptide of 61%. (B) Cyclization through *N*-terminal amine. (Right) Analytical HPLC–mass analysis for the cyclization reaction of GK(Dde)LYRAG(prop)G. Peak a corresponds to the starting peptide with the observed mass of 1021.7 ± 0.0 Da (calcd 1021.9 Da); peak b corresponds to the cyclized product with the observed mass of 1021.7 ± 0.0 Da (calcd 1021.9 Da); and peak c corresponds to the reduced cyclized products (diastereomers) with the observed mass of 859.7 ± 0.0 Da (calcd 859.9 Da). # refers to the metal complex.

To study the scope of the gold-mediated cyclization of Lys-containing peptides, we looked at a model peptide with a free Lys residue in its sequence, which could compete with the N-terminus amine in the cyclization reaction. When two model peptides, either with a dimethylated *N*-terminus amine or with a dimethylated ε-amine on the Lys side chain, were examined, we found that both the peptides reacted with similar kinetics. To direct the cyclization step, we decided to protect the *N*-terminus amine or the Lys side chain. Therefore, we prepared a model peptide with Fmoc-*N*-terminally protected amine (Fmoc-GKLYRAG(prop)G) and exposed to gold-mediated cyclization. We obtained the cyclized product through the Lys side chain with the Fmoc protecting group left intact. However, during the reduction step, the Fmoc protecting group was fully removed (Figure 5A).

Next, we constructed a model peptide with a Dde protecting group, GK(Dde)LYRAG(prop)G, and exposed it to our conditions to produce a cyclic peptide via the *N*-terminus amine (Figure 5B). Also, here, the Dde protecting group was removed during the reduction step.

### Asymmetric Reduction

**Figure 6:**
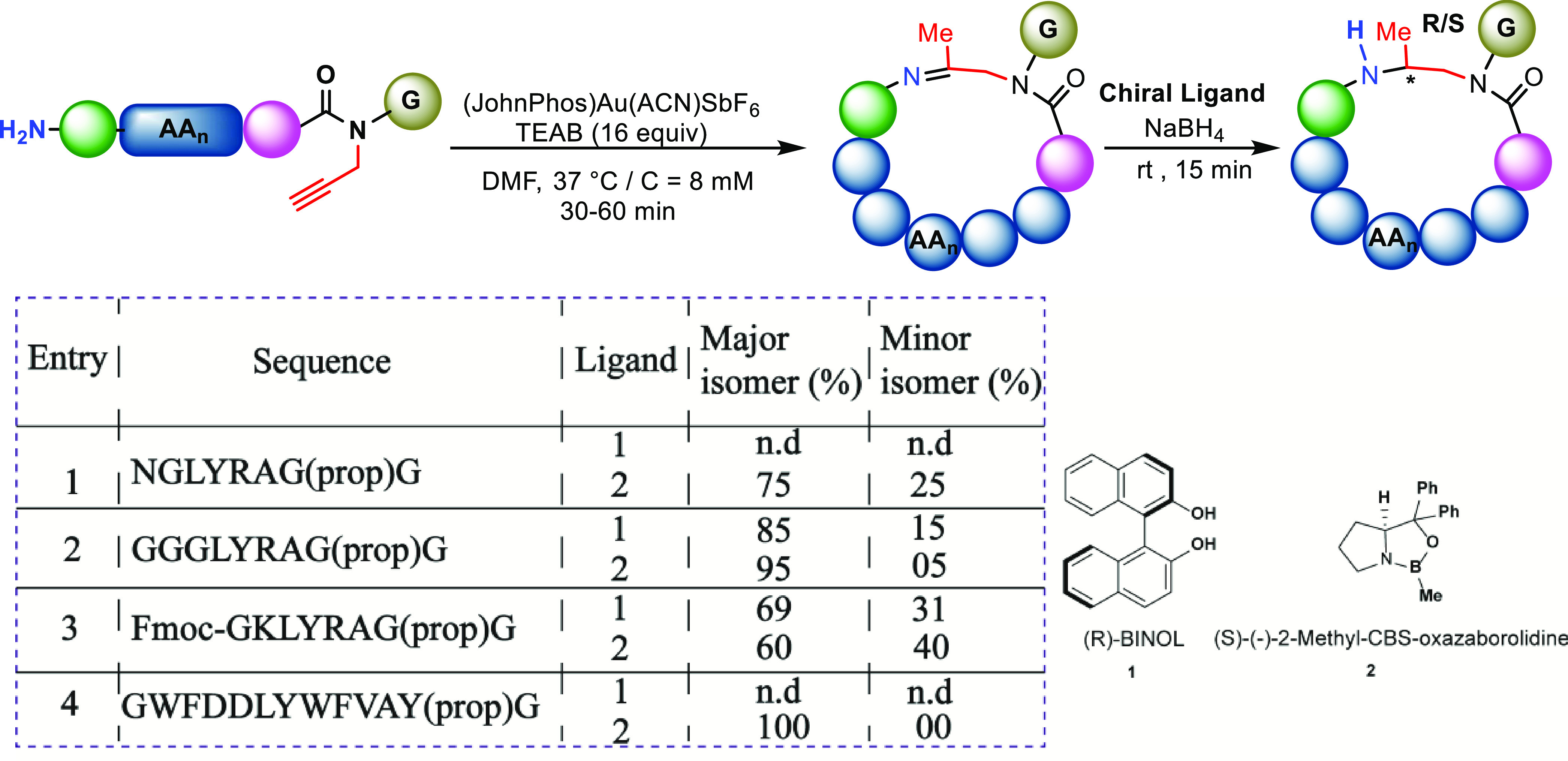
Examining chiral reduction of the imine cyclic peptides. The major and minor isomer ratios are referred to the corresponding peaks in the achiral version. n.d = not determined. (JohnPhos)Au(ACN)SBF_6_ (2.0 equiv) and chiral ligand (3.0 equiv).

During the reduction with NaBH_4_, we observed the formation of two isomers with different diastereomeric ratios depending on the sequences. For example, in the case of NGLYRAG(prop)G, the diastereomeric ratio was 63:37, whereas for the GKLYRAG(prop)G sequence, the ratio was 50:50. Although we were unable to determine the absolute configuration, we started to investigate the possible stereoselective reduction of the imine using chiral ligands. For this, we chose R-(+)-1,1′-Bi(2-naphthol), (R-BINOL), and S-(−)-2-Methyl-CBS-oxazaborolidine, (S-CBS), ligands to study the diastereoselectivity of four sets of peptides in which achiral reductions with minimal or no selectivity were observed (Figure 6, entries 1–4). In the case of peptide NGLYRAG(prop)G, where the ratio was 63:37 in achiral reduction, this has increased to 75:25 in the presence of ligand S-CBS (Figure 6, entry 1). Similarly for the peptide GGGGLYRAG(prop)G, we could achieve up to 95:05 diastereoselectivity with S-CBS and 85:15 with R-BINOL (Figure 6, entry 2). For the peptide GKLYRAG(prop)G, which showed no selectivity in achiral reduction, we observed an increase of up to 70% of the product corresponding to the first HPLC peak in the presence of R-BINOL (Figure 6, entry 3). Interestingly, nearly prefect selectivity was observed in the case of GWFDDLYWFVA(prop)Y in the presence of the S-CBS ligand (Figure 6, entry 4).

### Synthesis and Study of Cyclic Peptide Modulators of Ubiquitin Chains

**Figure 7:**
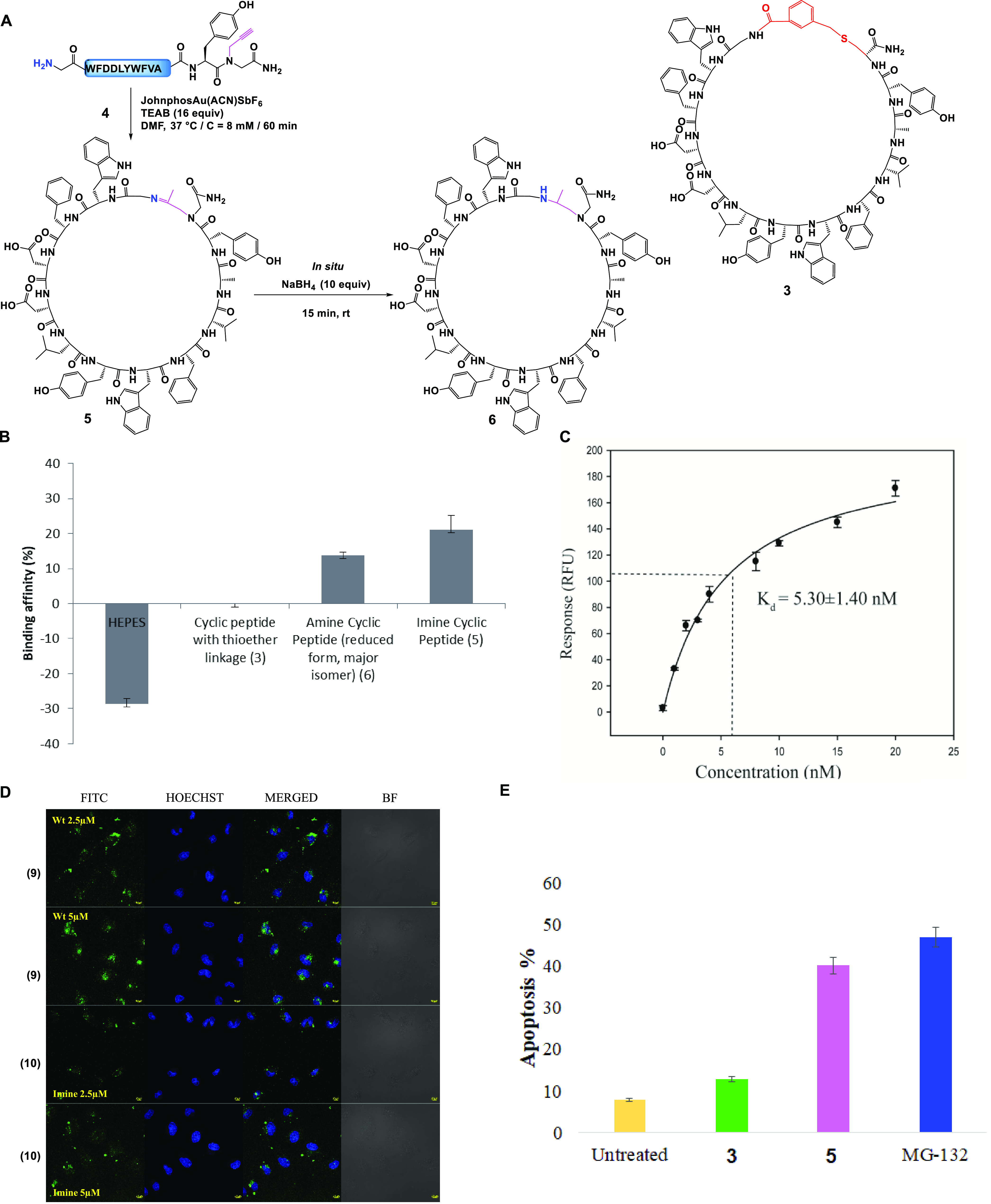
(A) Cyclization of peptide **4** to cyclic peptides **5** and **6** (left) and structure of **3** (right). (B) Binding of cyclic peptides to Lys48-di-Ub, normalized to the affinity of **3**. HEPES buffer (50 mm HEPES, 150 mm NaCl, 0.1% Tween, pH 7.3; negative control). All measurements were performed in triplicate. Error bars represent standard error. (C) Binding curve of FITC-labeled **10** to Lys48-di-Ub. The *K*_d_ value of 5.30 ± 1.40 nm was determined. All measurements were performed in triplicate. (D) Live-cell uptake of cyclic peptides **9** and **10** in 2.5 and 5.0 μM: (Left to right) FITC signal from the cyclic peptides **9** and **10**, respectively; Hoechst signals from live cells; Hoechst and FITC signals merged and bright-field images. The experiment was repeated in duplicates (scale bar: 10 μm). (E) Induction of apoptosis in HeLa cells by cyclic peptides **3** and **5** and MG-132 (bars represent standard error) (2.5 μM concentration).

Novel cyclic peptide analogues with varied linkages may have an impact on the peptide’s physicochemical and pharmacological properties.^36^ We wanted to apply our method to check the activity and permeability of known cyclic peptides that bind to Ub chains and modulate their properties. Therefore, we prepared the propargylated version of our previously reported peptide having the thioether linkage.^37^ The peptide GWFDDLYWFVAY(prop)G, **4**, was subjected to the gold-mediated reaction to obtain the corresponding imine cyclic peptide **5** and its amine reduced form **6** (major isomer) (Figure 7A).

With both peptides in hand (**5** and **6**, Figure 7A), we investigated the binding efficiency using our fluorescence-based competitive assay.^38^ We observed a ∼22% increase in the binding affinity for Lys48-linked di-Ub chains for the imine-cyclized product (**5**) and ∼15% for its reduced cyclic product (**6**), compared to cyclic peptide having the thioether linkage (**3**) (Figure 7B), and the *K*_d_ was determined to be 5.30 ± 1.4 nM (Figure 7C). Encouraged by these *in vitro* results, we explored the cellular uptake and apoptosis efficacy of the new cyclic peptide in living cells. To investigate the live cell delivery efficacy of the FITC-labeled peptides (**9** and **10**), both cyclic peptides were incubated for 1h with HeLa cells at different concentrations of 2.5, 5, 7, and 10 μM. Upon analyzing the confocal images for both peptides **9** and **10**, with only 2.5 μM concentration, the cells have shown significant fluorescence, which indicates the cellular uptake of these peptides (Figure 7D). After assessing the cellular uptake, we probed the controlled cell death or apoptosis in HeLa cells using only 2.5 μM peptides (**3** and **5**) and the known proteasome inhibitor MG-132 as a positive control. With cyclic peptide, **5**, upon 24 h of treatment, the cancer cells (HeLa) showed up to threefold higher induction of apoptosis than that of peptide **3** or the cyclic peptide with the keto linkage^27^ (Figure 7E). Interestingly, the apoptosis induction efficacy of peptide **5** is similar to that of the commercially available MG-132 peptide.

## Conclusions

We have developed an efficient and straightforward method for obtaining structurally rigid macrocyclic peptides using the gold(I) complex bearing an imine functionality as a nonpeptidic element. The reaction was carried out without the presence of side chain protecting groups and compatible to a variety of proteinogenic functional groups. Stereoselective reduction of the cyclic imine was achieved using chiral R-Binol and S-CBS ligands. The applicability of this method was demonstrated by the development of the cyclic peptide modulator for Lys48-linked di-Ub chains with improved binding and apoptosis compared to the parent compound.

Peptide-based drug discovery has witnessed a significant upturn within the past decade, which is in part due to the introduction of unnatural elements that are allowing us to overcome some of the drawbacks associated with peptide therapeutics. In our case, changing the nature of the linkage using the imine cyclization method led to a threefold increase in the apoptosis of these cyclic peptides. We envision that our approach could serve as a platform for synthesizing other therapeutically relevant peptides with unnatural elements by further modifying the imine moiety.

## Supporting Information Available

Experimental data, characterization data, materials, peptide synthesis, experimental methods, cyclization reactions, HPLC and mass spectrometry analyses, and NMR data (PDF)
