# Supplementary material for: Gold(I)-Mediated Rapid Cyclization of Propargylated Peptides via Imine Formation
Source: J Am Chem Soc. 2022 Mar 8;144(11):4966–76. doi: 10.1021/jacs.1c12906 (PMC8949771; doi:10.1021/jacs.1c12906)
Supplement: Supplementary file 1 — ja1c12906_si_001.pdf [file ja1c12906_si_001.pdf]

Supporting Information

Gold(I)-Mediated Rapid Cyclization of Propargylated Peptides via Imine Formation

Rajeshwer Vanjari, Deepanjan Panda, Shaswati Mandal, Ganga B. Vamisetti, and Ashraf Brik*

Schulich Faculty of Chemistry, Technion-Israel Institute of Technology, Haifa 3200008, Israel.

*E-mail: abrik@technion.ac.il

Table of Contents **Page**

Materials ……..........……….…………………………………………………...……….....	S1
General methods ………………………………………………….....………...…........	S2
General procedure for synthesis of AA-G(prop)G……………………..……………..........	S3
Synthesis of AA-G(prop)-AA peptides …………………………………………………	S4
General procedure for Au(I)-mediated cyclization reaction ………………………….....	S4
Scope of Au(I)-Mediated cyclization …….…………………..	S5
Synthesis of $(Me)_2$N-GLYAG(prop)G & $(Me)_2$N-GLYKRAG(prop)G peptides ...	S37
Scope of lysine containing peptides ……………………………………………......	S38
Synthesis of thioether linked peptide **3** ……………………………………….…	S39
Synthesis of propargylated peptide **4** ...……………………………………………...	S39
Cyclization of propargylated peptide **4** using gold to cyclic peptides **5** and **6** with Au(I)	S40
Synthesis of peptide **4a** …………………………………………………………………	S41
Cyclization of propargylated peptide **4a** to cyclic peptide **7** with Au(I)………………..	S42
Synthesis of thioether linked peptide **3a** ……………………………………………….	S43
Synthesis of cyclic peptide **10** ……………………………………….………………	S43
Au(I) mediated on resin reaction (Solid Phase Cyclization)...…………………………	S43
Fluorescence based competitive assay against K48-Ub-Di-Ub chains …….....	S43
Kd determination ……………………………………………………..........................	S44
Cell uptake studies………………………………………………………………………	S45
Apoptosis studies ………………………………………………………................	S45
Reference………………………………………………………………………………	S46
NMR data ……………………………………………………………………………	S47

Experimental Section

Materials

Solid Phase Peptide Synthesis (SPPS) was carried out manually in syringes, equipped with Teflon filters, purchased from Torviq or by using an automated peptide synthesizer (CS336X, CSBIO). Analytical grade *N*, *N*-Dimethylformamide (DMF), dichloromethane (DCM), and Trifluoroacetic acid (TFA) were purchased from BioLab. Diethyl ether and HPLC-grade acetonitrile (ACN) was purchased from Avantor. Triisopropyl silane (TIPS) was purchased from Sigma-Aldrich. Commercial reagents were used without further purification. Resins were purchased from CreoSalus and all protected amino acids were purchased from GL Biochem. The activating reagents [(2-(1Hbenzotriazol-1-yl)-1,1,3,3tetramethyluronium hexafluorophosphate (HBTU), hydroxybenzotriazole (HOBt), [(6chlorobenzotriazol-1-yl)oxy(dimethylamino)methylidene]-dimethyl-azanium hexafluorophosphate (HCTU), 1-[Bis(dimethylamino)methylene]-1H-1,2,3-triazolo[4,5-b]pyridinium 3-oxid hexafluorophosphate (HATU), were purchased from Luxembourg Bio Technologies. Bromoacetic acid, propargylamine, and propargyl bromide were purchased from Alfa Aesar. $PdCl_2$, AuCl, and chloroacetic acid were purchased from Acros Organics. *N*,*N′*-diisopropylcarbodiimide (DIC) was purchased from Kemilab Organics. (JohnPhos)Au(ACN)SbF_6, *m*-chlorobenzoic acid, and dimethylamine (40% in water), were purchased from Merck. Fmoc-Lys(Dde)-OH was purchased from Holland Moran. Rink amide resin (100-200 mesh, 0.26 mmol/g) was purchased from CreoSalus.

HPLC was performed on a Thermo instrument (Dionex Ultimate 3000) using analytical Thermo Scientific (Hypersil Gold, C18, 3 µm, 4.6 × 150 mm) columns at flow rate of 1.2 ml/min. Preparative HPLC was performed on a Thermo Scientific instrument ultimate 3000 using Waters XSelect C18 (10µm, 19 × 250 mm) and semi preparative HPLC was performed on a Thermo Scientific instrument (Spectra System SCM1000) using XBridge BEH300 C4 (10 µm, 150 × 10 mm) and Jupiter C4 (10 µm, 300 Å, 250 × 10 mm) column, at flow rate of 15 and 4 mL/min

respectively. All synthetic products were purified by HPLC and characterized by mass spectrometry using LCQ Fleet Ion Trap (Thermo Scientific). All calculated masses have been reported as an average isotope composition. Buffer A: 0.1% TFA in water; buffer B: 0.1% TFA in acetonitrile. (Method: 0-60% B in 33 min).

List of the protected amino acids used in peptides synthesis: Fmoc-Gly-OH, Fmoc-Ala-OH, Fmoc-Leu-OH, Fmoc-Ile-OH, Fmoc-Phe-OH, Fmoc-Pro-OH, Fmoc-His(Trt)-OH, Fmoc-Asn(Trt)-OH, Fmoc-Gln(Trt)-OH, Fmoc-Arg(Pbf)-OH, Fmoc-Lys(Boc)-OH, Fmoc-Tyr(tBu)-OH, Fmoc-Ser(tBu)-OH, Fmoc-Asp(OtBu)-OH, Fmoc-Glu(OtBu)-OH, Fmoc-Trp(Boc)-OH, Fmoc-D-Trp(Boc)-OH, Fmoc-Cys(Acm)-OH, Fmoc-Lys(Dde)-OH.

General methods

Coupling of amino acids: The Coupling of the amino acids (AA) was performed on automated/manual peptide synthesizer in presence of 4 equiv. of AA, 4 equiv. of HCTU/HATU and 8 equiv. of DIEA to the initial loading of the resin (Knorr Resin (0.26 mmol/g)), and the reaction was left at room temperature for 60 min.

Cleavage of the resin

After finishing the coupling reactions, the resin was washed with (3x5ml) DMF, (3x5ml) MeOH, and (3x5ml) DCM and vaccum dried. The cleavage cocktail of 95% (v/v) TFA, 2.5% (v/v) H_2O, and 2.5% (v/v) TIPS was added to the resin and kept on shaker for 1.5 hr. Then the resin was removed by filtration and was washed with additional TFA (2×1 mL). The combined filtrate was added dropwise to cold ether followed by centrifugation, decanting of ether and by dissolution of residue in acetonitrile/water for freeze-drying in the lyophilizer afforded the desired peptide.

General Procedure for synthesis of AA-G(prop)G

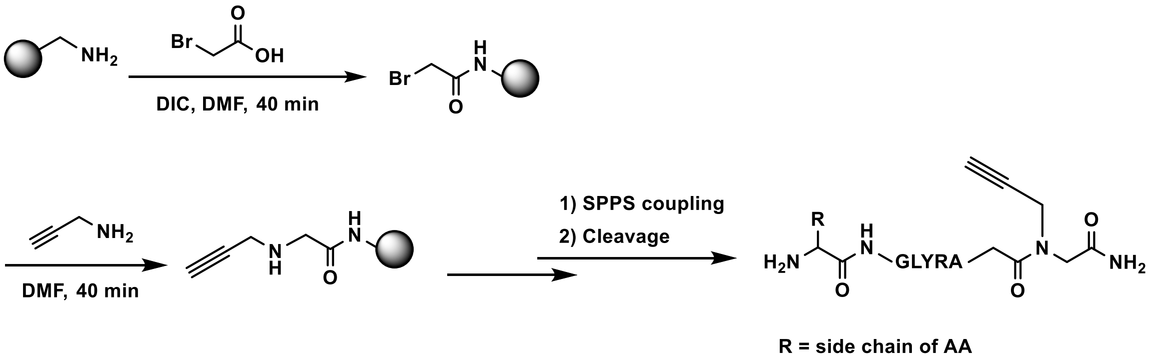

The resin, after peptide chain synthesis, was treated with 20% piperidine in DMF for Fmoc-deprotection (3x5ml). BAA (20 equiv.) was activated using 20 equiv. DIC in DMF for 20 min at room temperature. Then, activated BAA was added to the resin and allowed it to couple for 40 min. Subsequently, propargyl amine (20 equiv.) in DMF (900 µl, ~1 M) was added and the reaction left for 40 min at room temperature. Then the next amino acid was double coupled using 20 equiv. Fmoc-Gly-OH, 20 equiv. HATU and 40 equiv. DIEA. The coupling of remaining amino acids was continued as described above. Finally, the peptides were cleaved from the resin with the cleavage cocktail and purified using preparative HPLC C18 column with a gradient of 10-50%B over 40 min.

Synthesis of AA-G(prop)-AA peptides

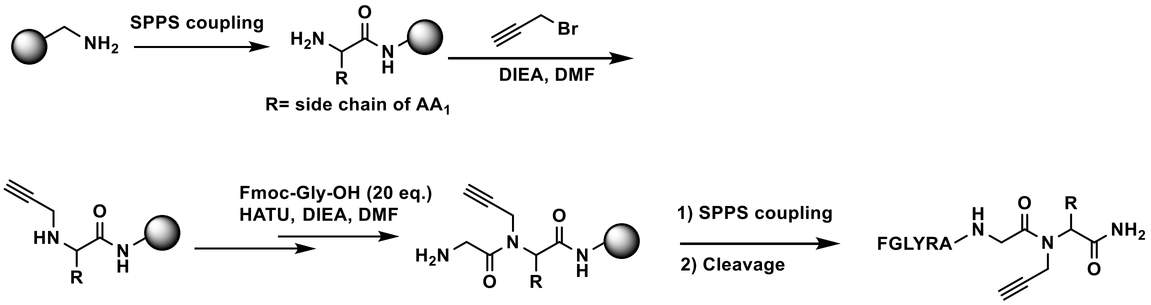

The first amino acid is coupled according to the general coupling method described above. Following that, DIEA (2 equiv.) in DMF was added to the resin, then propargyl bromide (25 equiv.) in DMF was added. The reaction was left on a plate shaker for 1 hr. The next AA, after propargyl installation step, was coupled doubly using 20 equiv. HATU with 40 equiv. DIEA for 30 min for each cycle (X2). Then the next remaining amino acids were coupled using peptide synthesizer as described above.

General procedure for Au(I)-mediated cyclization reaction

To the solution of a peptide (0.1 mg, 1.0 equiv.) and tetraethylammonim bromide (TEAB) (16.0 equiv.) in DMF (8 mM) was added JohnPhosAu(ACN)SbF_6 complex (2.0 equiv.) in eppendorf. The reaction was incubated at 37 °C for 30-60 min and followed by reduction with $NaBH_4$ (10.0 equiv.) for 15 min. The reaction was quenched with excess DTT (dithiothreitol) and monitored by analytical HPLC and ESI mass analysis. For asymmetric reduction, we added the chiral ligand (3.0 equiv.) and $NaBH_4$ (10.0 equiv.) after upon reaction completion and kept for 15 min at room temperature. Conversion yields were calculated from the peak areas by integrating all the peaks in chromatogram. Conversion yields of the imine cyclic products were calculated indirectly from the peak area of the reduced cyclic peptides. This because the retention time of the imine and the starting material are the same, while the reduced has different retention time that allow us to determine conversion yield of the imine product more accurately.

Scope of Au(I)-Mediated cyclization

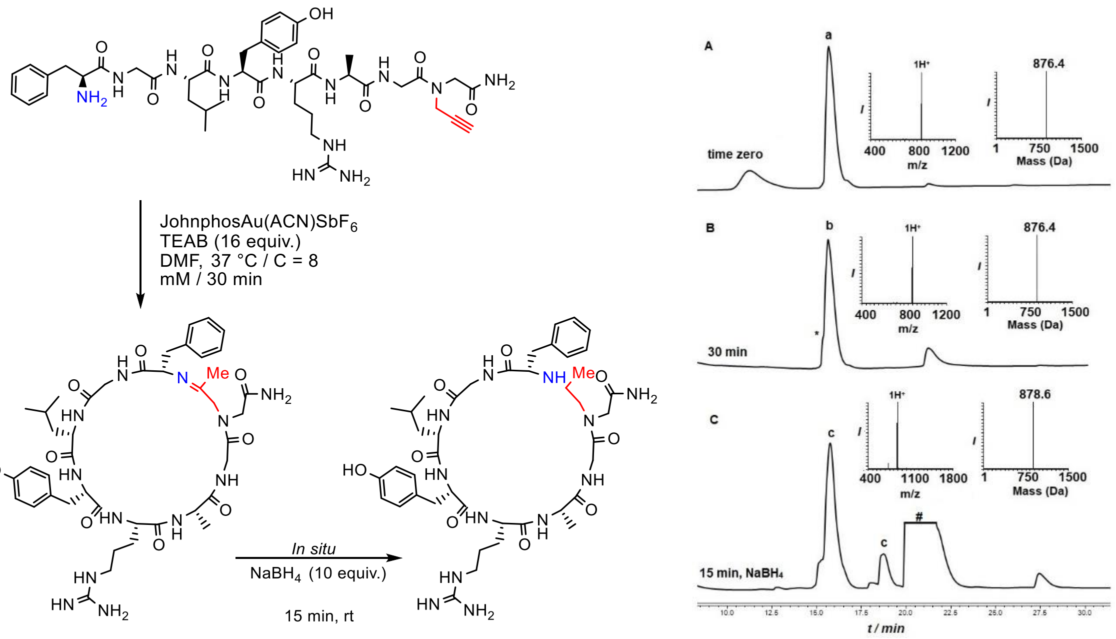

Figure S1: FGLYRAG(prop)G peptide reaction with JohnPhosAu(ACN)SbF_6: (A) Analytical HPLC and mass analysis of pure FGLYRAG(prop)G . Peak a corresponds to FGLYRAG(prop)G with the observed mass 876.4 ± 0.0 Da (calcd 876.9 Da) (r.t = 15.59 min). (B) Analytical HPLC and mass analysis after 30 min incubation at 37 °C with JohnPhosAu(ACN)SbF_6. Peak b corresponds to cyclization product with the observed mass 876.4 ± 0.0 Da (calcd 876.9 Da) (r.t = 15.58 min). (C) Analytical HPLC and mass analysis after 15 min incubation at rt with $NaBH_4$. Peak c corresponds to the reduced cyclized products (diastereomers) with the observed mass 878.6 ± 0.0 Da (calcd 878.9 Da). (*) partial hydrolysis of the imine during the column separation. (#) metal complex.

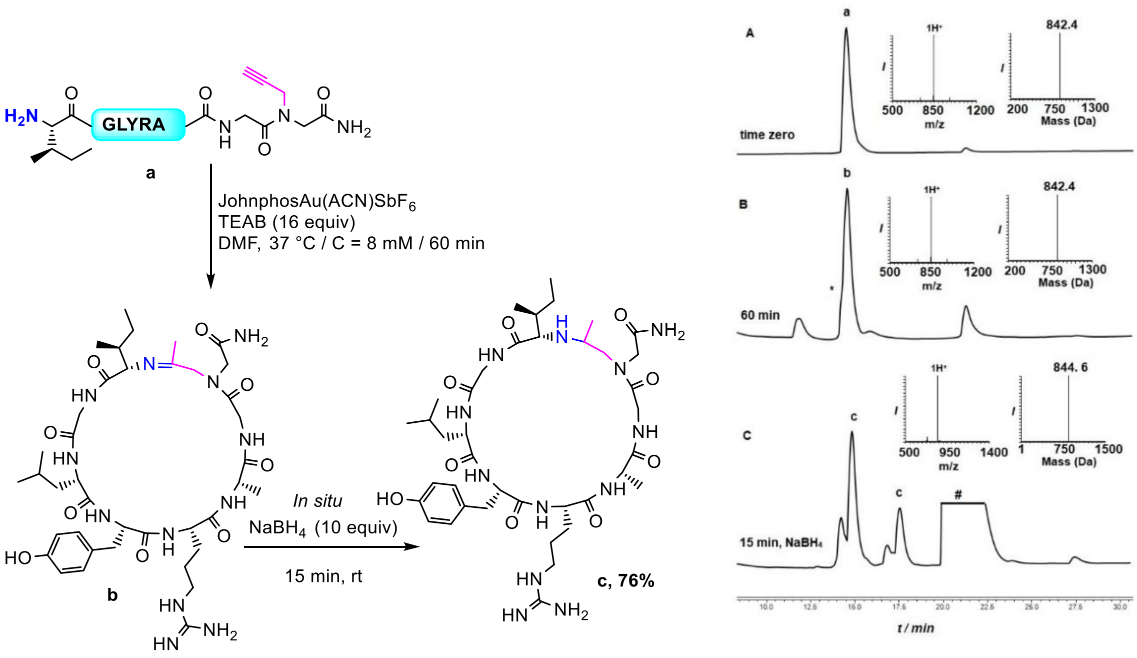

Figure S2: IGLYRAG(prop)G peptide reaction with JohnPhosAu(ACN)SbF_6: (A) Analytical HPLC and mass analysis of pure IGLYRAG(prop)G. Peak a corresponds to IGLYRAG(prop)G with the observed mass 842.4 ± 0.0 Da (calcd 842.9 Da) (r.t = 14.52 min). (B) Analytical HPLC and mass analysis after 60 min incubation at 37 °C with JohnPhosAu(ACN)SbF_6. Peak b corresponds to cyclization product with the observed mass 842.4 ± 0.0 Da (calcd 842.9 Da) (r.t = 14.51 min). (C) Analytical HPLC and mass analysis after 15 min incubation at rt with $NaBH_4$. Peak c corresponds to the reduced cyclized products (diastereomers) with the observed mass 844.6 ± 0.0 Da (calcd 844.9 Da). (*) partial hydrolysis of the imine during column separation. (#) metal complex.

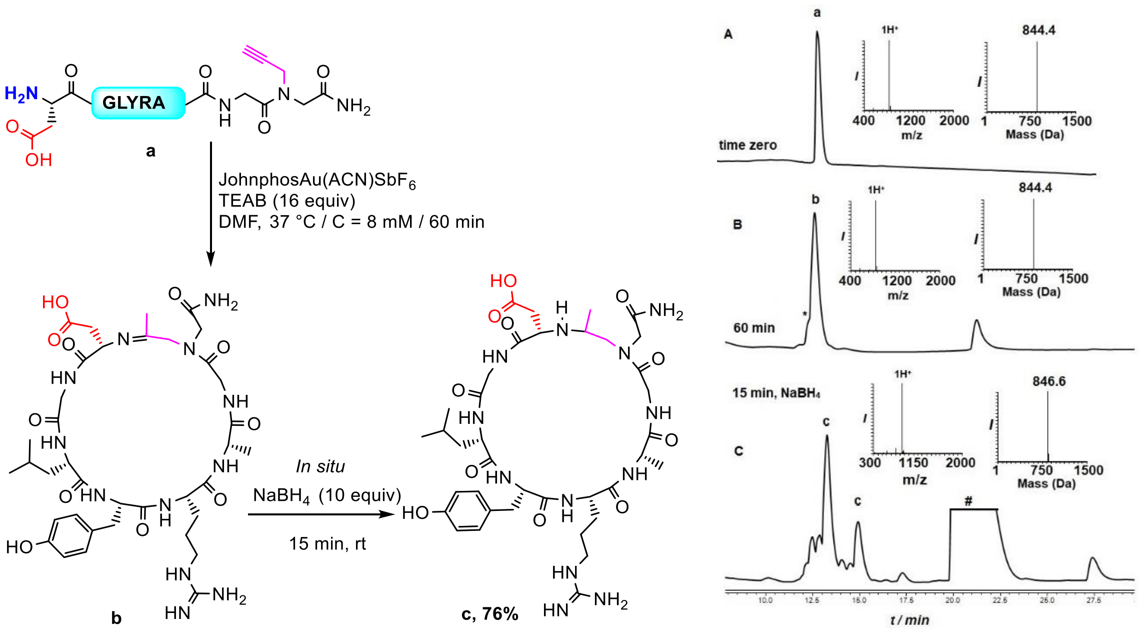

Figure S3: DGLYRAG(prop)G peptide reaction with JohnPhosAu(ACN)SbF_6: (A) Analytical HPLC and mass analysis of pure DGLYRAG(prop)G. Peak a corresponds to DGLYRAG(prop)G with the observed mass 844.4 ± 0.0 Da (calcd 844.9 Da) (r.t. = 12.67 min). (B) Analytical HPLC and mass analysis after 60 min incubation at 37 °C with JohnPhosAu(ACN)SbF_6. Peak b corresponds to cyclization product with the observed mass 844.4 ± 0.0 Da (calcd 844.9 Da) (r.t. = 12.62 min). (C) Analytical HPLC and mass analysis after 15 min incubation at rt with $NaBH_4$. Peak c corresponds to the reduced cyclized products (diastereomers) with the observed mass 846.6 ± 0.0 Da (calcd 846.9 Da). (*) partial hydrolysis of the imine during column separation. (#) metal complex.

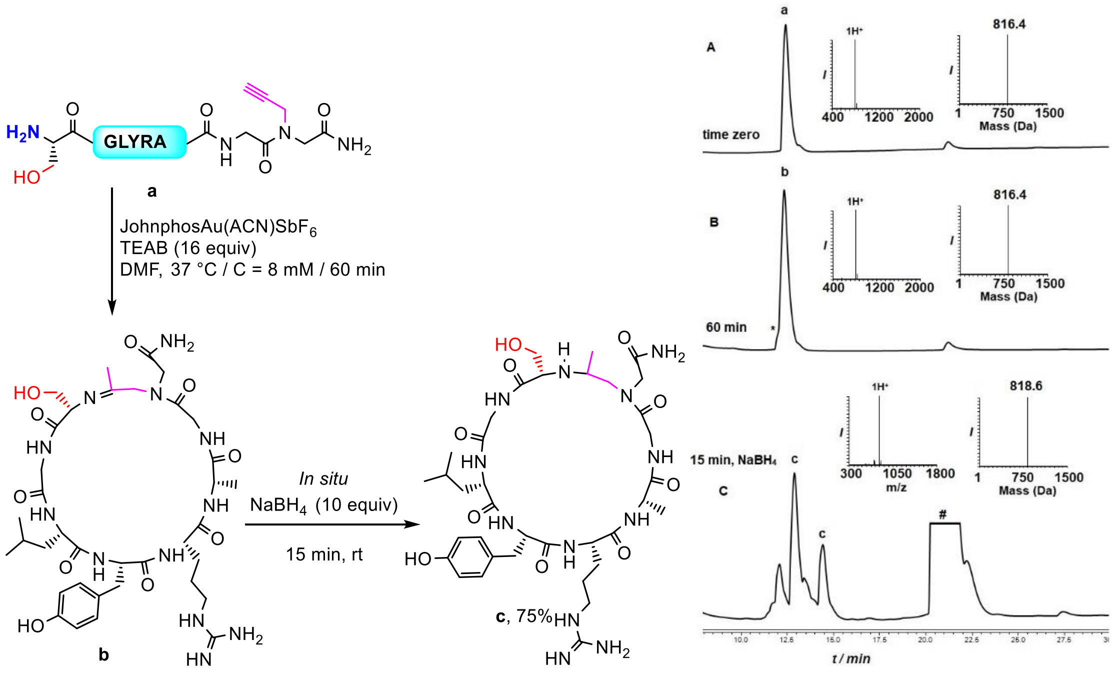

Figure S4: SGLYRAG(prop)G peptide reaction with JohnPhosAu(ACN)SbF$_6$: (A) Analytical HPLC and mass analysis of pure SGLYRAG(prop)G. Peak a corresponds to SGLYRAG(prop)G with the observed mass 816.4 ± 0.0 Da (calcd 816.9 Da) (r.t. = 12.45 min). (B) Analytical HPLC and mass analysis after 60 min incubation at 37 °C with JohnPhosAu(ACN)SbF6. Peak b corresponds to cyclization product with the observed mass 816.4 ± 0.0 Da (calcd 816.9 Da) (r.t. = 12.38 min). (C) Analytical HPLC and mass analysis after 15 min incubation at rt with NaBH$_4$. Peak c corresponds to the reduced cyclized products (diastereomers) with the observed mass 818.6 ± 0.0 Da (calcd 818.9 Da). (*) partial hydrolysis of the imine during column separation. (#) metal complex.

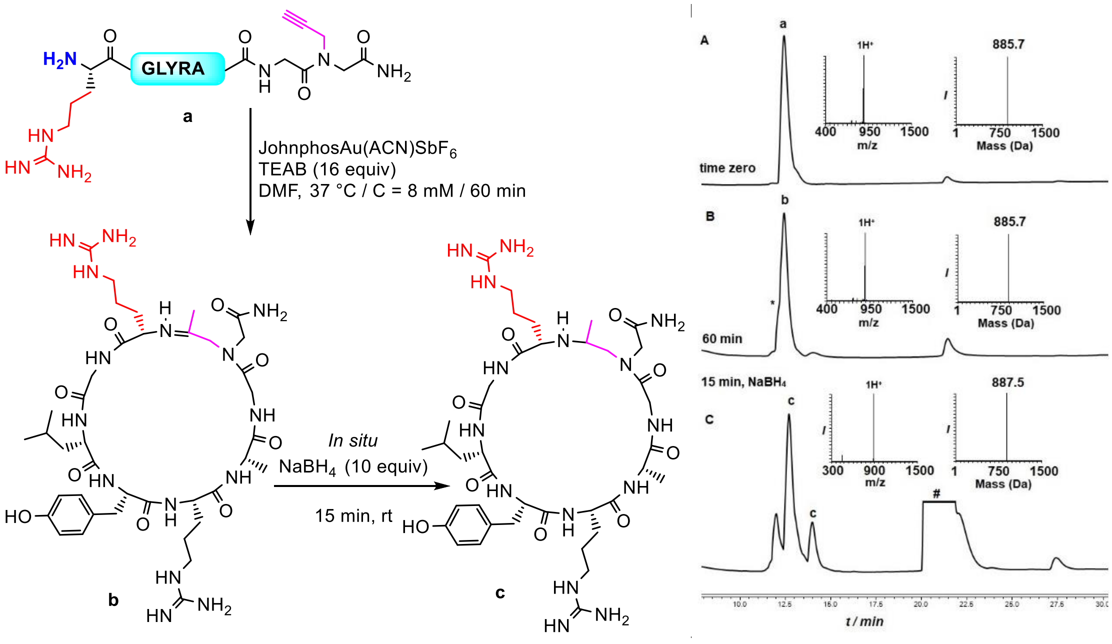

Figure S5: RGLYRAG(prop)G peptide reaction with JohnPhosAu(ACN)SbF_6: (A) Analytical HPLC and mass analysis of pure RGLYRAG(prop)G. Peak a corresponds to RGLYRAG(prop)G with the observed mass 885.7 ± 0.0 Da (calcd 886.0 Da) (r.t. = 12.29 min). (B) Analytical HPLC and mass analysis after 60 min incubation at 37 °C with JohnPhosAu(ACN)SbF6. Peak b corresponds to cyclization product with the observed mass 885.7 ± 0.0 Da (calcd 886.0 Da) (r.t. = 12.29 min). (C) Analytical HPLC and mass analysis after 15 min incubation at rt with $NaBH_4$. Peak c corresponds to the reduced cyclized products (diastereomers) with the observed mass 887.5 ± 0.0 Da (calcd 888.0 Da). (*) partial hydrolysis of the imine during column separation. (#) metal complex.

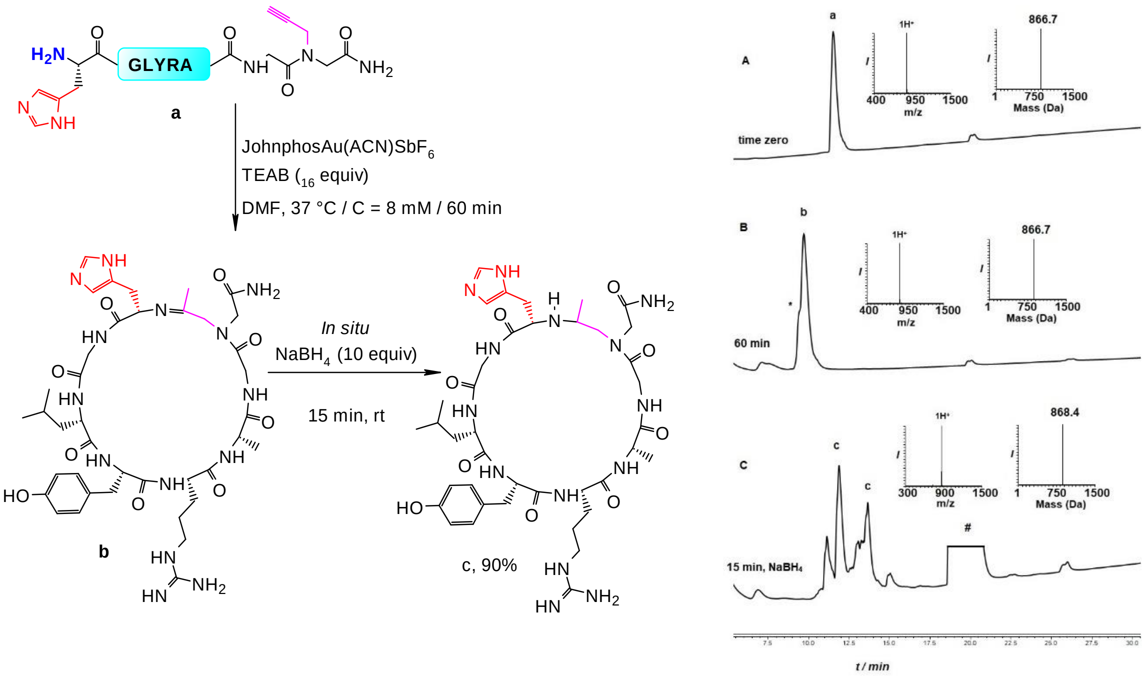

Figure S6: HGLYRAG(prop)G peptide reaction with JohnPhosAu(ACN)SbF_6: (A) Analytical HPLC and mass analysis of pure HGLYRAG(prop)G. Peak a corresponds to HGLYRAG(prop)G with the observed mass 866.7 ± 0.0 Da (calcd 866.4 Da) (r.t. = 11.59 min). (B) Analytical HPLC and mass analysis after 60 min incubation at 37 °C with JohnPhosAu(ACN)SbF6. Peak b corresponds to cyclization product with the observed mass 866.7 ± 0.0 Da (calcd 866.4 Da) (r.t. = 9.6 min). (C) Analytical HPLC and mass analysis after 15 min incubation at rt with $NaBH_4$. Peak c corresponds to the reduced cyclized products (diastereomers) with the observed mass 868.4 ± 0.0 Da (calcd 868.4 Da). (*) partial hydrolysis of the imine during column separation. (#) metal complex.

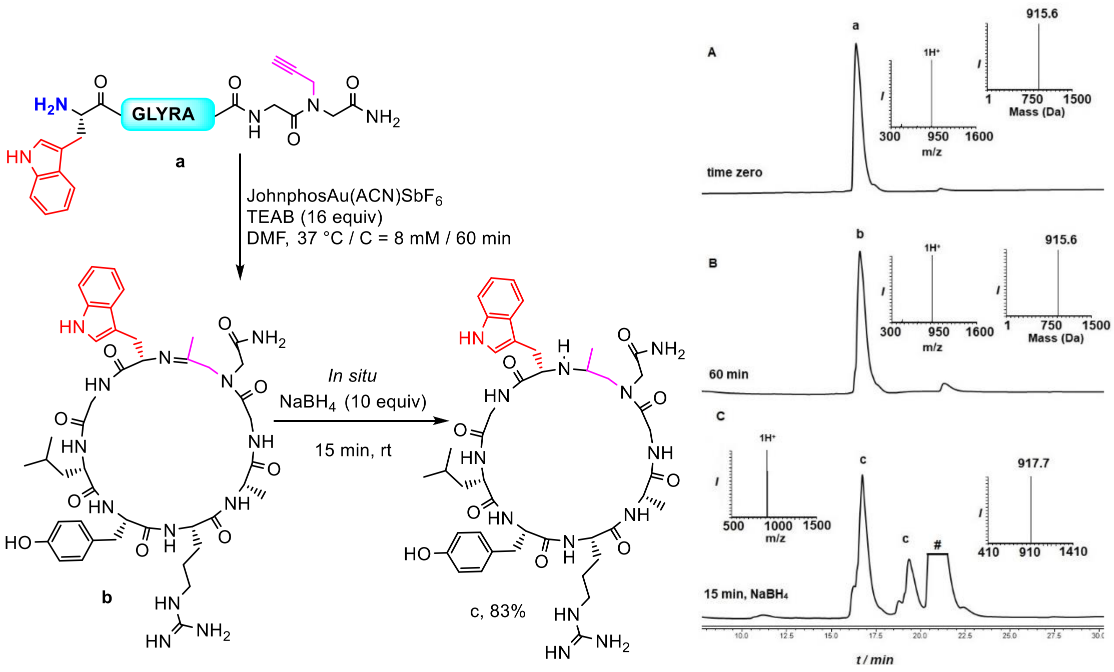

Figure S7: WGLYRAG(prop)G peptide reaction with JohnPhosAu(ACN)SbF_6: (A) Analytical HPLC and mass analysis of pure WGLYRAG(prop)G . Peak a corresponds to WGLYRAG(prop)G with the observed mass 915.6 ± 0.0 Da (calcd 916.0 Da) (r.t. = 16.60 min). (B) Analytical HPLC and mass analysis after 60 min incubation at 37 °C with JohnPhosAu(ACN)SbF6. Peak b corresponds to cyclization product with the observed mass 915.6 ± 0.0 Da (calcd 916.0 Da) (r.t. = 16.60 min). (C) Analytical HPLC and mass analysis after 15 min incubation at rt with $NaBH_4$. Peak c corresponds to the reduced cyclized products (diastereomers) with the observed mass 917.7 ± 0.0 Da (calcd 918.0 Da), HRMS (ESI+): $[M+H]^+$ calcd. 918.4950 and found 918.4938. (*) partial hydrolysis of the imine during column separation. (#) metal complex.

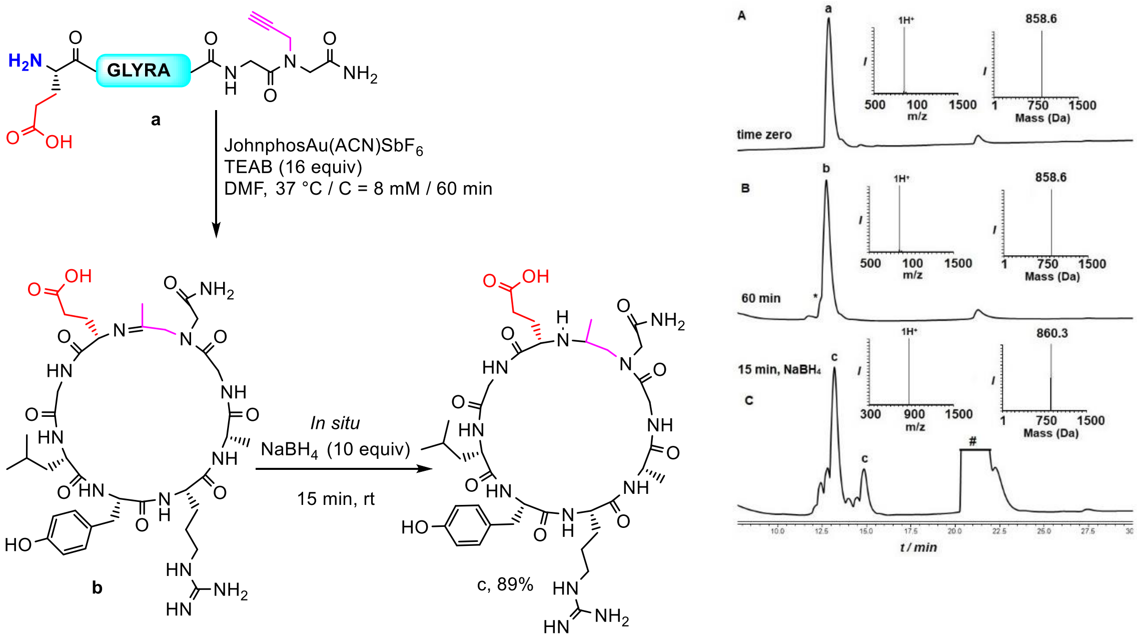

Figure S8: EGLYRAG(prop)G peptide reaction with JohnPhosAu(ACN)SbF_6: (A) Analytical HPLC and mass analysis of pure EGLYRAG(prop)G . Peak a corresponds to EGLYRAG(prop)G with the observed mass 858.6 ± 0.0 Da (calcd 858.9 Da) (r.t. = 12.87 min). (B) Analytical HPLC and mass analysis after 60 min incubation at 37 °C with JohnPhosAu(ACN)SbF6. Peak b corresponds to cyclization product with the observed mass 858.6 ± 0.0 Da (calcd 858.9 Da) (r.t. = 12.74 min). (C) Analytical HPLC and mass analysis after 15 min incubation at rt with $NaBH_4$. Peak c corresponds to the reduced cyclized products (diastereomers) with the observed mass 860.3 ± 0.0 Da (calcd 860.9 Da). (*) partial hydrolysis of the imine during column separation. (#) metal complex.

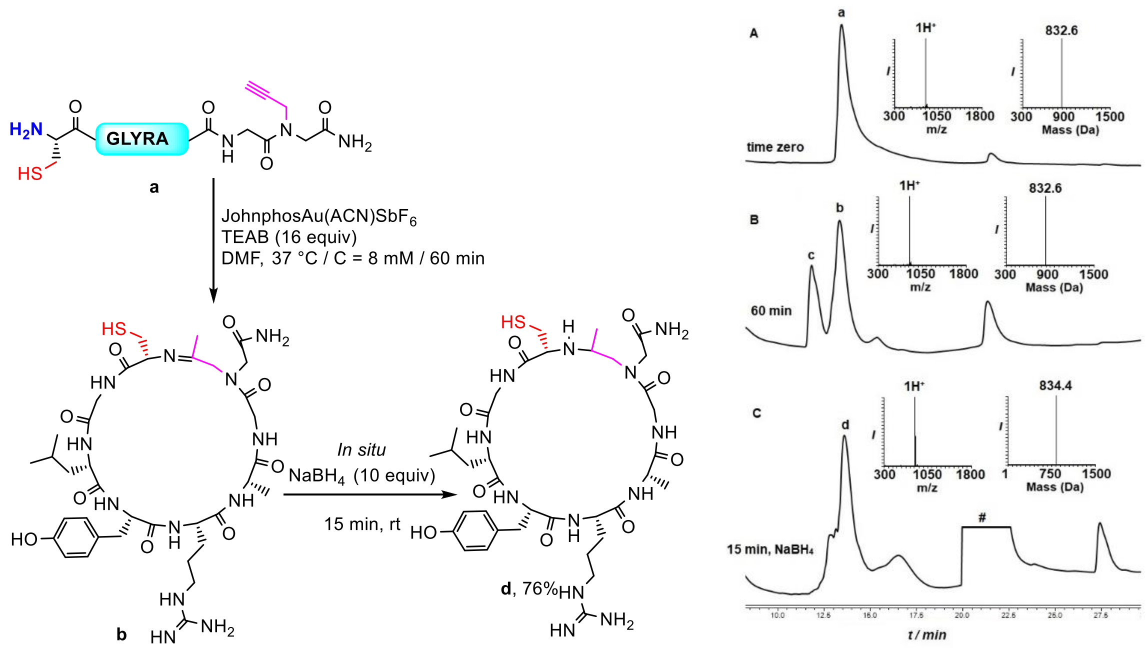

Figure S9: CGLYRAG(prop)G peptide reaction with JohnPhosAu(ACN)SbF_6: (A) Analytical HPLC and mass analysis of pure CGLYRAG(prop)G . Peak a corresponds to CGLYRAG(prop)G with the observed mass 832.6 ± 0.0 Da (calcd 832.9 Da) (r.t. = 13.36 min). (B) Analytical HPLC and mass analysis after 60 min incubation at 37 °C with JohnPhosAu(ACN)SbF6. Peak b corresponds to cyclization product with the observed mass 832.6 ± 0.0 Da (calcd 832.9 Da) (r.t. = 13.22 min). Peak c corresponds to partial hydrolysis of the imine during column separation. (C) Analytical HPLC and mass analysis after 15 min incubation at rt with $NaBH_4$. Peak d corresponds to the reduced cyclized products (diastereomers) with the observed mass 834.4 ± 0.0 Da (calcd 834.9 Da). (#) metal complex.

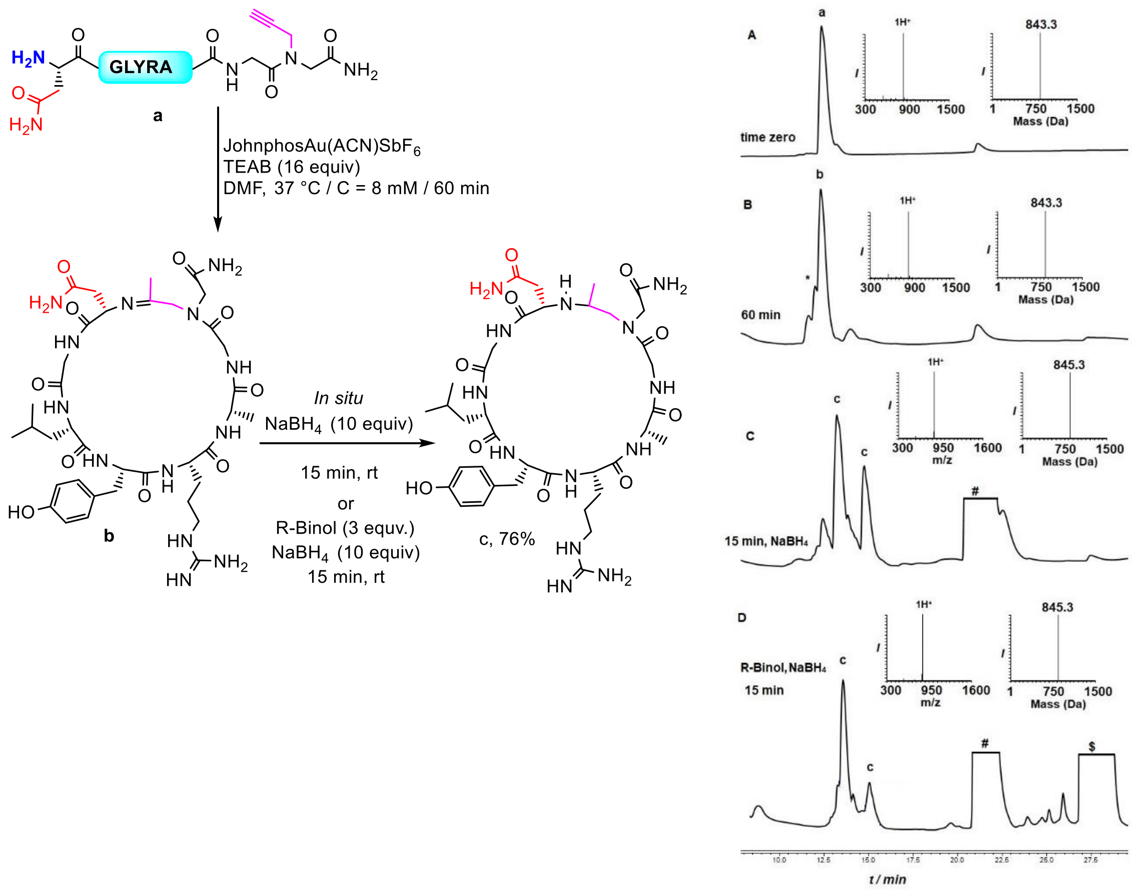

Figure S10: NGLYRAG(prop)G peptide reaction with JohnPhosAu(ACN)SbF_6: (A) Analytical HPLC and mass analysis of pure NGLYRAG(prop)G . Peak a corresponds to NGLYRAG(prop)G with the observed mass 843.3 ± 0.0 Da (calcd 843.9 Da) (r.t. = 12.43 min). (B) Analytical HPLC and mass analysis after 60 min incubation at 37 °C with JohnPhosAu(ACN)SbF6. Peak b corresponds to cyclization product with the observed mass 843.3 ± 0.0 Da (calcd 843.9 Da) (r.t. = 12.43 min). (C) Analytical HPLC and mass analysis after 15 min incubation at rt with $NaBH_4$. Peak c corresponds to the reduced cyclized products (diastereomers) with the observed mass 845.3 ± 0.0 Da (calcd 845.9 Da). (D) Analytical HPLC and mass analysis after 15 min incubation at rt with R-BINOL and $NaBH_4$. Peak c corresponds to the reduced cyclized products (diastereomers) with the observed mass 845.3 ± 0.0 Da (calcd 845.9 Da). (*) partial hydrolysis of the imine during column separation. (#) metal complex. ($) ligand related material.

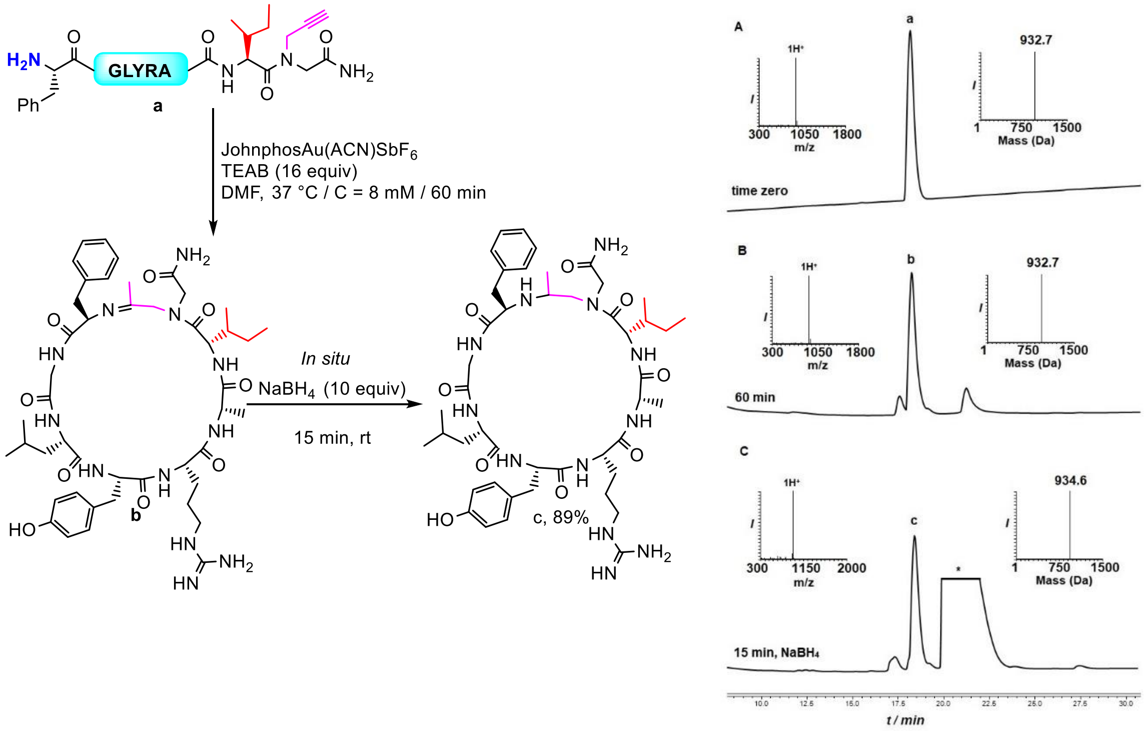

Figure S11: FGLYRAI(prop)G peptide reaction with JohnPhosAu(ACN)SbF_6: (A) Analytical HPLC and mass analysis of pure FGLYRAI(prop)G . Peak a corresponds to FGLYRAI(prop)G with the observed mass 932.7 ± 0.0 Da (calcd 933.0 Da) (r.t. = 18.30 min). (B) Analytical HPLC and mass analysis after 60 min incubation at 37 °C with JohnPhosAu(ACN)SbF6. Peak b corresponds to cyclization product with the observed mass 932.7 ± 0.0 Da (calcd 933.0 Da) (r.t. = 18.20 min). (C) Analytical HPLC and mass analysis after 15 min incubation at rt with $NaBH_4$. Peak c corresponds to the reduced cyclized products (diastereomers) with the observed mass 934.6 ± 0.0 Da (calcd 935.0 Da). (*) metal complex.

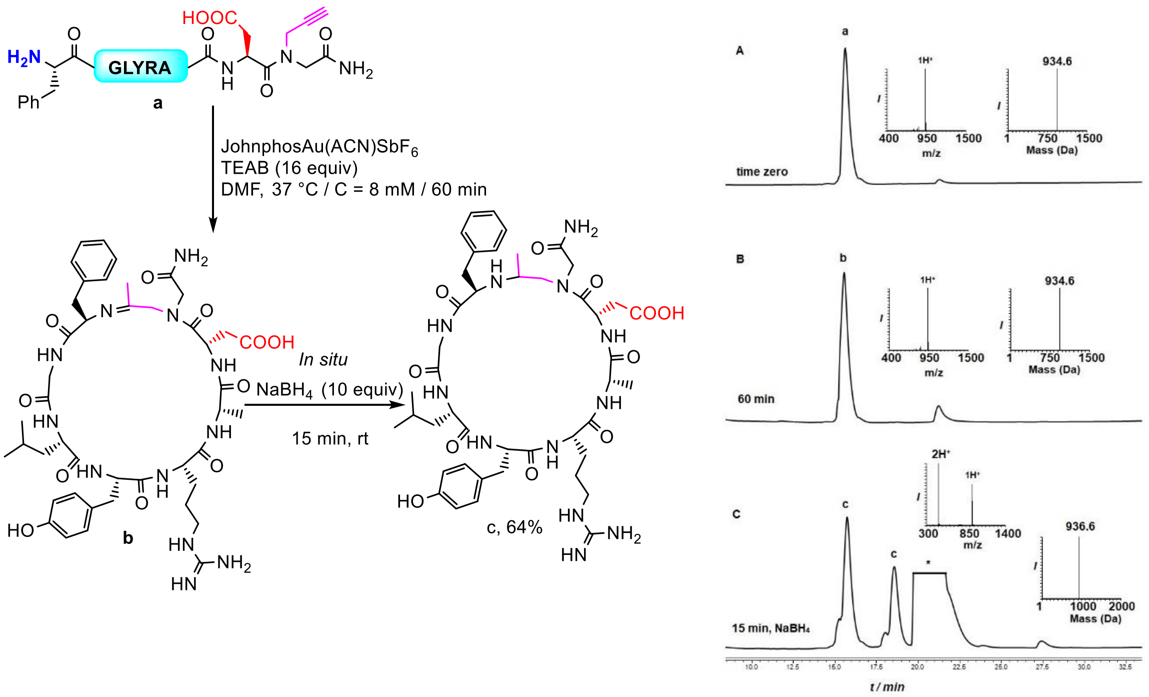

Figure S12: FGLYRAD(prop)G peptide reaction with JohnPhosAu(ACN)SbF_6: (A) Analytical HPLC and mass analysis of pure FGLYRAD(prop)G . Peak a corresponds to FGLYRAD(prop)G with the observed mass 934.6 ± 0.0 Da (935.0 Da) (r.t. = 15.60 min). (B) Analytical HPLC and mass analysis after 60 min incubation at 37 °C with JohnPhosAu(ACN)SbF6. Peak b corresponds to cyclization product with the observed mass 934.6 ± 0.0 Da (calcd 935.0 Da) (r.t. = 15.60 min). (C) Analytical HPLC and mass analysis after 15 min incubation at rt with $NaBH_4$. Peak c corresponds to the reduced cyclized products (diastereomers) with the observed mass 936.6 ± 0.0 Da (calcd 937.0 Da). (*) metal complex.

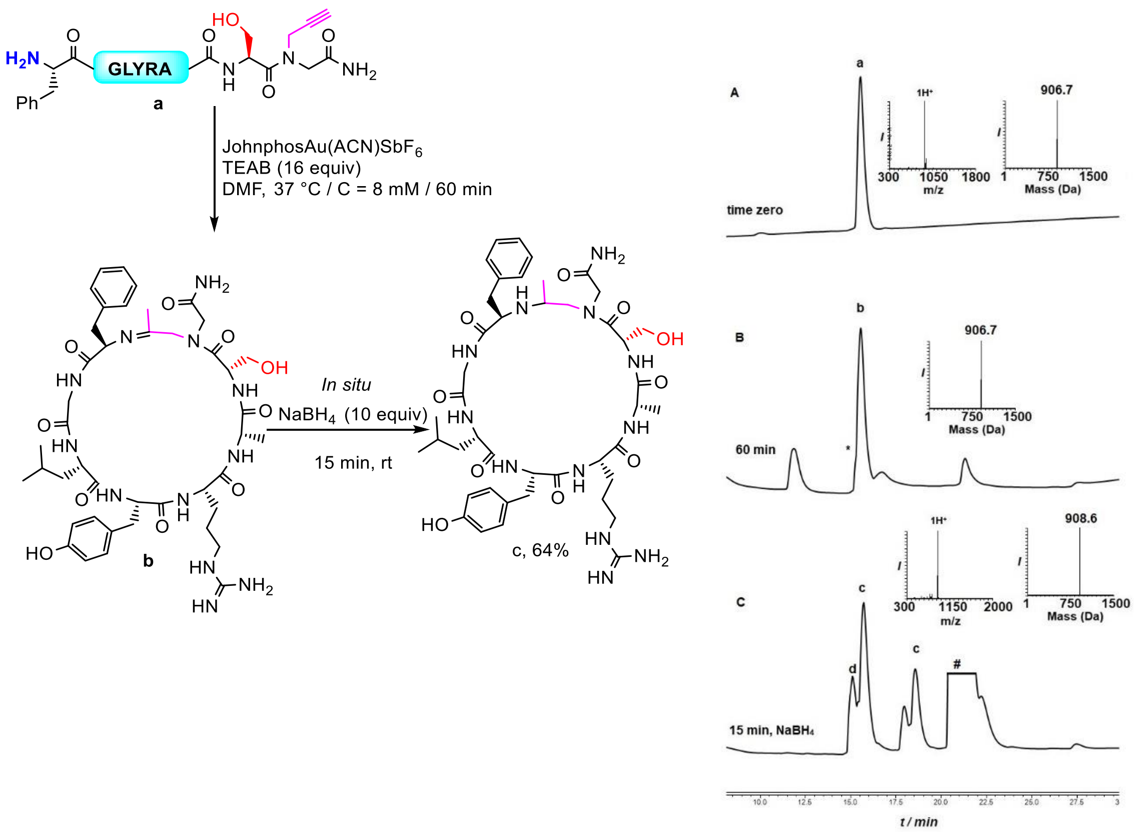

Figure S13: FGLYRAS(prop)G peptide reaction with JohnPhosAu(ACN)SbF_6: (A) Analytical HPLC and mass analysis of pure FGLYRAS(prop)G . Peak a corresponds to FGLYRAS(prop)G with the observed mass 906.7 ± 0.0 Da (calcd 907.0 Da) (r.t. = 15.50 min). (B) Analytical HPLC and mass analysis after 60 min incubation at 37 °C with JohnPhosAu(ACN)SbF6. Peak b corresponds to cyclization product with the observed mass 906.7 ± 0.0 Da (calcd 907.0 Da) (r.t. = 15.50 min). (C) Analytical HPLC and mass analysis after 15 min incubation at rt with $NaBH_4$. Peak c corresponds to the reduced cyclized products (diastereomers) with the observed mass 908.6 ± 0.0 Da (calcd 909.0 Da). Peak d corresponds to the reduced water addition product. (*) mixture of partial hydrolysis of the imine during column separation and water addition byproduct. (#) metal complex.

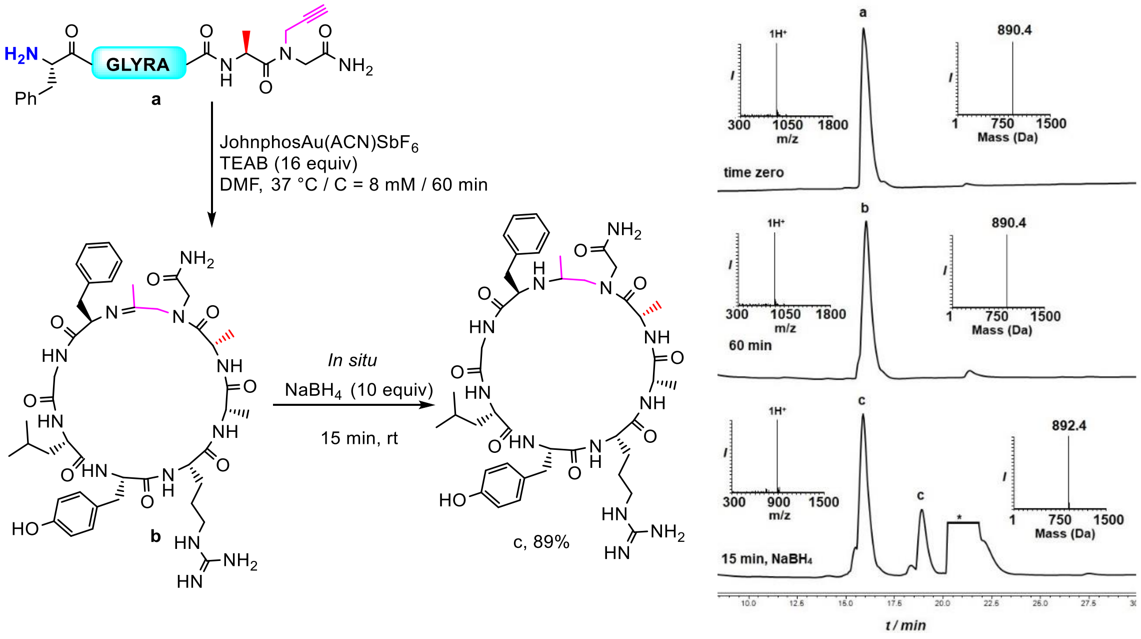

Figure S14: FGLYRAA(prop)G peptide reaction with JohnPhosAu(ACN)SbF_6: (A) Analytical HPLC and mass analysis of pure FGLYRAA(prop)G . Peak a corresponds to FGLYRAA(prop)G with the observed mass 890.4 ± 0.0 Da (calcd 891.0 Da) (r.t. = 15.90 min). (B) Analytical HPLC and mass analysis after 60 min incubation at 37 °C with JohnPhosAu(ACN)SbF6. Peak b corresponds to cyclization product with the observed mass 890.4 ± 0.0 Da (calcd 891.0 Da) (r.t. = 15.90 min). (C) Analytical HPLC and mass analysis after 15 min incubation at rt with $NaBH_4$. Peak c corresponds to the reduced cyclized products (diastereomers) with the observed mass 892.4 ± 0.0 Da (calcd 893.0 Da). (*) metal complex.

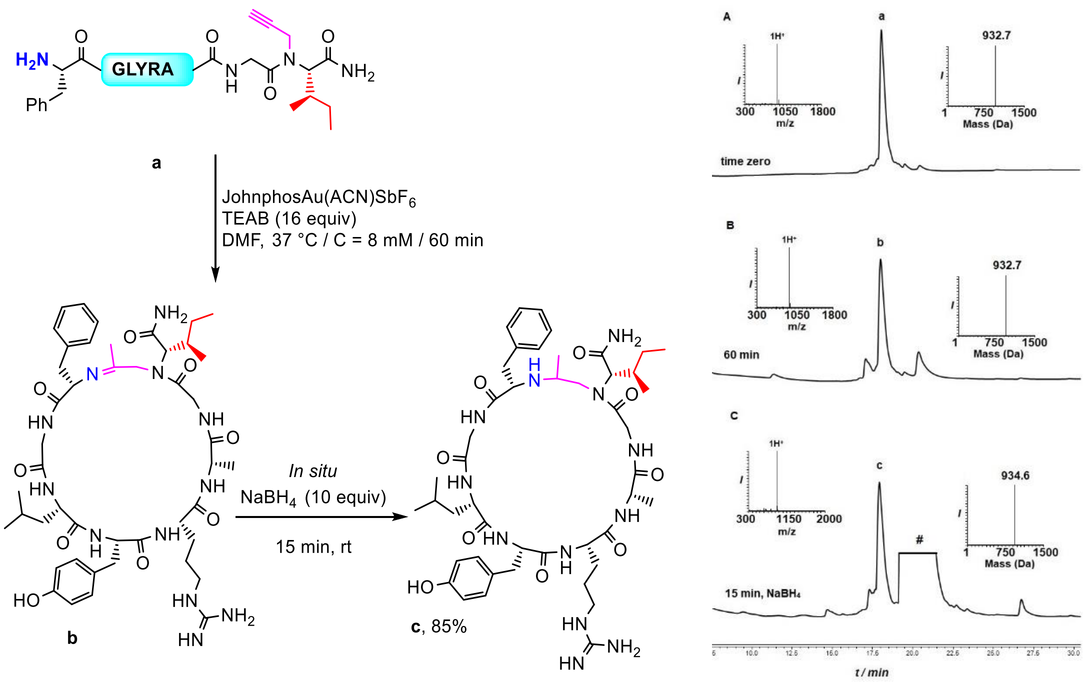

Figure S15: FGLYRAG(prop)I peptide reaction with JohnPhosAu(ACN)SbF_6: (A) Analytical HPLC and mass analysis of pure FGLYRAG(prop)I. Peak a corresponds to FGLYRAG(prop)I with the observed mass 932.7 ± 0.0 Da (calcd 933.0 Da) (r.t. = 18.10 min). (B) Analytical HPLC and mass analysis after 60 min incubation at 37 °C with JohnPhosAu(ACN)SbF6. Peak b corresponds to cyclization product with the observed mass 932.7 ± 0.0 Da (calcd 933.0 Da) (r.t. = 18.10 min). (C) Analytical HPLC and mass analysis after 15 min incubation at rt with $NaBH_4$. Peak c corresponds to the reduced cyclized products (diastereomers) with the observed mass 934.6 ± 0.0 Da (calcd 935.0 Da), HRMS (ESI+): $[M+H]^+$ calcd. 935.5460 and found 935.5450. (#) metal complex.

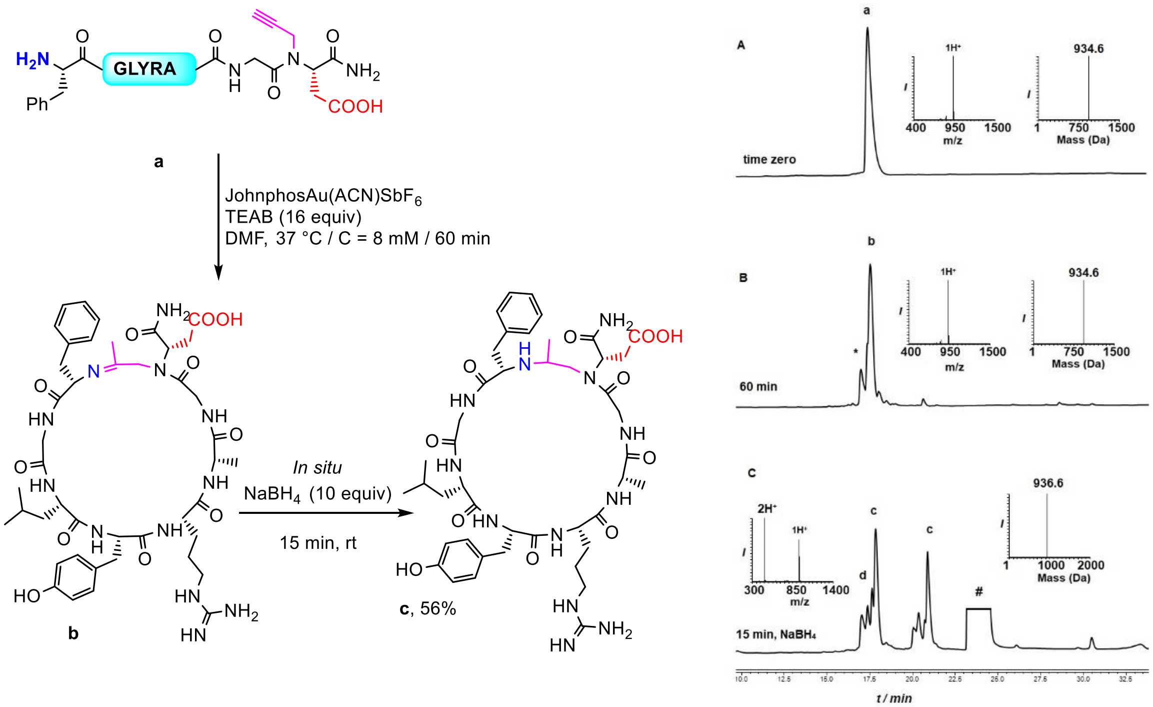

Figure S16: FGLYRAG(prop)D peptide reaction with JohnPhosAu(ACN)SbF_6: (A) Analytical HPLC and mass analysis of pure FGLYRAG(prop)D. Peak a corresponds to FGLYRAG(prop)D with the observed mass 934.6 ± 0.0 Da (calcd 935.0 Da) (r.t. = 17.52 min). (B) Analytical HPLC and mass analysis after 60 min incubation at 37 °C with JohnPhosAu(ACN)SbF_6. Peak b corresponds to cyclization product with the observed mass 934.6 ± 0.0 Da (calcd 935.0 Da) (r.t. = 17.52 min). (C) Analytical HPLC and mass analysis after 15 min incubation at rt with $NaBH_4$. Peak c corresponds to the reduced cyclized products (diastereomers) with the observed mass 936.6 ± 0.0 Da (calcd 937.0 Da). Peak d corresponds to the reduced water addition product. (*) mixture of partial hydrolysis of the imine during column separation and water addition byproduct. (#) metal complex.

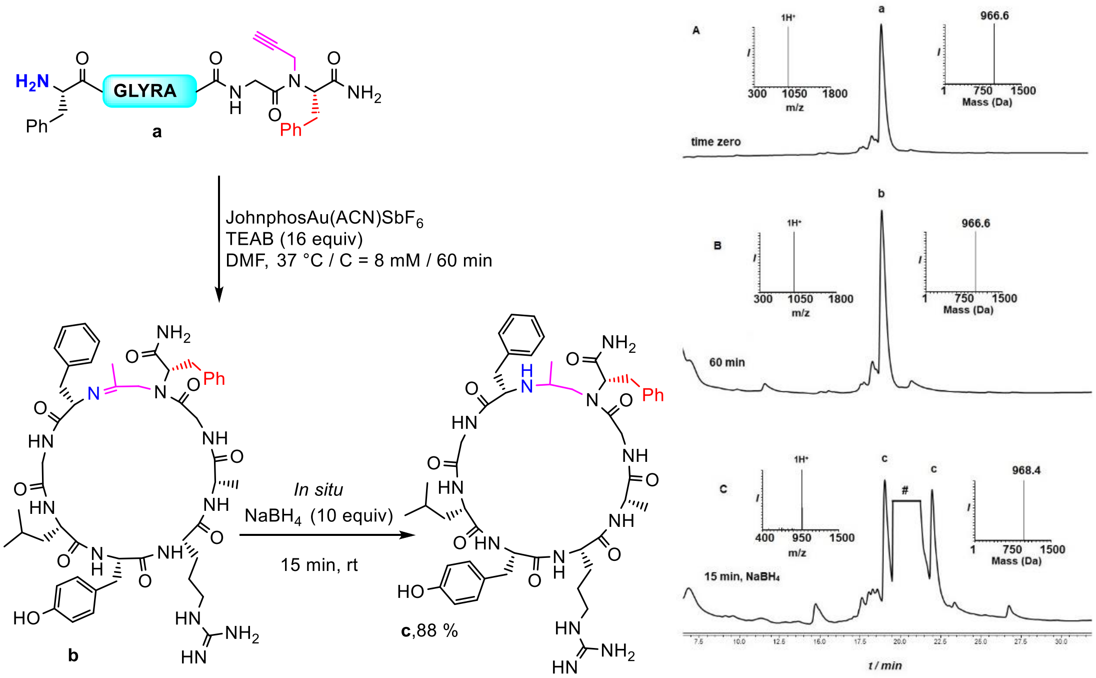

Figure S17: FGLYRAG(prop)F peptide reaction with JohnPhosAu(ACN)SbF_6: (A) Analytical HPLC and mass analysis of pure FGLYRAG(prop)F. Peak a corresponds to FGLYRAG(prop)F with the observed mass 966.6 ± 0.0 Da (calcd 967.0 Da) (r.t. = 18.70 min). (B) Analytical HPLC and mass analysis after 60 min incubation at 37 °C with JohnPhosAu(ACN)SbF_6. Peak b corresponds to cyclization product with the observed mass 966.6 ± 0.0 Da (calcd 967.0 Da) (r.t. = 18.70 min). (C) Analytical HPLC and mass analysis after 15 min incubation at rt with $NaBH_4$. Peak c corresponds to the reduced cyclized products (diastereomers) with the observed mass 968.4 ± 0.0 Da (calcd 969.0 Da). (#) metal complex.

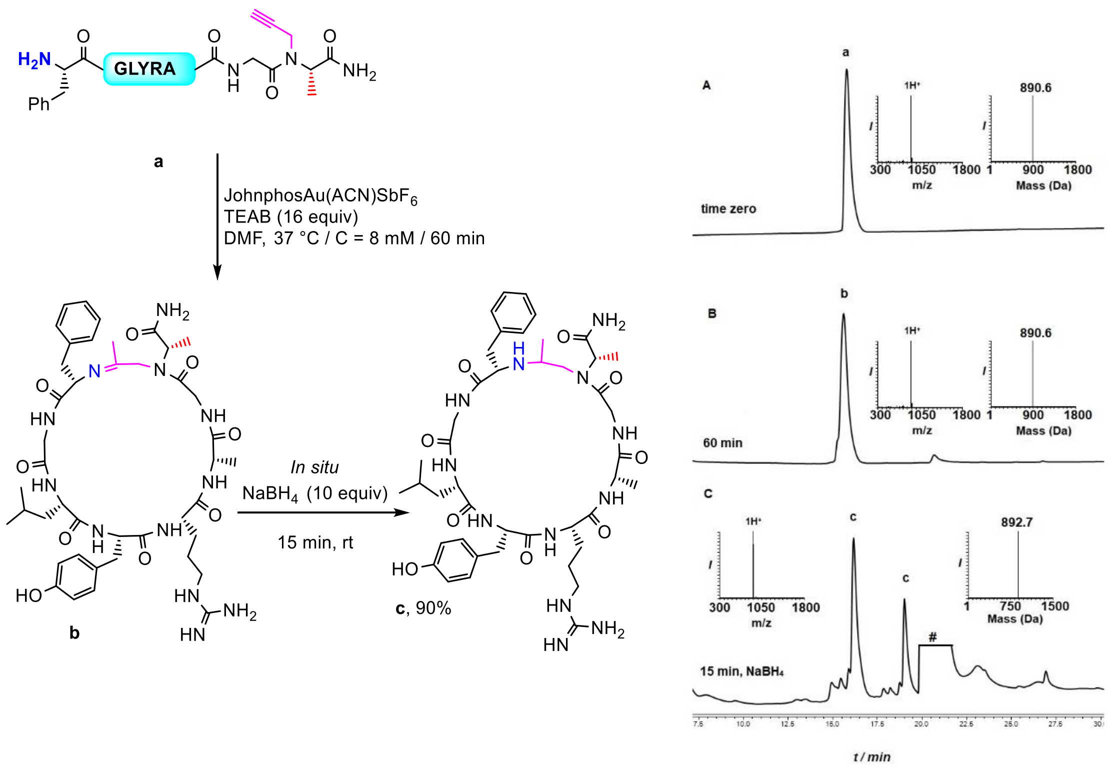

Figure S18: FGLYRAG(prop)A peptide reaction with JohnPhosAu(ACN)SbF_6: (A) Analytical HPLC and mass analysis of pure FGLYRAG(prop)A. Peak a corresponds to FGLYRAG(prop)A with the observed mass 890.6 ± 0.0 Da (calcd 890.9 Da) (r.t. = 15.78 min). (B) Analytical HPLC and mass analysis after 60 min incubation at 37 °C with JohnPhosAu(ACN)SbF_6. Peak b corresponds to cyclization product with the observed mass 890.6 ± 0.0 Da (calcd 890.9 Da) (r.t. = 15.75 min). (C) Analytical HPLC and mass analysis after 15 min incubation at rt with $NaBH_4$. Peak c corresponds to the reduced cyclized products (diastereomers) with the observed mass 892.7 ± 0.0 Da (calcd 892.9 Da). (#) metal complex.

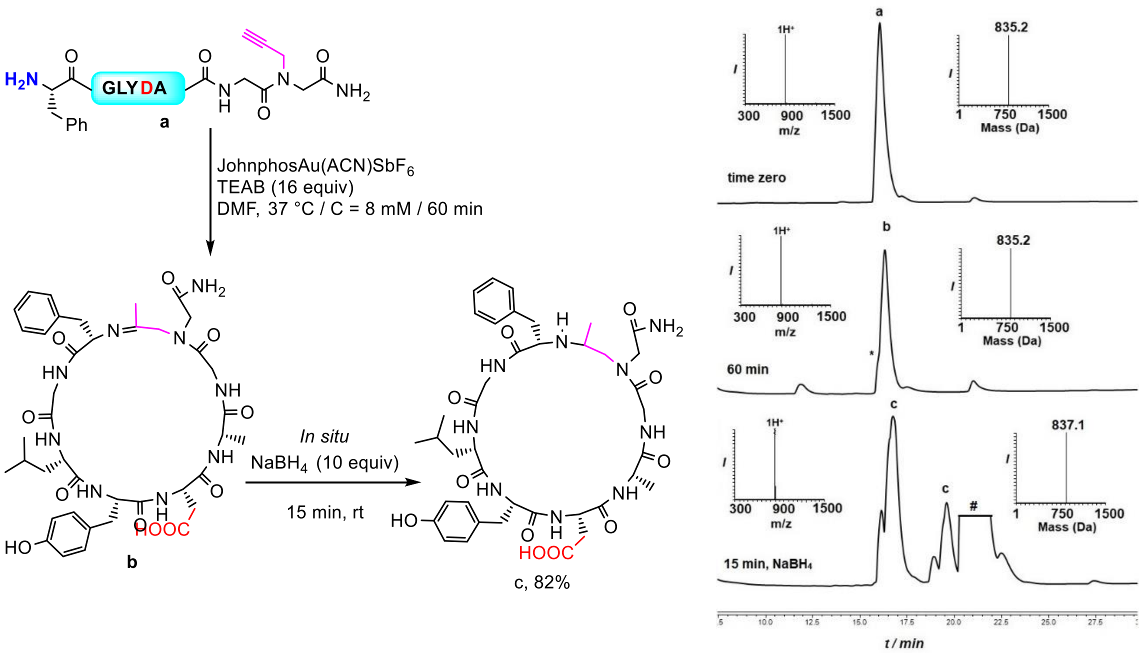

Figure S19: FGLYDAG(prop)G peptide reaction with JohnPhosAu(ACN)SbF_6: (A) Analytical HPLC and mass analysis of pure FGLYDAG(prop)G. Peak a corresponds to FGLYDAG(prop)G with the observed mass 835.2 ± 0.0 Da (calcd 835.9 Da) (r.t. = 16.12 min). (B) Analytical HPLC and mass analysis after 60 min incubation at 37 °C with JohnPhosAu(ACN)SbF_6. Peak b corresponds to cyclization product with the observed mass 835.2 ± 0.0 Da (calcd 835.9 Da) (r.t. = 16.38 min). (C) Analytical HPLC and mass analysis after 15 min incubation at rt with $NaBH_4$. Peak c corresponds to the reduced cyclized products (diastereomers) with the observed mass 837.1 ± 0.0 Da (calcd 837.9 Da). (*) partial hydrolysis of the imine during column separation. (#) metal complex.

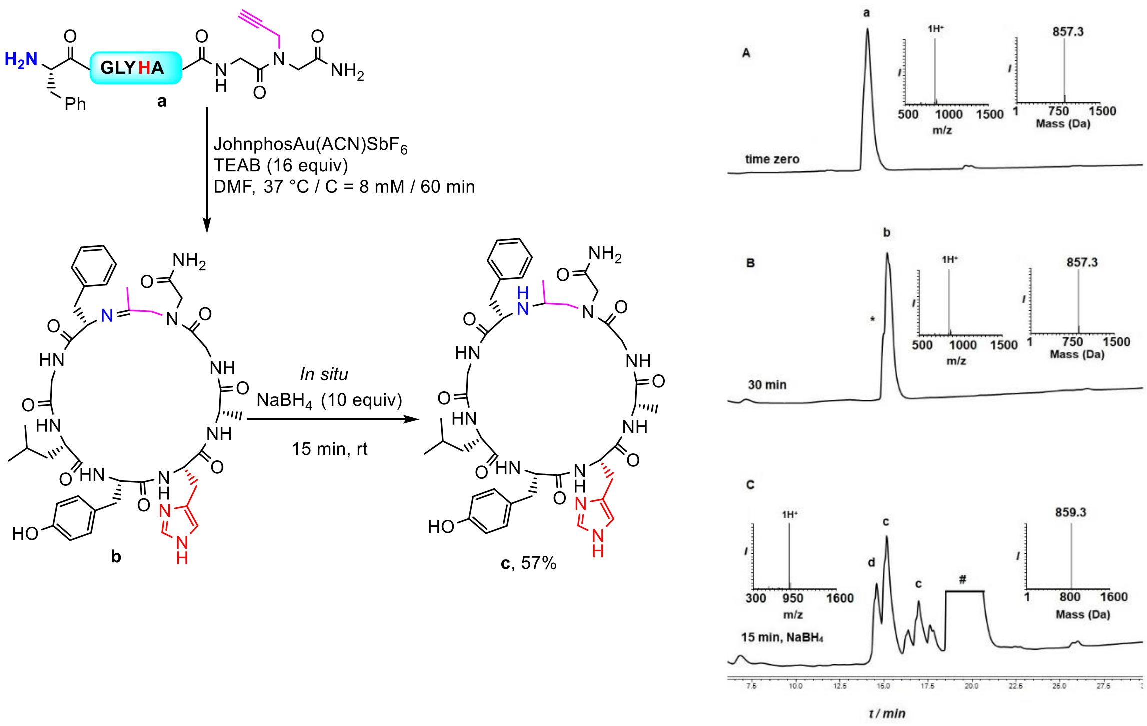

Figure S20: FGLYHAG(prop)G peptide reaction with JohnPhosAu(ACN)SbF_6: (A) Analytical HPLC and mass analysis of pure FGLYHAG(prop)G. Peak a corresponds to FGLYHAG(prop)G with the observed mass 857.3 ± 0.0 Da (calcd 857.9 Da) (r.t. = 13.90 min). (B) Analytical HPLC and mass analysis after 60 min incubation at 37 °C with JohnPhosAu(ACN)SbF_6. Peak b corresponds to cyclization product with the observed mass 857.3 ± 0.0 Da (calcd 857.9 Da) (r.t. = 14.90 min). (C) Analytical HPLC and mass analysis after 15 min incubation at rt with $NaBH_4$. Peak c corresponds to the reduced cyclized products (diastereomers) with the observed mass 859.3 ± 0.0 Da (calcd 859.9 Da). Peak d corresponds to the reduced water addition product. (*) mixture of partial hydrolysis of the imine during column separation and water addition byproduct. (#) metal complex.

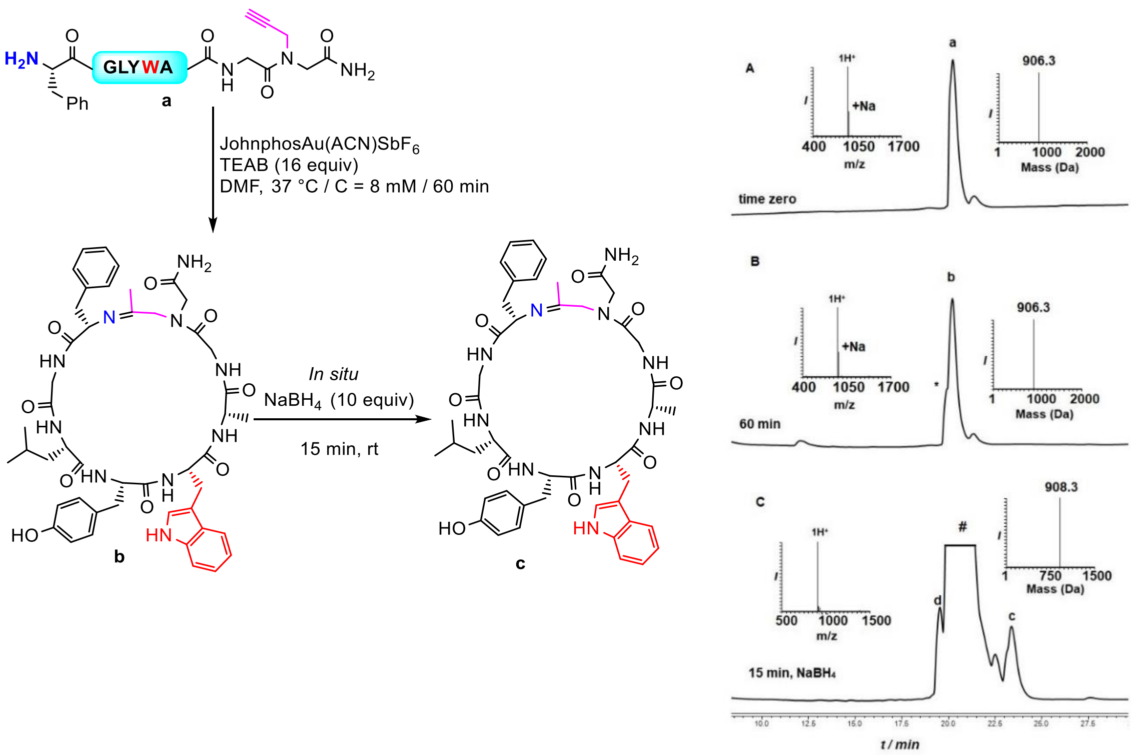

Figure S21: FGLYWAG(prop)G peptide reaction with JohnPhosAu(ACN)SbF_6: (A) Analytical HPLC and mass analysis of pure FGLYWAG(prop)G. Peak a corresponds to FGLYWAG(prop)G with the observed mass 906.3 ± 0.0 Da (calcd 906.9 Da) (r.t. = 20.20 min). (B) Analytical HPLC and mass analysis after 60 min incubation at 37 °C with JohnPhosAu(ACN)SbF_6. Peak b corresponds to cyclization product with the observed mass 906.3 ± 0.0 Da (calcd 906.9 Da) (r.t. = 20.10 min). (C) Analytical HPLC and mass analysis after 15 min incubation at rt with $NaBH_4$. Peak c corresponds to the reduced cyclized products (diastereomers) with the observed mass 908.3 ± 0.0 Da (calcd 908.9 Da) (major isomer is merged in the metal complex peak). Peak d corresponds to the reduced water addition product. (*) mixture of partial hydrolysis of the imine during column separation and water addition byproduct. (#) metal complex.

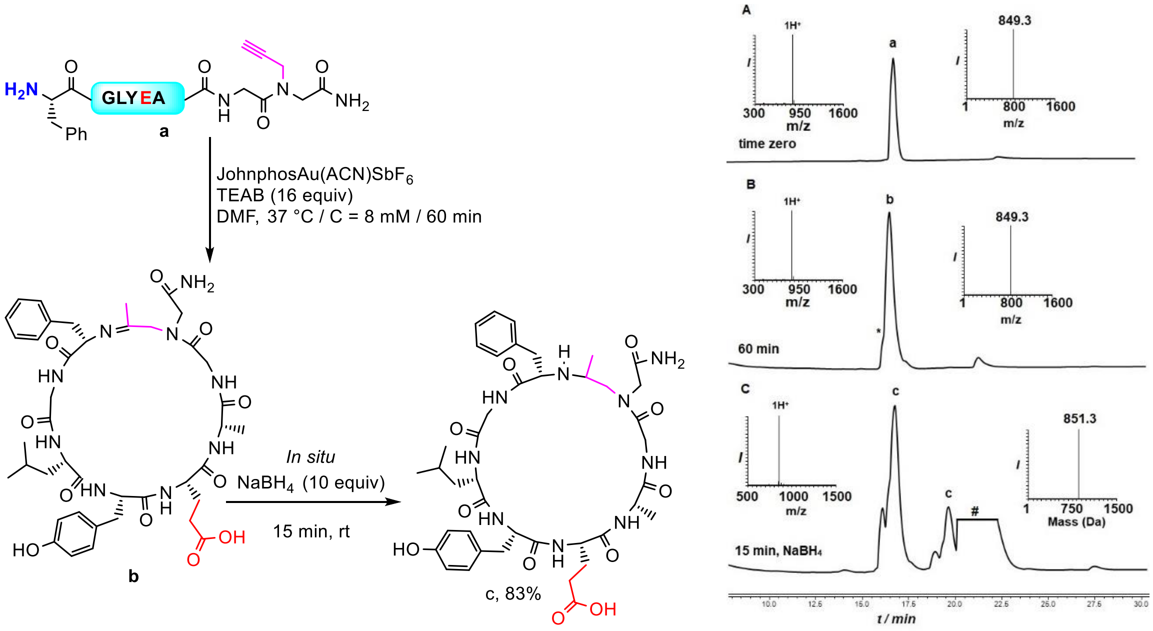

Figure S22: FGLYEAG(prop)G peptide reaction with JohnPhosAu(ACN)SbF_6: (A) Analytical HPLC and mass analysis of pure FGLYEAG(prop)G. Peak a corresponds to FGLYEAG(prop)G with the observed mass 849.3 ± 0.0 Da (calcd 849.9 Da) (r.t. = 16.60 min). (B) Analytical HPLC and mass analysis after 60 min incubation at 37 °C with JohnPhosAu(ACN)SbF_6. Peak b corresponds to cyclization product with the observed mass 849.3 ± 0.0 Da (calcd 849.9 Da) (r.t. = 16.50 min). (C) Analytical HPLC and mass analysis after 15 min incubation at rt with $NaBH_4$. Peak c corresponds to the reduced cyclized products (diastereomers) with the observed mass 851.3 ± 0.0 Da (calcd 851.9 Da). (*) partial hydrolysis of the imine during column separation. (#) metal complex.

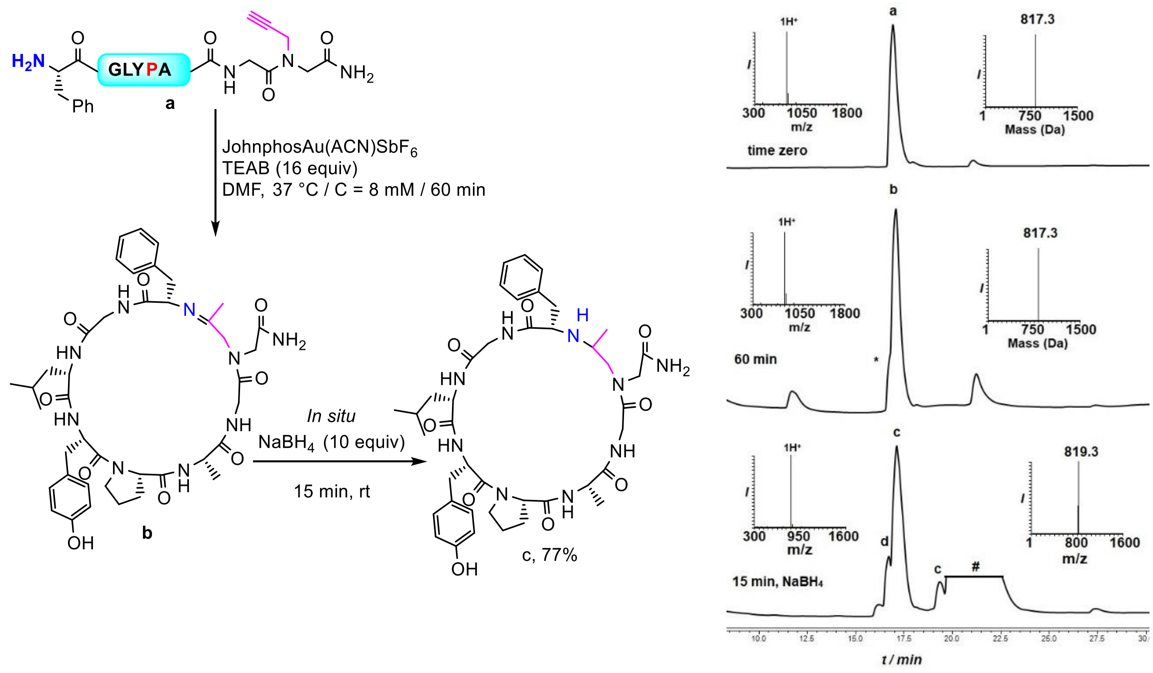

Figure S23: FGLYPAG(prop)G peptide reaction with JohnPhosAu(ACN)SbF_6: (A) Analytical HPLC and mass analysis of pure FGLYPAG(prop)G. Peak a corresponds to FGLYPAG(prop)G with the observed mass 817.3 ± 0.0 Da (calcd 817.9 Da) (r.t. = 17.10 min). (B) Analytical HPLC and mass analysis after 60 min incubation at 37 °C with JohnPhosAu(ACN)SbF_6. Peak b corresponds to cyclization product with the observed mass 817.3 ± 0.0 Da (calcd 817.9 Da) (r.t. = 17.10 min). (C) Analytical HPLC and mass analysis after 15 min incubation at rt with $NaBH_4$. Peak c corresponds to the reduced cyclized products (diastereomers) with the observed mass 819.3 ± 0.0 Da (calcd 819.9 Da). Peak d corresponds to the reduced water addition by-product. (*) mixture of partial hydrolysis of the imine during column separation and water addition byproduct. (#) metal complex.

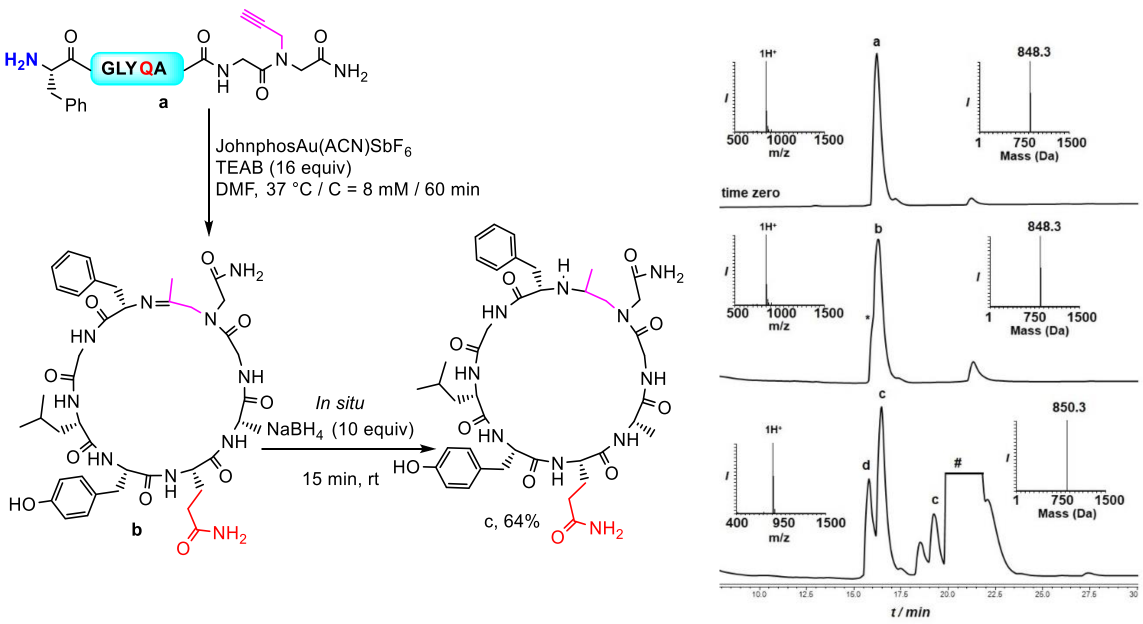

Figure S24: FGLYQAG(prop)G peptide reaction with JohnPhosAu(ACN)SbF_6: (A) Analytical HPLC and mass analysis of pure FGLYQAG(prop)G. Peak a corresponds to FGLYQAG(prop)G with the observed mass 848.3 ± 0.0 Da (calcd 848.9 Da) (r.t. = 16.20 min). (B) Analytical HPLC and mass analysis after 60 min incubation at 37 °C with JohnPhosAu(ACN)SbF_6. Peak b corresponds to cyclization product with the observed mass 848.3 ± 0.0 Da (calcd 848.9 Da) (r.t. = 16.20 min). (C) Analytical HPLC and mass analysis after 15 min incubation at rt with $NaBH_4$. Peak c corresponds to the reduced cyclized products (diastereomers) with the observed mass 850.3 ± 0.0 Da (calcd 850.9 Da). Peak d corresponds to the reduced water addition by-product. (*) mixture of partial hydrolysis of the imine during column separation and water addition byproduct. (#) metal complex.

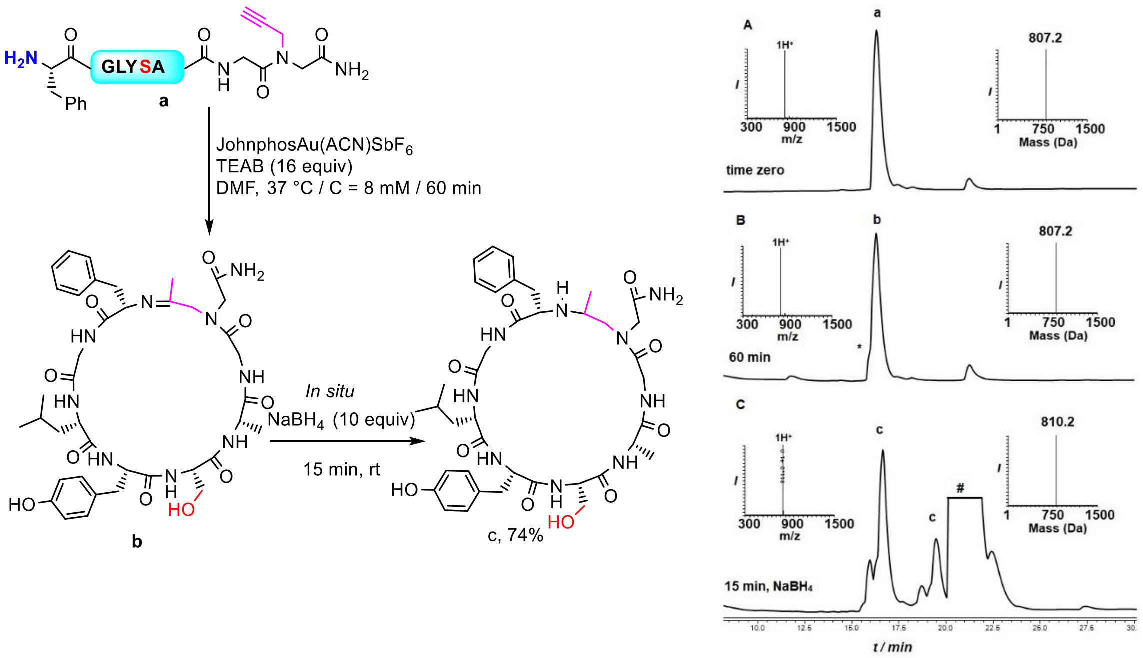

Figure S25: FGLYSAG(prop)G peptide reaction with JohnPhosAu(ACN)SbF_6: (A) Analytical HPLC and mass analysis of pure FGLYSAG(prop)G. Peak a corresponds to FGLYSAG(prop)G with the observed mass 807.2 ± 0.0 Da (calcd 807.8 Da) (r.t. = 16.30 min). (B) Analytical HPLC and mass analysis after 60 min incubation at 37 °C with JohnPhosAu(ACN)SbF_6. Peak b corresponds to cyclization product with the observed mass 807.2 ± 0.0 Da (calcd 807.8 Da) (r.t. = 16.30 min). (C) Analytical HPLC and mass analysis after 15 min incubation at rt with NaBH_4. Peak c corresponds to the reduced cyclized products (diastereomers) with the observed mass 810.2 ± 0.0 Da (calcd 809.8 Da), HRMS (ESI+): $[M+H]^+$ calcd. 810.4150 and found 810.4070. (*) partial hydrolysis of the imine during column separation. (#) metal complex.

Au(I)-Mediated cyclization of different chain lengths

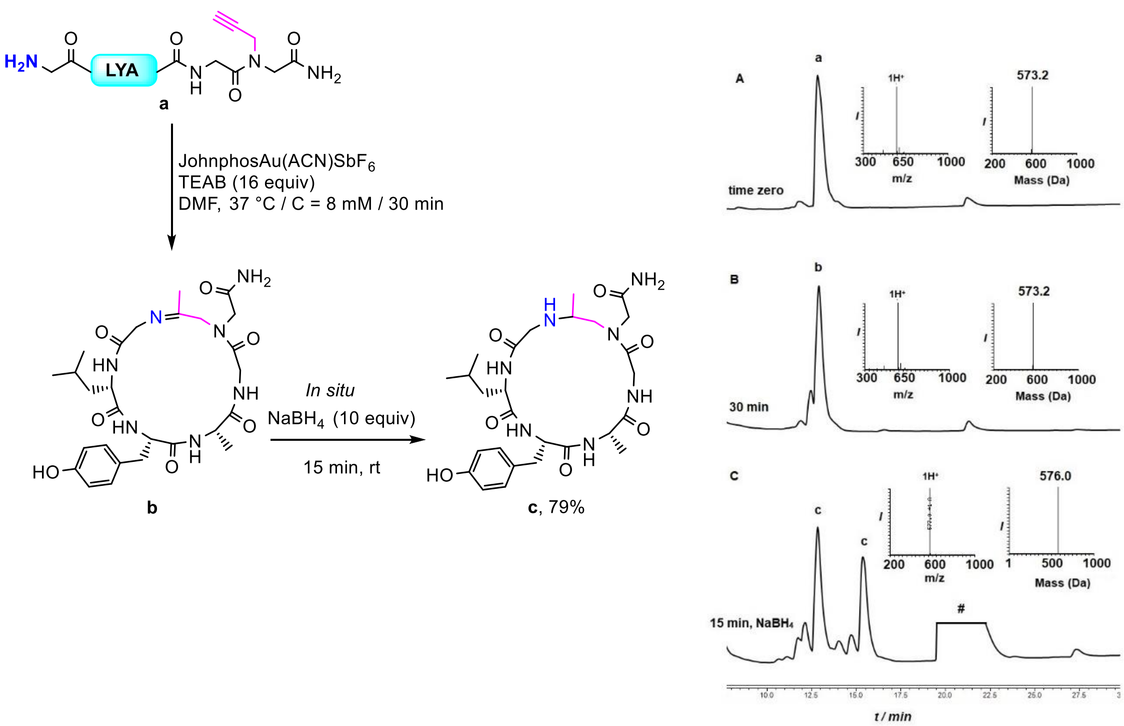

Figure S26: GLYAG(prop)G peptide reaction with JohnPhosAu(ACN)SbF_6: (A) Analytical HPLC and mass analysis of pure GLYAG(prop)G. Peak a corresponds to GLYAG(prop)G with the observed mass 573.2 ± 0.0 Da (calcd 573.6 Da) (r.t. = 12.90 min). (B) Analytical HPLC and mass analysis after 30 min incubation at 37 °C with JohnPhosAu(ACN)SbF_6. Peak b corresponds to cyclization product with the observed mass 573.2 ± 0.0 Da (calcd 573.6 Da) (r.t. = 12.90 min). (C) Analytical HPLC and mass analysis after 15 min incubation at rt with $NaBH_4$. Peak c corresponds to the reduced cyclized products (diastereomers) with the observed mass 576.0 ± 0.0 Da (calcd 575.6 Da). (#) metal complex.

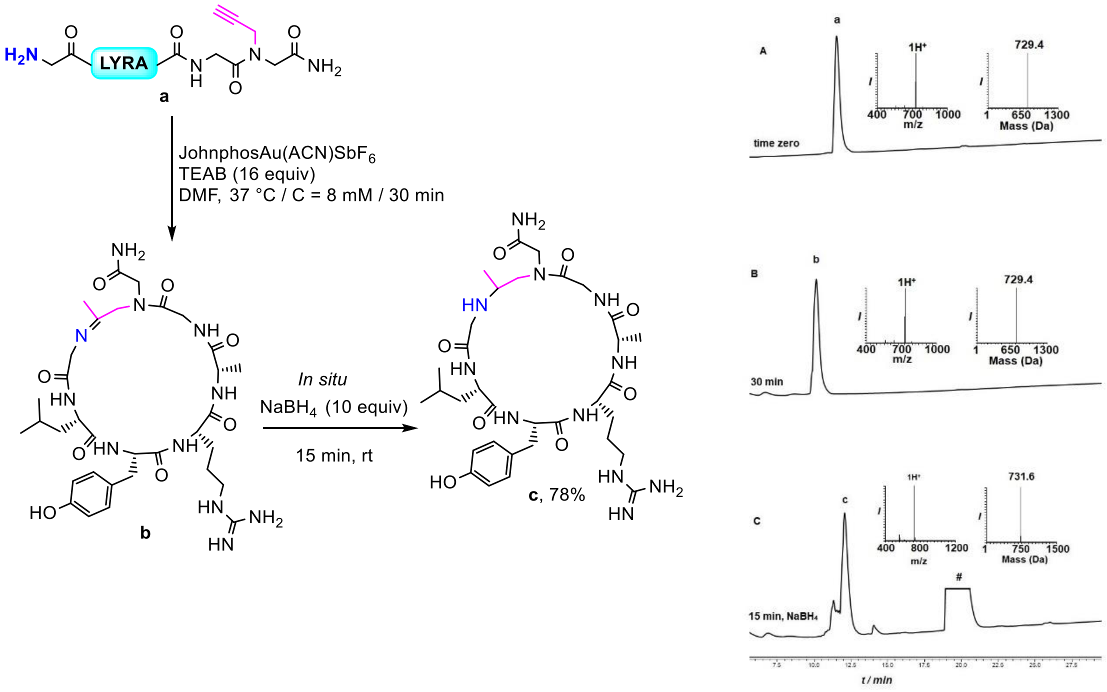

Figure S27: GLYRAG(prop)G peptide reaction with JohnPhosAu(ACN)SbF_6: (A) Analytical HPLC and mass analysis of pure GLYRAG(prop)G. Peak a corresponds to GLYRAG(prop)G with the observed mass 729.4 ± 0.0 Da (calcd 729.7 Da) (r.t. = 11.70 min). (B) Analytical HPLC and mass analysis after 30 min incubation at 37 °C with JohnPhosAu(ACN)SbF_6. Peak b corresponds to cyclization product with the observed mass 729.4 ± 0.0 Da (calcd 729.7 Da) (r.t. = 10.40 min). (C) Analytical HPLC and mass analysis after 15 min incubation at rt with $NaBH_4$. Peak c corresponds to the reduced cyclized products (diastereomers) with the observed mass 731.6 ± 0.0 Da (calcd 731.7 Da). (#) metal complex.

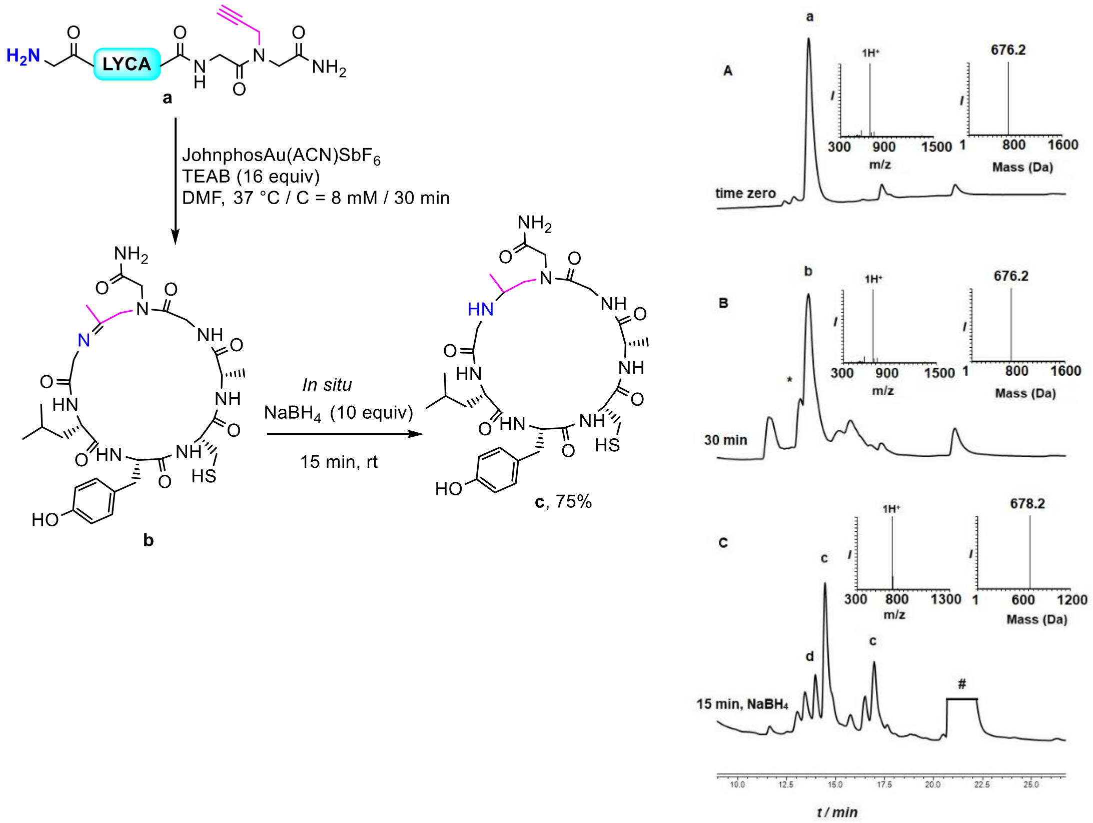

Figure S28: GLYCAG(prop)G peptide reaction with JohnPhosAu(ACN)SbF_6: (A) Analytical HPLC and mass analysis of pure FGLYCAG(prop)G. Peak a corresponds to FGLYCAG(prop)G with the observed mass 676.2 ± 0.0 Da (calcd 676.7 Da) (r.t. = 13.80 min). (B) Analytical HPLC and mass analysis after 30 min incubation at 37 °C with JohnPhosAu(ACN)SbF_6. Peak b corresponds to cyclization product with the observed mass 676.2 ± 0.0 Da (calcd 676.7 Da) (r.t. = 13.80 min). (C) Analytical HPLC and mass analysis after 15 min incubation at rt with $NaBH_4$. Peak c corresponds to the reduced cyclized products (diastereomers) with the observed mass 678.2 ± 0.0 Da (calcd 678.7 Da). Peak d corresponds to the reduced. (*) mixture of partial hydrolysis of the imine during column separation and water addition byproduct. (#) metal complex.

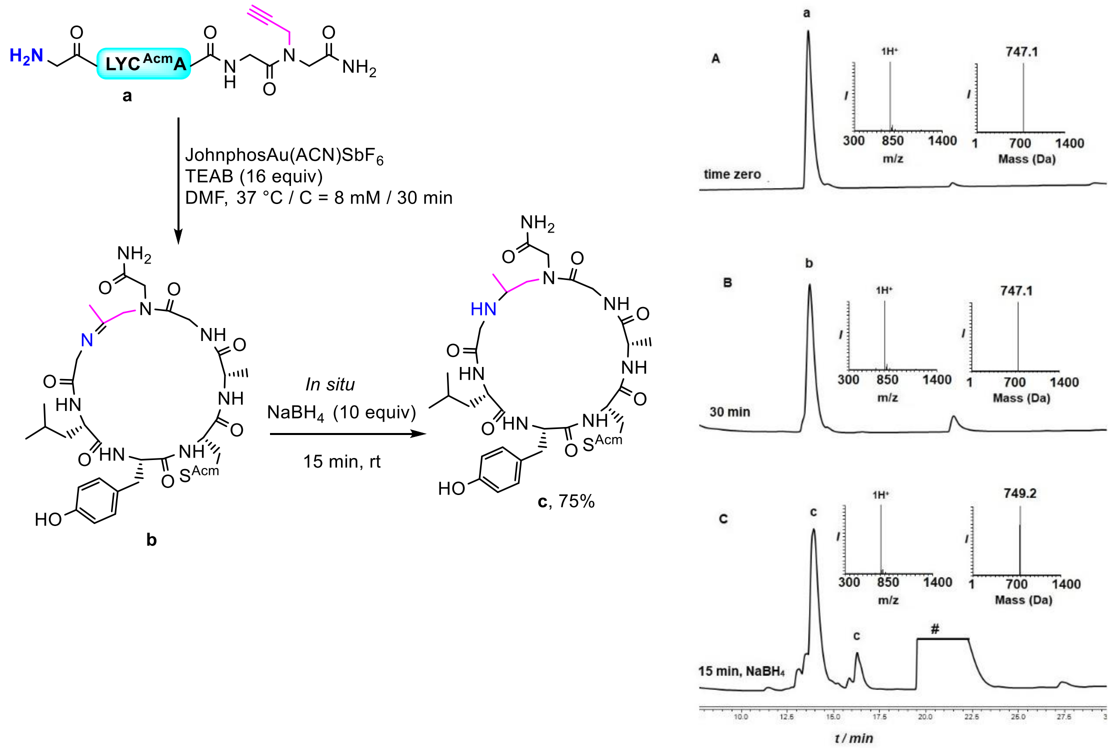

Figure S29: GLYC^{acm}AG(prop)G peptide reaction with JohnPhosAu(ACN)SbF$_6$: (A) Analytical HPLC and mass analysis of pure FGLYC^{acm}AG(prop)G. Peak a corresponds to FGLYC^{acm}AG(prop)G with the observed mass 747.1 ± 0.0 Da (calcd 747.7 Da) (r.t. = 13.50 min). (B) Analytical HPLC and mass analysis after 30 min incubation at 37 °C with JohnPhosAu(ACN)SbF$_6$. Peak b corresponds to cyclization product with the observed mass 747.1 ± 0.0 Da (calcd 747.7 Da) (r.t. = 13.50 min). (C) Analytical HPLC and mass analysis after 15 min incubation at rt with NaBH$_4$. Peak c corresponds to the reduced cyclized products (diastereomers) with the observed mass 749.2 ± 0.0 Da (calcd 749.7 Da). (#) metal complex.

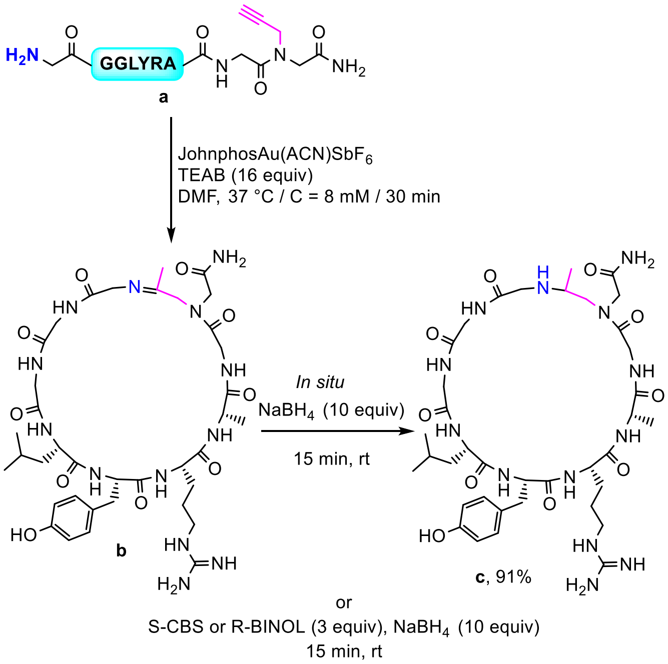

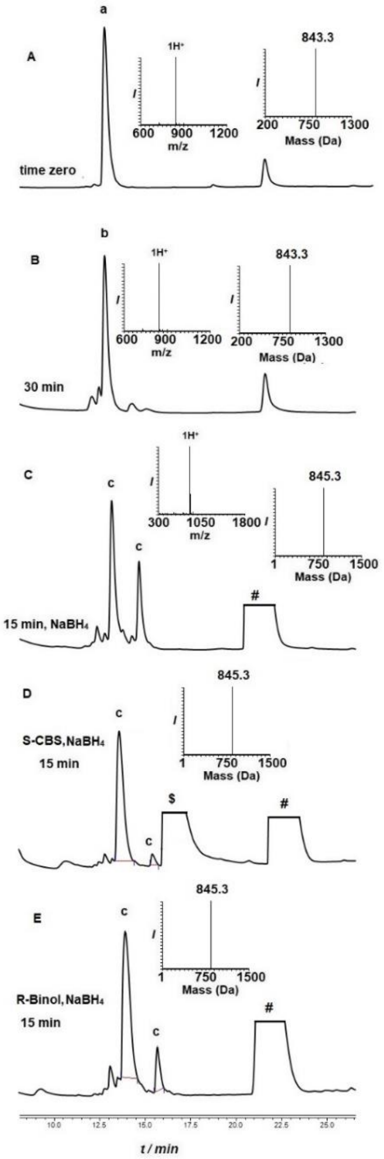

Figure S30: GGGLYRAG(prop)G peptide reaction with JohnPhosAu(ACN)SbF_6: (A) Analytical HPLC and mass analysis of pure GGGLYRAG(prop)G. Peak a corresponds to GGGLYRAG(prop)G with the observed mass 843.3 ± 0.0 Da (calcd 843.8 Da) (r.t. = 12.70 min). (B) Analytical HPLC and mass analysis after 30 min incubation at 37 °C with JohnPhosAu(ACN)SbF_6. Peak b corresponds to cyclization product with the observed mass 843.3 ± 0.0 Da (calcd 843.8 Da) (r.t. = 12.69 min). (C) Analytical HPLC and mass analysis after 15 min incubation at rt with $NaBH_4$. Peak c corresponds to the reduced cyclized products (diastereomers) with the observed mass 845.3 ± 0.0 Da (calcd 845.8 Da). (D) Analytical HPLC and mass analysis after 15 min incubation at rt with S-CBS and $NaBH_4$. Peak c corresponds to the reduced cyclized products (diastereomers) with the observed mass 845.3 ± 0.0 Da (calcd 845.8 Da). (E) Analytical HPLC and mass analysis after 15 min incubation at rt with R-BINOL and $NaBH_4$. Peak c corresponds to the reduced cyclized products (diastereomers) with the observed mass 845.3 ± 0.0 Da (calcd 845.8 Da). (#) metal complex. ($) ligand related material.

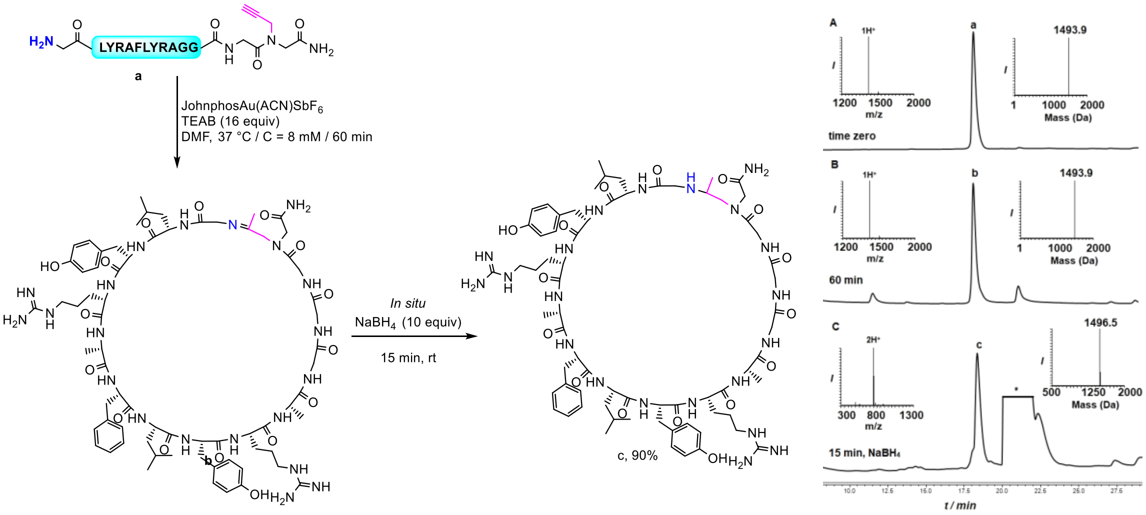

Figure S31: GLYRAFLYRAGGG(prop)G peptide reaction with JohnPhosAu(ACN)SbF_6: (A) Analytical HPLC and mass analysis of pure GLRAFLYRAGGG(prop)G. Peak a corresponds to GLRAFLYRAGGG(prop)G with the observed mass 1493.9 ± 0.0 Da (calcd 1494.6 Da) (r.t. = 18.53 min). (B) Analytical HPLC and mass analysis after 60 min incubation at 37 °C with JohnPhosAu(ACN)SbF_6. Peak b corresponds to cyclization product with the observed mass 1493.9 ± 0.0 Da (calcd 1494.6 Da) (r.t. = 18.53 min). (C) Analytical HPLC and mass analysis after 15 min incubation at rt with $NaBH_4$. Peak c corresponds to the reduced cyclized products (diastereomers) with the observed mass 1496.5 ± 0.0 Da (calcd 1496.6 Da). (*) metal complex.

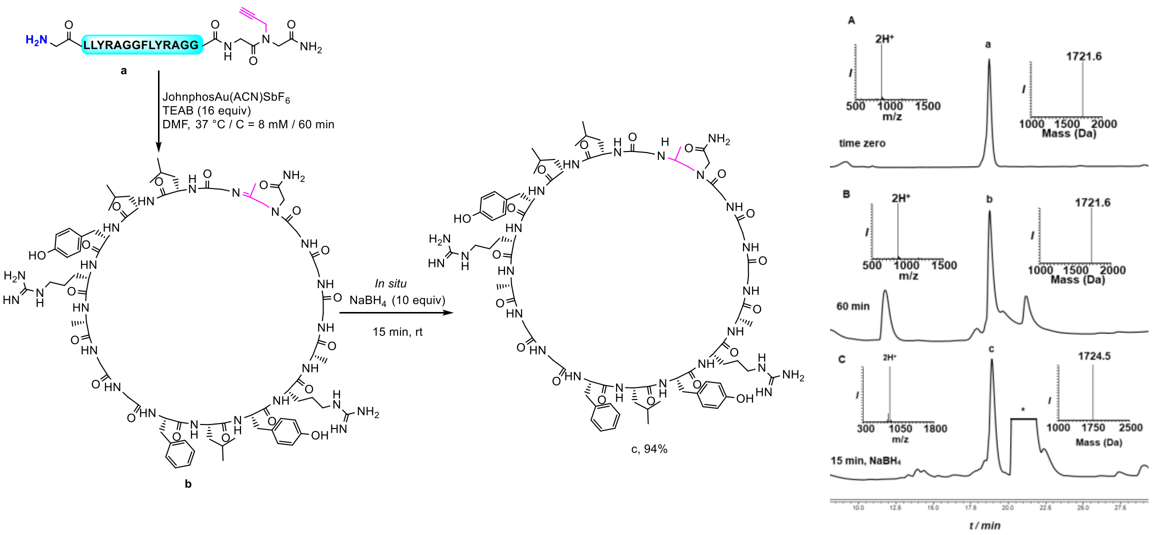

Figure S32: GLLYRAGGFLYRAGGG(prop)G peptide reaction with JohnPhosAu(ACN)SbF_6: (A) Analytical HPLC and mass analysis of pure GLLYRAGGFLYRAGGG(prop)G. Peak a corresponds to GLLYRAGGFLYRAGGG(prop)G with the observed mass 1721.6 ± 0.0 Da (calcd 1721.9 Da) (r.t. = 18.80 min). (B) Analytical HPLC and mass analysis after 60 min incubation at 37 °C with JohnPhosAu(ACN)SbF_6. Peak b corresponds to cyclization product with the observed mass 1721.6 ± 0.0 Da (calcd 1721.9 Da) (r.t. = 18.80 min). (C) Analytical HPLC and mass analysis after 15 min incubation at rt with $NaBH_4$. Peak c corresponds to the reduced cyclized products (diastereomers) with the observed mass 1724.5 ± 0.0 Da (calcd 1723.9 Da), HRMS (ESI+): $[M+H]^+$ calcd. 1723.9396 and found 1723.9396. (*) metal complex.

Synthesis of $(Me)_2$N-GLYAG(prop)G & (Me)2N-GLYKRAG(prop)G peptides

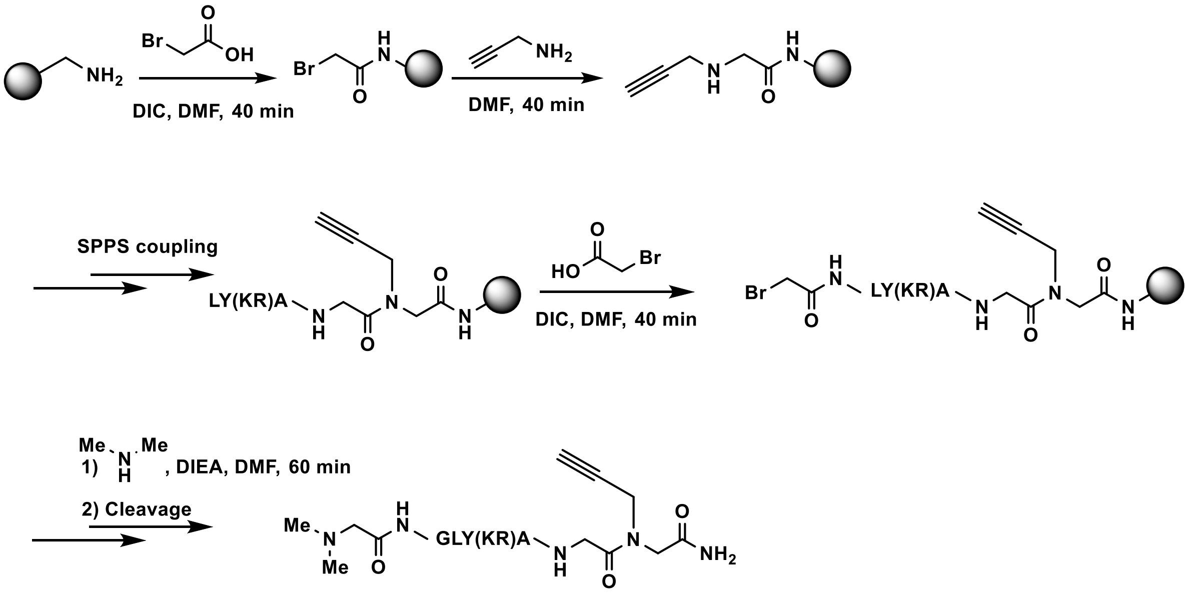

Firstly, the resin was treated with 20% piperidine in DMF for Fmoc-deprotection (3x5ml) followed by bromoacetic acid (BAA) coupling. Then, BAA (20 equiv.) was activated using 20 equiv. DIC in DMF for 20 min at room temperature. Activated BAA was added to the resin and allowed it to couple for 40 min. Subsequently, propargyl amine (20 equiv.) in DMF (900 µl, ~1 M) was added and the reaction was left for 40 min at room temperature. The Next amino acid was double coupled using 20 equiv. Fmoc-Gly-OH and 20 equiv. HATU and 40 equiv. DIEA for 1 hr at room temperature. The remaining amino acids were coupled on as described above. Following the Fmoc-deprotection of the last amino acid, BAA was coupled as described above. Subsequently, *N*,*N*-Dimethylamine (20 equiv.), DIEA (8 equiv) in DMF (900 µl, ~1 M) was added and the reaction was left for 60 min. Finally, the peptide was cleaved from the resin with the cleavage cocktail and purified using preparative HPLC C18 column with a gradient of 10-50% B over 40 min.

Scope of lysine containing peptides

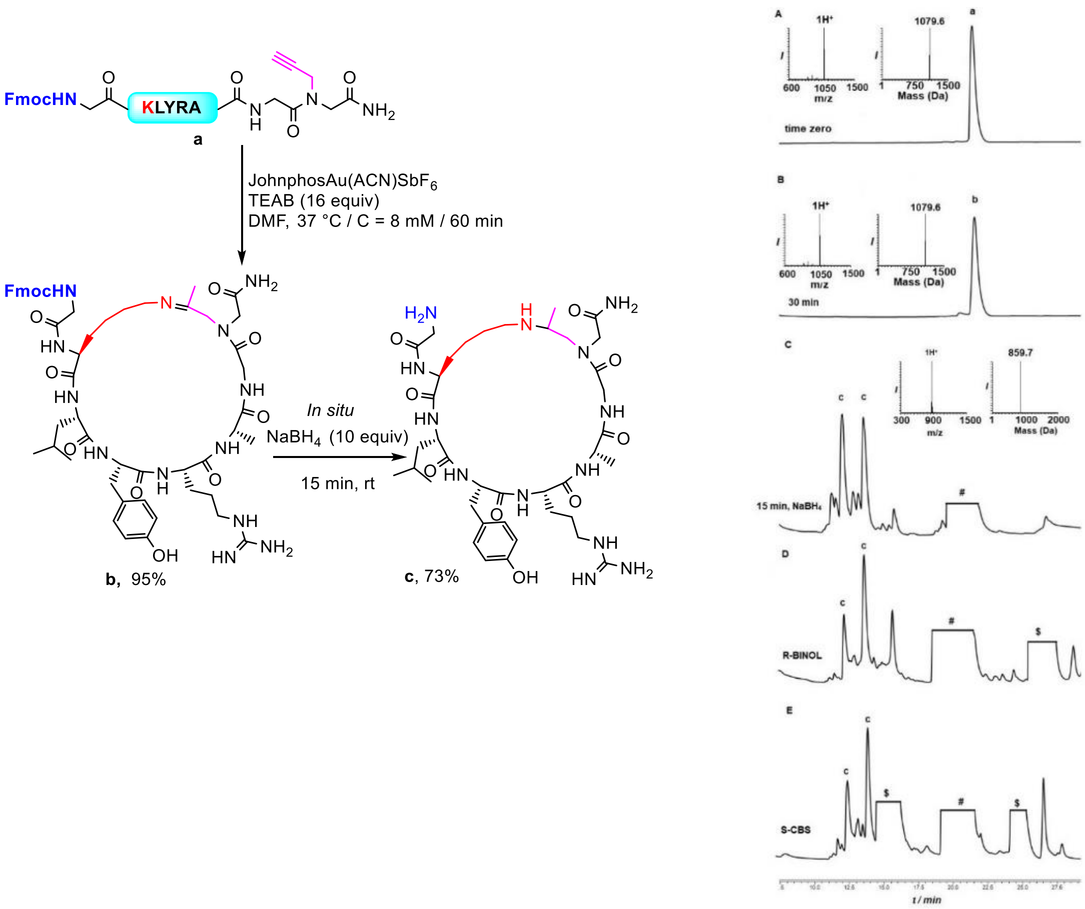

Figure S33: Fmoc-GKLYRAG(prop)G peptide reaction with JohnPhosAu(ACN)SbF_6: (A) Analytical HPLC and mass analysis of pure Fmoc-GKLYRAG(prop)G. Peak a corresponds to Fmoc-GKLYRAG(prop)G with the observed mass 1079.6 ± 0.0 Da (calcd 1079.9 Da) (r.t = 21.70 min). (B) Analytical HPLC and mass analysis after 60 min incubation at 37 °C with JohnPhosAu(ACN)SbF_6. Peak b corresponds to cyclization product with the observed mass 1079.6 ± 0.0 Da (calcd 1079.9 Da) (r.t = 21.60 min). (C) Analytical HPLC and mass analysis after 15 min incubation at rt with $NaBH_4$. Peak c corresponds to the reduced cyclized products (diastereomers) with the observed mass 859.7 ± 0.0 Da (calcd 859.9 Da) (Fmoc is removed during reduction), HRMS (ESI+): $[M+H]^+$ calcd. 860.5106 and found 860.5106. (D) Analytical HPLC and mass analysis after 15 min incubation at rt with R-BINOL and $NaBH_4$. Peak c corresponds to the reduced cyclized products (diastereomers) with the observed mass 859.7 ± 0.0 Da (calcd 859.9 Da). (E) Analytical HPLC and mass analysis after 15 min incubation at rt with S-CBS and $NaBH_4$. Peak c corresponds to the reduced cyclized products (diastereomers) with the observed mass 859.7 ± 0.0 Da (calcd 859.9 Da). (#) metal complex. ($) ligand related material.

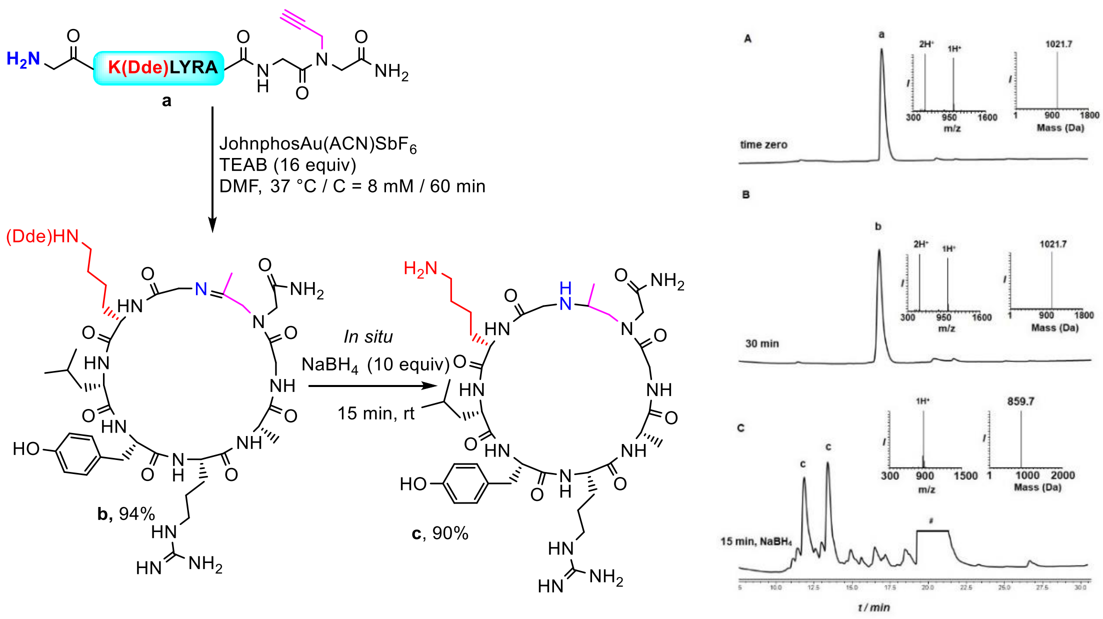

Figure S34: GK(Dde)LYRAG(prop)G peptide reaction with JohnPhosAu(ACN)SbF_6: (A) Analytical HPLC and mass analysis of pure GK(Dde)LYRAG(prop)G. Peak a corresponds to GK(Dde)LYRAG(prop)G with the observed mass 1021.7 ± 0.0 Da (calcd 1021.9 Da) (r.t = 16.93 min). (B) Analytical HPLC and mass analysis after 60 min incubation at 37 °C with JohnPhosAu(ACN)SbF_6. Peak b corresponds to cyclization product with the observed mass 1021.7 ± 0.0 Da (calcd 1021.9 Da) (r.t = 16.80 min). (C) Analytical HPLC and mass analysis after 15 min incubation at rt with $NaBH_4$. Peak c corresponds to the reduced cyclized products (diastereomers) with the observed mass 859.7 ± 0.0 Da (calcd 859.9 Da) (Dde protection is removed during reduction). (#) metal complex.

Synthesis of thioether linked peptide 3

Sequence for **3**: mClBz-GWFDDLYWFVAYC

mClBz-GWFDDLYWFVAYC

3

The synthesis proceeded according to the reported method[1].

Synthesis of propargylated peptide 4

Sequence: GWFDDLYWFVAY(prop)G

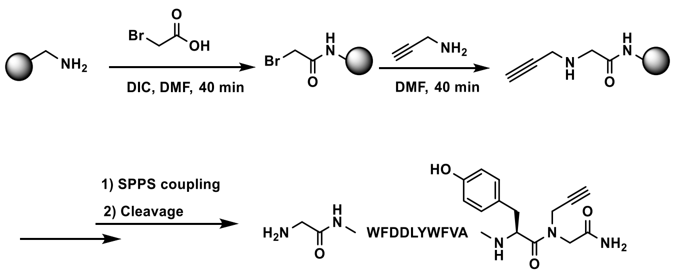

The synthesis performed as described before in general procedure for synthesis of AA-G(prop)G.

Cyclization of propargylated peptide 4 to cyclic peptides 5 and 6 with Au(I)

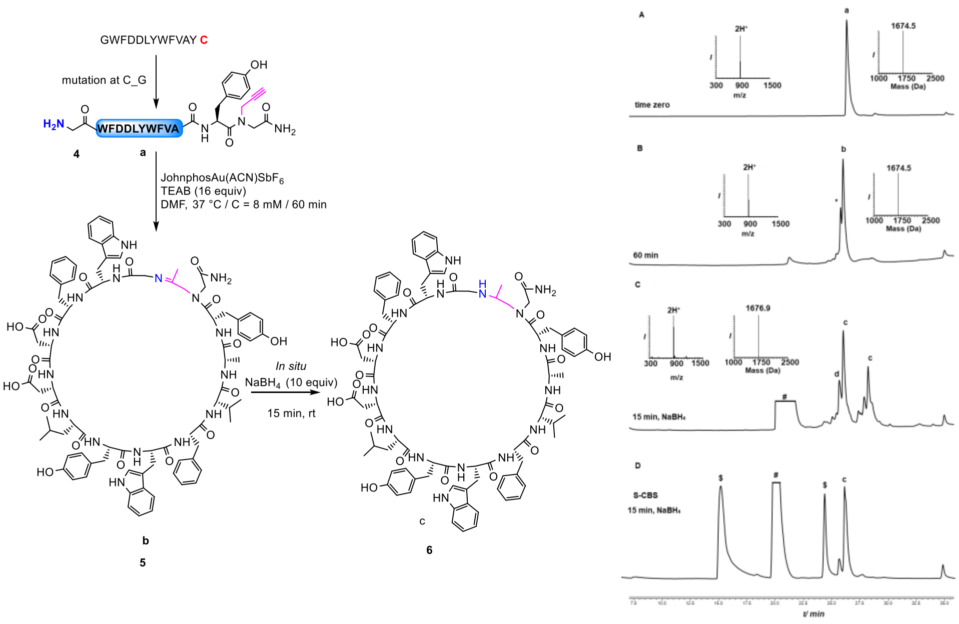

Figure S35: GWFDDLYWFVAY(prop)G, **4**, peptide reaction with JohnPhosAu(ACN)SbF_6: (A) Analytical HPLC and mass analysis of pure GWFDDLYWFVAY(prop)G, **5**. Peak a corresponds to GWFDDLYWFVAY(prop)G with the observed mass 1674.5 ± 0.0 Da (calcd 1675.8 Da) (r.t = 26.18 min). (B) Analytical HPLC and mass analysis after 60 min incubation at 37 °C with JohnPhosAu(ACN)SbF_6. Peak b corresponds to cyclization product with the observed mass 1674.5 ± 0.0 Da (calcd 1675.8 Da) (r.t = 26.17 min). (C) Analytical HPLC and mass analysis after 15 min incubation at rt with $NaBH_4$. Peak c corresponds to the reduced cyclized products (diastereomers) with the observed mass 1676.9 ± 0.0 Da (calcd 1677.8 Da), HRMS (ESI+): $[M]^+$ calcd. 1676.7664 and found 1676.7664. (D) Analytical HPLC and mass analysis after 15 min incubation at rt with S-CBS and $NaBH_4$. Peak c corresponds to the reduced cyclized products (diastereomers) with the observed mass 1676.9 ± 0.0 Da (calcd 1677.8 Da). (#) metal complex. ($) ligand related material.

Synthesis of peptide 4a

Sequence: GWFDDLYWFVAY(prop)GAC^{Acm} (peptide sequence of **4** + AC^{acm})

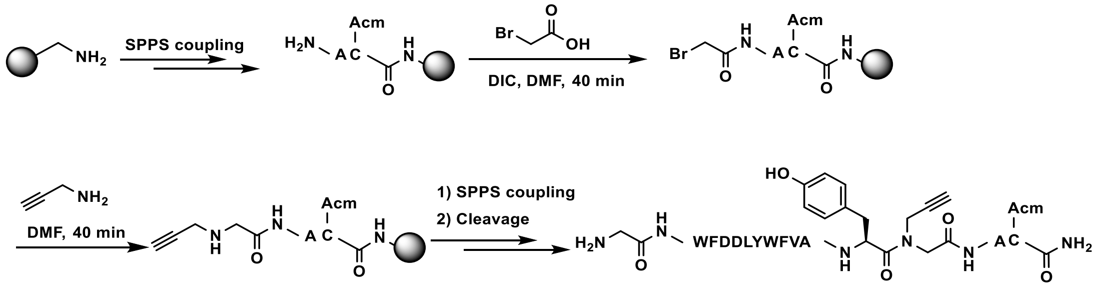

The synthesis performed as described before in general procedure for synthesis of AA-G(prop)G.

Cyclization of propargylated peptide 4a using gold to cyclic peptide 7 with Au(I)

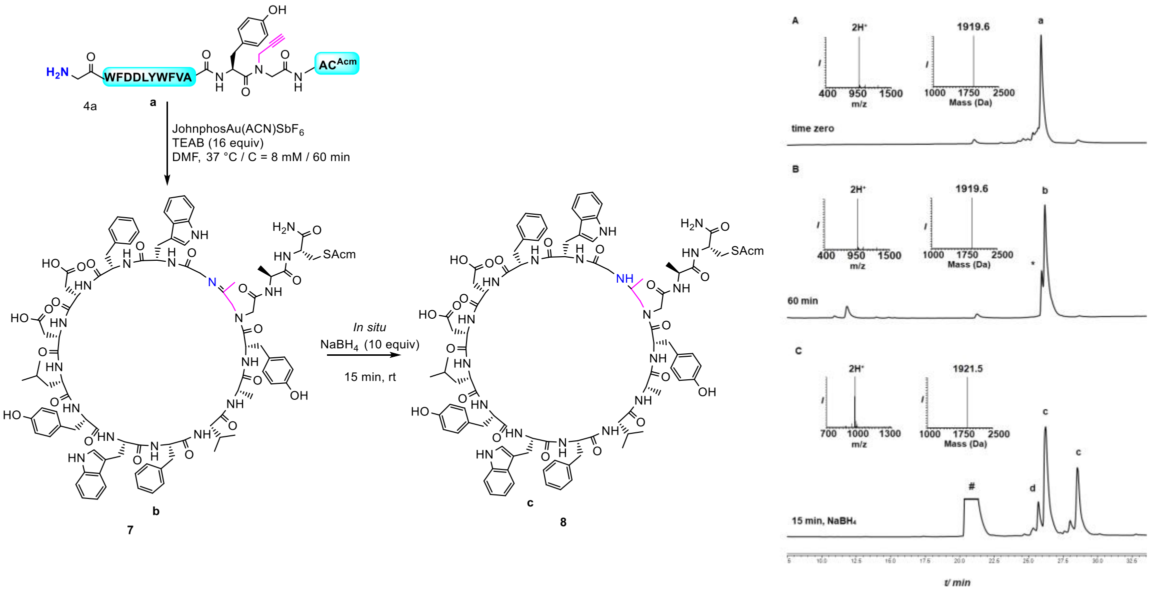

Figure S35: Synthesis of cyclic peptide **7**: (A) Analytical HPLC and mass analysis of pure GWFDDLYWFVAY(prop)GAC^{Acm}. Peak a corresponds to GWFDDLYWFVAY(prop)GAC^{Acm} with the observed mass 1919.6 ± 0.0 Da (calcd 1922.0 Da) (r.t = 25.96 min). (B) Analytical HPLC and mass analysis after 20 min incubation at 37 °C with JohnPhosAu(ACN)SbF$_6$. Peak b corresponds to the product with the observed mass 1919.6 ± 0.0 Da (calcd 1922.0 Da) (r.t = 25.95 min). Analytical HPLC and mass analysis after 15 min incubation at rt with NaBH$_4$. Peak c corresponds to the reduced cyclized products (diastereomers) with the observed mass 1921.5 ± 0.0 Da (calcd 1921.8 Da).

Synthesis of thioether linked peptide 3a

Sequence for **3a:**

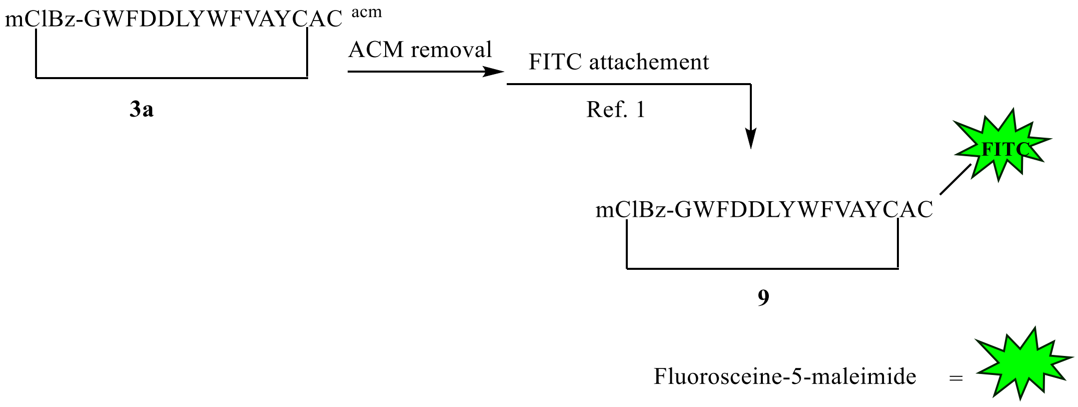

The synthesis proceeded according to the reported method.[1]

Synthesis of cyclic peptide 10

The synthesis proceeded according to the reported method.[1]

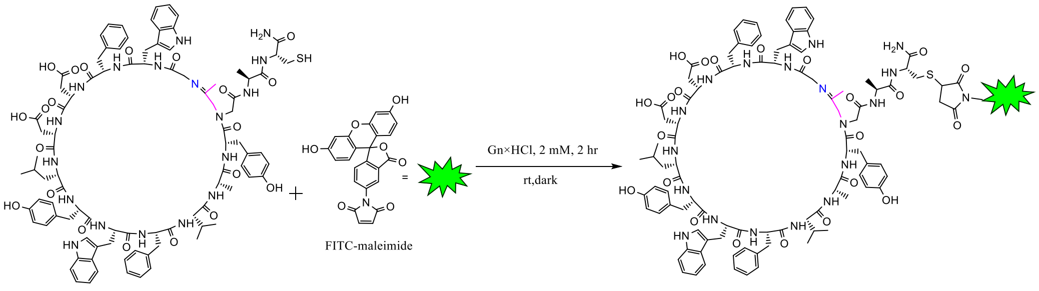

10

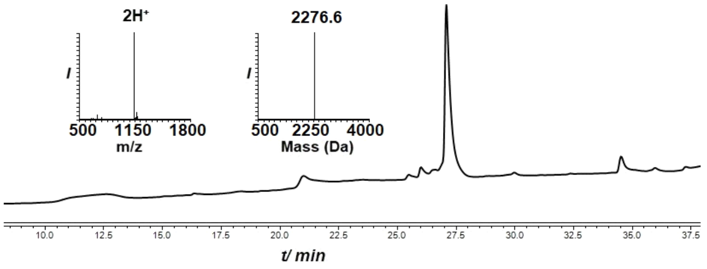

Figure S36: Synthesis of cyclic peptides **10**. Analytical HPLC and mass analysis of purified peptide **10** with the observed mass 2276.6 ± 0.0 Da (calcd 2277.0 Da).

Au(I) mediated on resin reaction (Solid Phase Cyclization)

Solid phase cyclization was performed using NH_2-GLYRAG(prop)G peptide under our described conditions on 0.01 mmol scale, incubated for 60 min in eppendorf and 15 min reduction with 10 equiv. $NaBH_4$ and followed by resin global cleavage.

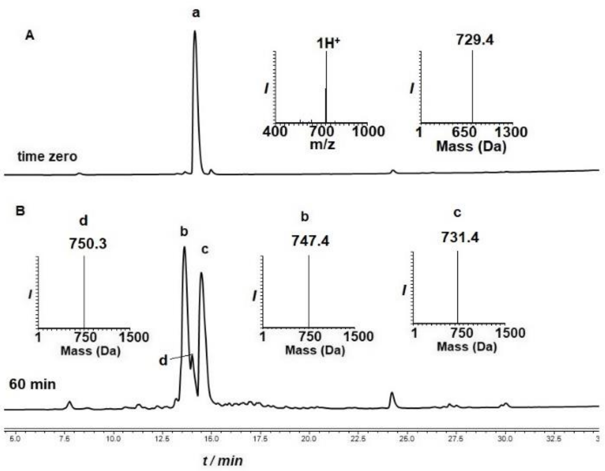

Figure S37: GLYRAG(prop)G on resin peptide reaction with JohnPhosAu(ACN)SbF_6: (A) Analytical HPLC and mass analysis of pure GLYRAG(prop)G . Peak a corresponds to GLYRAG(prop)G with the observed mass 729.4 ± 0.0 Da (calcd 728.7 Da). (B) Analytical HPLC and mass analysis after 60 min incubation at 37 °C with JohnPhosAu(ACN)SbF_6 and followed by 15 min incubation at rt with $NaBH_4$ and resin global cleavage. Peak b corresponds to water addition product with the observed mass 747.4 ± 0.0 Da (calcd 746.7 Da). Peak c corresponds to the reduced cyclized product with the observed mass 731.4 ± 0.0 Da (calcd 731.4 Da). Peak d corresponds to reduced water addition product with the observed mass 750.3 ± 0.0 Da (calcd 749.4 Da).

Flourescence based competitive assay against K48-Di-Ub chains

Each well of streptavidin-coated microplate (96 well plate) was washed (3 X 100 µl) with HEPES buffer (50 mM HEPES, NaCl (150 mM), 0.1% Tween, pH = 7.3). Next, each well was incubated with 1 µg of biotin-di-UbK48 in 100 µl of HEPES (50 mM HEPES, NaCl (150 mM), 0.1% Tween, pH = 7.3) for 30 minutes at room temperature (RT). Additional wells were kept free of biotin-di-UbK48 relative to wells with biotin-di-UbK48 for subsequent blank subtraction. Following washing step (each well washed with 3X100 µl of HEPES buffer), unlabeled candidate peptides **5** and **6**, including peptide-standard (**3**) each in 100 µl of HEPES buffer (50 mM HEPES, NaCl (150

mM), 0.1% Tween, pH = 7.3), were incubated in an excess amount (5 molar equiv relative to biotin-tetra-UbK48) for 30 minutes at RT to saturate binding to the target. Following the washing step (each well washed with 3X100 μl with HEPES buffer (50 mM HEPES, NaCl (150 mM), 0.1% Tween, pH = 7.3)) FITC labeled peptide-standard (**9**) in 100 μl of HEPES Buffer (50 mM HEPES, NaCl (150 mM), 0.1% Tween, pH = 7.3) was incubated in each well for 30 minutes at RT in 1 molar equivalent relative to biotin-tetra-UbK48 or biotin-tetra-UbK48 to compete with the unlabeled peptides. Finally, following washing step (each well washed with 3X100 μl with HEPES buffer (50 mM HEPES, NaCl (150 mM), 0.1% Tween, pH = 7.3)), each well was treated with 100 μl of 6M Gn.HCl at RT for 30 minutes to release the bound peptides. Then, the solution with the released peptides was transferred to Nunc black 96-well plate and fluorescence was measured (λ_{ex} = 480 nm and λ_{em}= 525 nm). The obtained values are then normalized, and compared with peptide standard, while the fluorescence signal is inversely proportional to the binding affinity of the peptide. The calculations of relative change in binding were performed according to the formula: Y = -[1-(ab)]x100, where Y is the change in the signal relative to the standard thioether linked peptides (**3**) (in percentage); a is signal measured for each peptide variant (in relative fluorescence units); b is signal measured for **3**.

Kd determination

To determine dissociation constant (Kd) of labeled peptide (**10**), different concentrations of the labeled peptide were incubated, following immobilization step of biotin-di-UbK48. Then, the bound cyclic peptides are released with 6M Gn.HCl, followed by fluorescence measurement (λex = 480 nm and λem = 525 nm). After normalization, obtained binding curve was used for kinetic determination of Kd

Cell uptake studies

To investigate the cellular delivery efficiency of the new cyclic peptide HeLa (CCL-2™) cells were seeded on ibidi poly-L-lysine (PLL) 8 well µ-slides in $2.5 \times 10^4 \frac{cells}{well}$ and were allowed to reach ~90% confluency over 24h. Cells were washed three times with warm PBS and serum-free DMEM (high glucose) followed by incubation for 1h with warm PBS/serum-free medium containing containing 2.5, 5, and 10 µM of fluorescently labeled cyclic peptides (**9** and **10**). Cells were then washed three times with warm PBS followed by adding 0.1 mg/ml heparin sulfate in PBS and incubating for 5min. Next, cells were washed with phenol red free optical medium and stained using manufacturer standard protocols with Hoechst (2 µg/ml) for imaging using confocal laser scanning microscope (CLSM-710). The FITC and Hoechst tags were imaged using 488 nm and 405 nm lasers respectively.

Apoptosis studies

To evaluate the apoptosis resulted from the cyclic peptides, Cytek Aurora, a spectral flow cytometer equipped with four lasers (405, 488, 561, 635 nm) having the technology to allow the detection of the full emission spectrum.

HeLa cells, seeded on were incubated with 2.5 µM of both cyclic peptides for 24h at 37°C with 5% CO_2. Following the treatments, cells were washed twice with PBS (calcium and magnesium free) and then trypsinized. After trypsinization, cells were harvested and washed once with full DMEM and once with PBS (calcium and magnesium free). Next, cells were resuspended in binding buffer (supplied with the apoptosis kit) and stained with Annexin V-FITC and propidium iodide (PI) according to the manufacture's protocol given with the apoptosis detection kit (BD Biosciences). Cells were then processed at a flow rate of 15 µL/min. Apoptotic cells were identified from % of the population having signals with FITC +ve, PI -ve (early apoptosis) FITC +ve, PI +ve (late apoptosis). MG-132 and untreated cells were taken as positive and negative controls, respectively.

By evaluating the results from the flow analyzer, it could be seen that the population of cancer cells undergoing apoptosis with **5** is nearly three-fold higher to the cyclic peptide with thioether linkage (**3**).

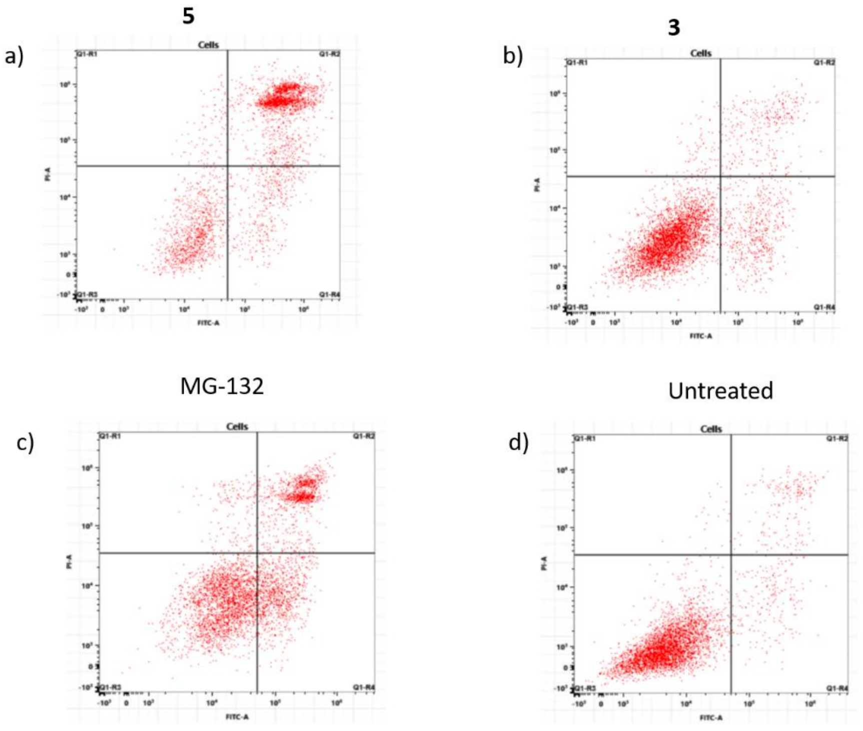

Figure S38: Early and late apoptosis of HeLa cells induced by **5** & **3**. The figure represents individual dot plots of (a) Cells treated for 24 h with 2.5 μM of **5**. (b) Cells treated for 24 h with 2.5 μM of **3**. (c). Cells were treated for 24 h with 2.5 μM of MG132. (d) Untreated cells.

Reference

1. Vamisetti, G. B.; Meledin, R.; Nawatha, M.; Suga, H.; Brik, A. The Development of a Fluorescence-Based Competitive Assay Enabled the Discovery of Dimeric Cyclic Peptide Modulators of Ubiquitin Chains. *Angew. Chemie - Int. Ed.* **2021**, *60* (13), 7018.

NMR data

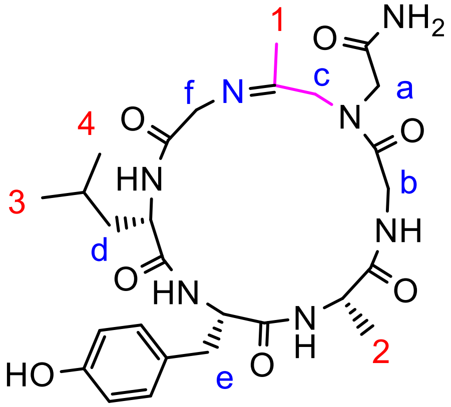

^{1}H NMR (400 MHz, MeOD–d$_4$): δ 6.96–6.94 (m, ArH), 6.594–6.57 (m, ArH), 4.48–4.41 (m), 4.34–4.22 (m), 4.17–4.11 (m), 4.09–4.00 (m), 3.91 (s), 3.00–2.90 (m), 2.81–2.72 (m), 2.62–2.61 (m), 1.52–1.44 (m), 1.40–1.33 (m), 1.26–1.24 (m, Leu –CH, –2CH_3 peaks merged (1 and 2)), 0.83–0.77 (m, –2CH_3 (3 and 4)); **^{13}C NMR (101 MHz, MeOD–d$_4$)**: δ 173.0, 169.4, 130.1, 114.9, 114.8, 73.9, 73.1, 54.9, 52.3, 52.2, 48.9, 40.6, 40.5, 40.1, 37.3, 36.5, 24.4, 21.9, 20.5, 16.6 (4x–CH_3, numbered 1-4 in red).

DEPT-135 (101 MHz, MeOD–d$_4$): 130.1, 114.9, 114.8, 54.92, 54.90, 52.3, 52.2, 48.9, -40.6, -40.5, -40.1, -37.3, -36.5, -35.5 (6x –CH_2, numbered a-f in blue), 24.4, 21.9, 20.5, 16.6 (4x–CH_3, numbered 1-4 in red).

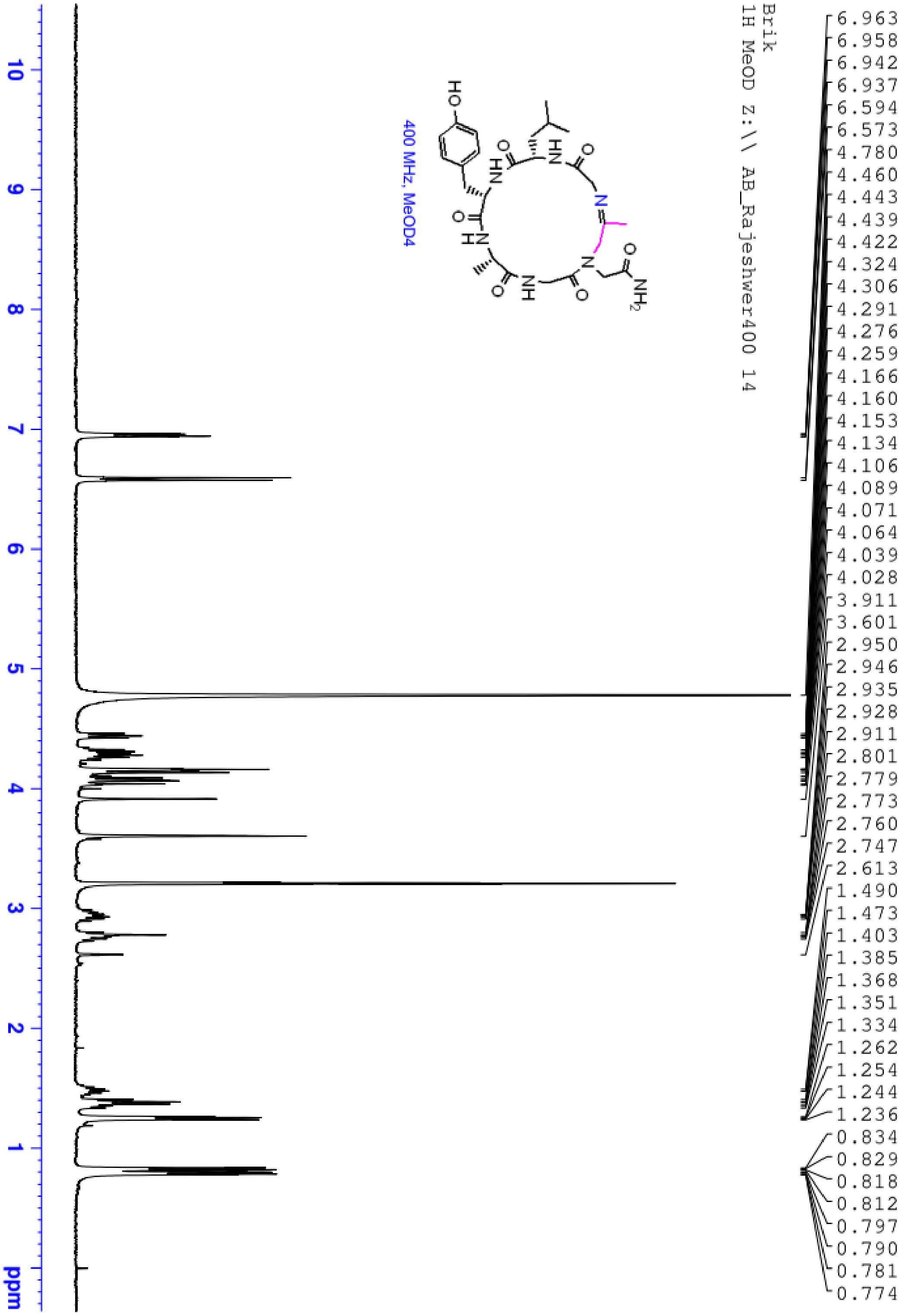
Brik
1H MeOD Z:\\ AB_Rajeshwer400 14
400 MHz, MeOD4
6.963
6.958
6.942
6.937
6.594
6.573
4.780
4.460
4.443
4.439
4.422
4.324
4.306
4.291
4.276
4.259
4.166
4.160
4.153
4.134
4.106
4.089
4.071
4.064
4.039
4.028
3.911
3.601
2.950
2.946
2.935
2.928
2.911
2.801
2.779
2.773
2.760
2.747
2.613
1.490
1.473
1.403
1.385
1.368
1.351
1.334
1.262
1.254
1.244
1.236
0.834
0.829
0.818
0.812
0.797
0.790
0.781
0.774
HO
HN
O
NH
N
NH₂
10
9
8
7
6
5
4
3
2
1
ppm

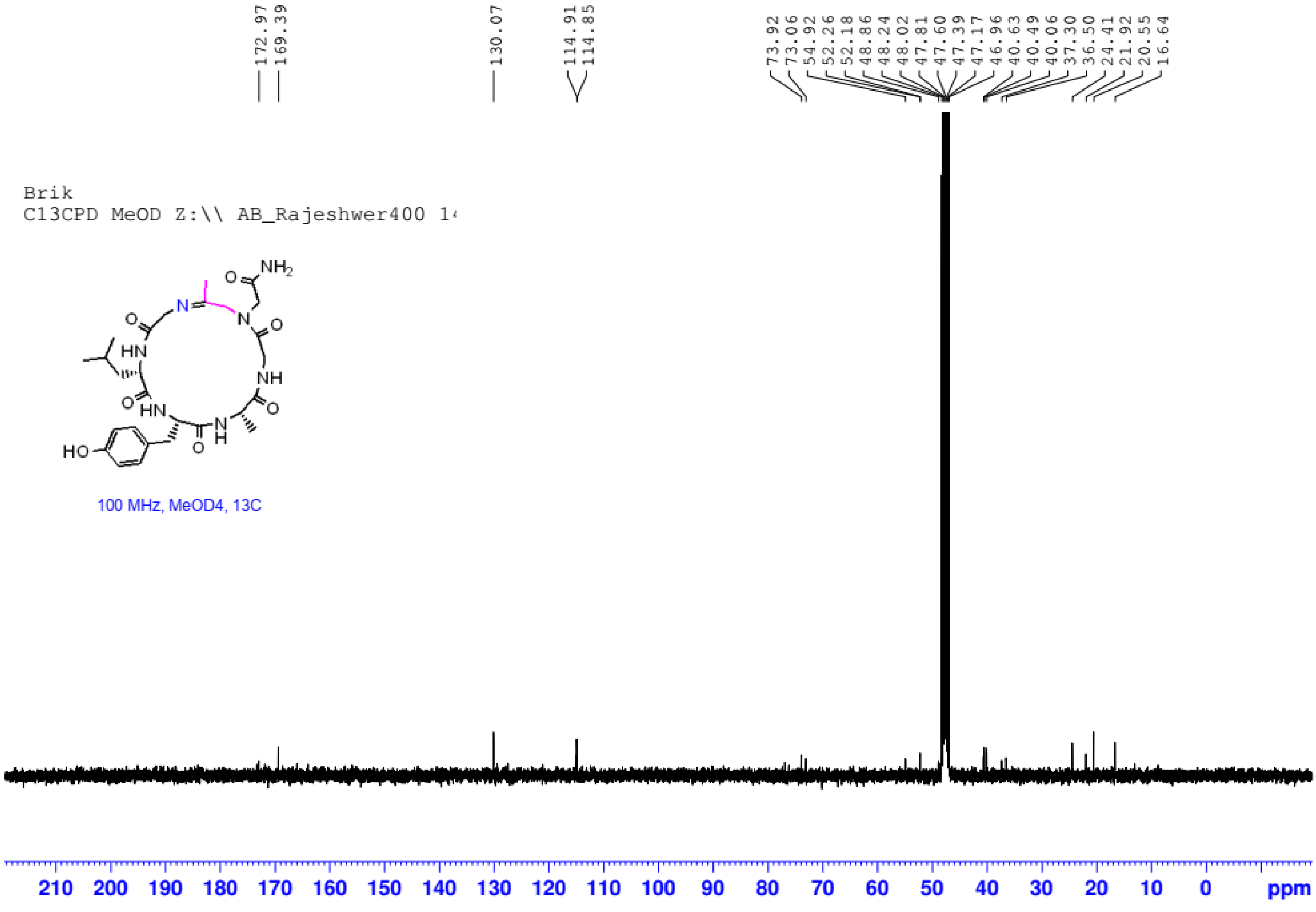
Brik
C13CPD MeOD Z:\\ AB_Rajeshwer400 1
100 MHz, MeOD4, 13C
172.97
169.39
130.07
114.91
114.85
73.92
73.06
54.92
52.26
52.18
48.86
48.24
48.02
47.81
47.60
47.39
47.17
46.96
40.63
40.49
40.06
37.30
36.50
24.41
21.92
20.55
16.64
210 200 190 180 170 160 150 140 130 120 110 100 90 80 70 60 50 40 30 20 10 0 ppm

1H-1H COSY and HMQC analysis: Key correlations:

In the region of 1.26–1.4, three 3 diagonal spots present corresponding to Leu –CH (3), and two $–CH_3$ peaks (1 and 2) (circled in red color). The Leu –CH peaks were in correlation with methyl peaks evident from the presence off-diagonal spot. In 0.83–0.77 region, 2 methyl peaks of Leu are present (a and b) (circled in blue color). These correlations also can be seen in HMQC and 4 clear peaks for methyl groups present.

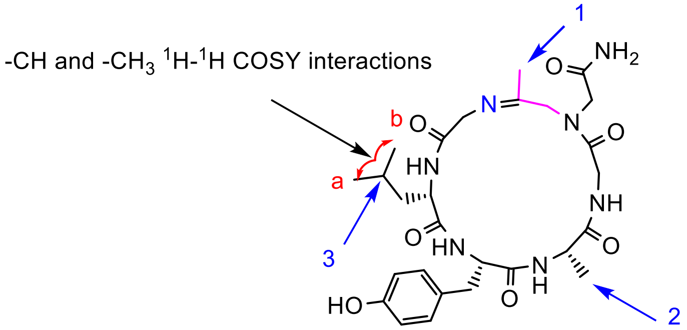

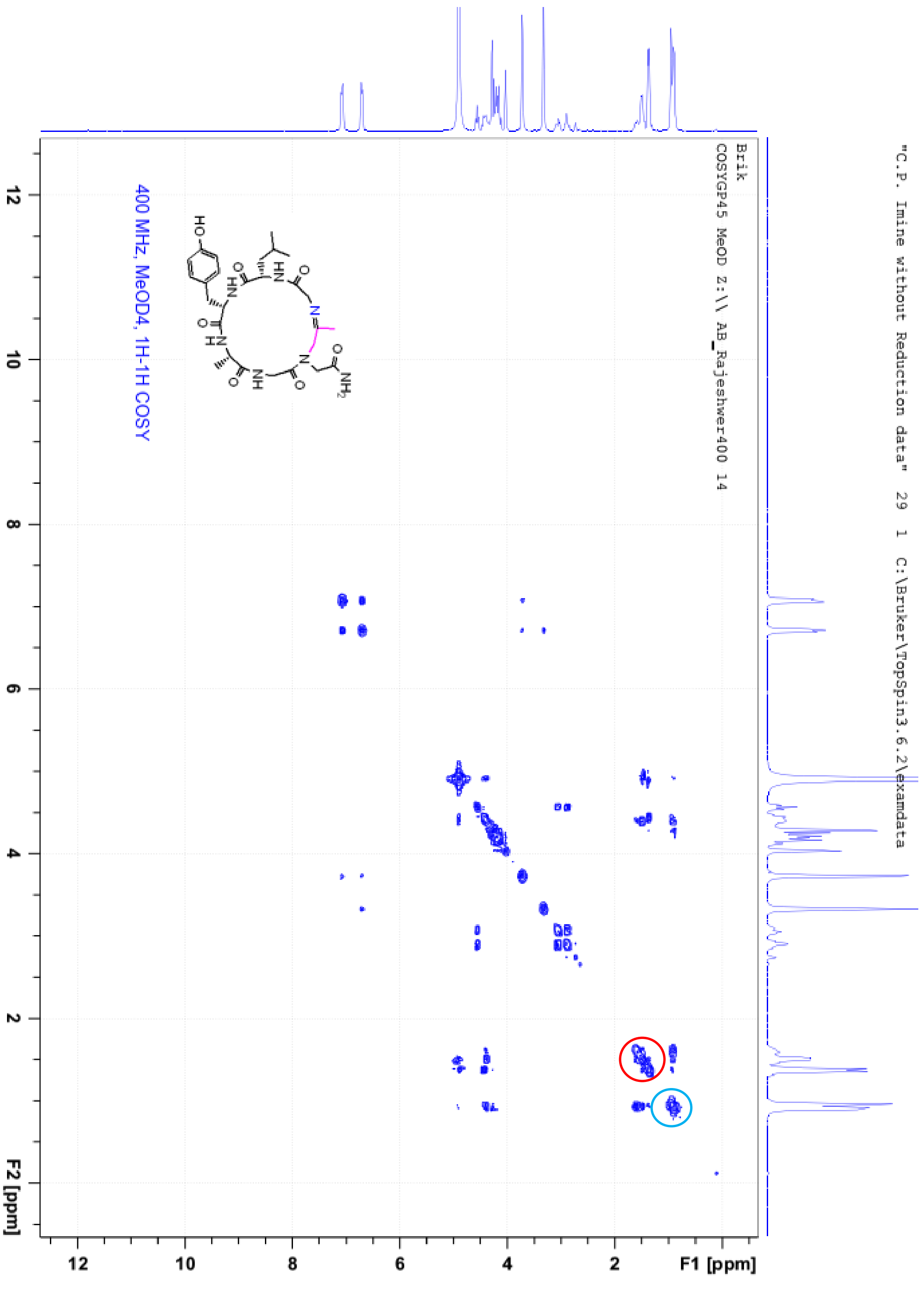
"C.P. Imine without Reduction data" 29 1 C:\Bruker\TopSpin3.6.2\examdata
Brik
COSYGP45 MeOD Z:\\ AB_Rajeshwer400 14
400 MHz, MeOD4, 1H-1H COSY
F1 [ppm]
F2 [ppm]

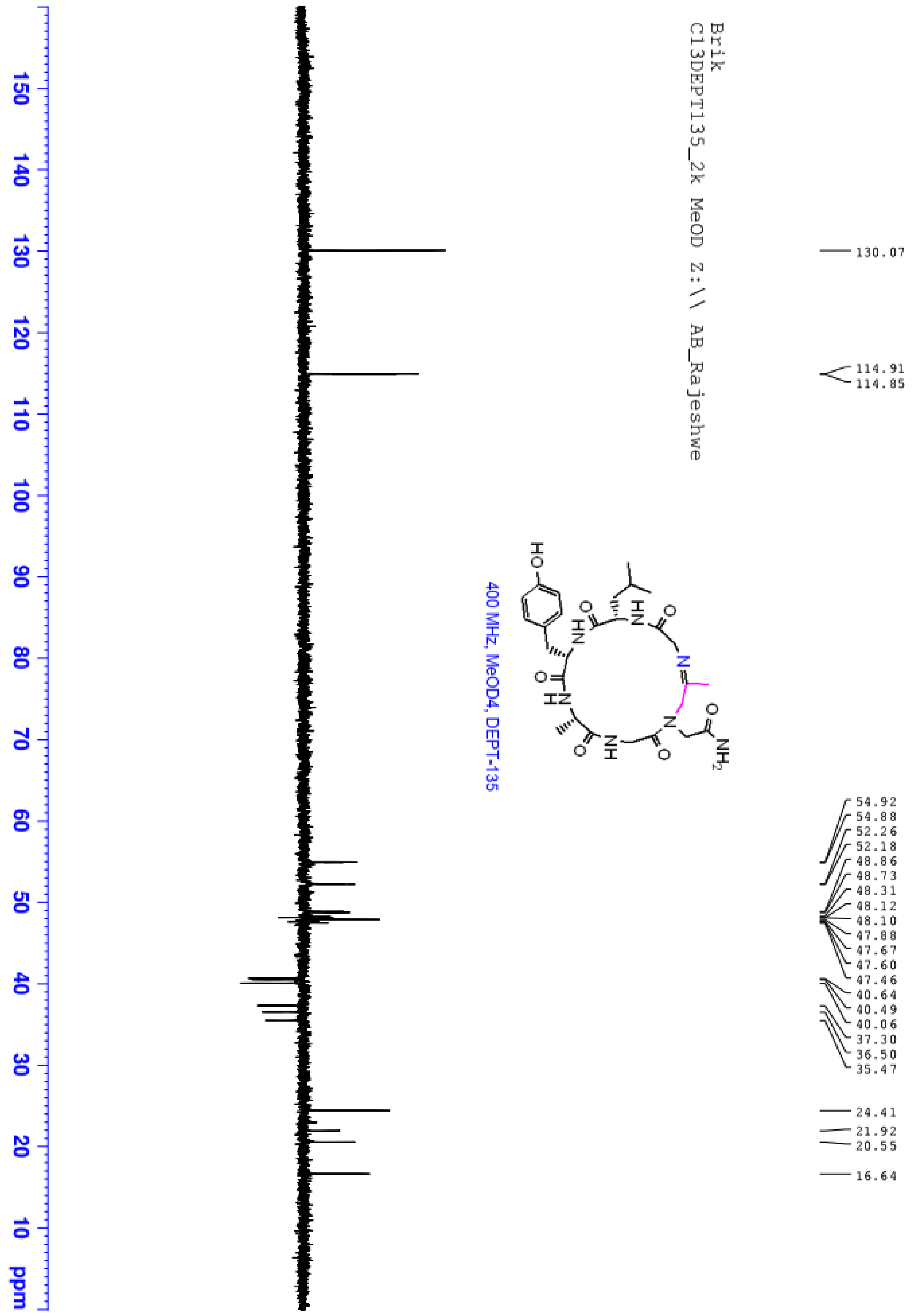
Brik
C13DEPT135_2k MeOD Z:\\ AB_Rajeshwe
400 MHz, MeOD4, DEPT-135
130.07
114.91
114.85
54.92
54.88
52.26
52.18
48.86
48.73
48.31
48.12
48.10
47.88
47.67
47.60
47.46
40.64
40.49
40.06
37.30
36.50
35.47
24.41
21.92
20.55
16.64
150
140
130
120
110
100
90
80
70
60
50
40
30
20
10
ppm

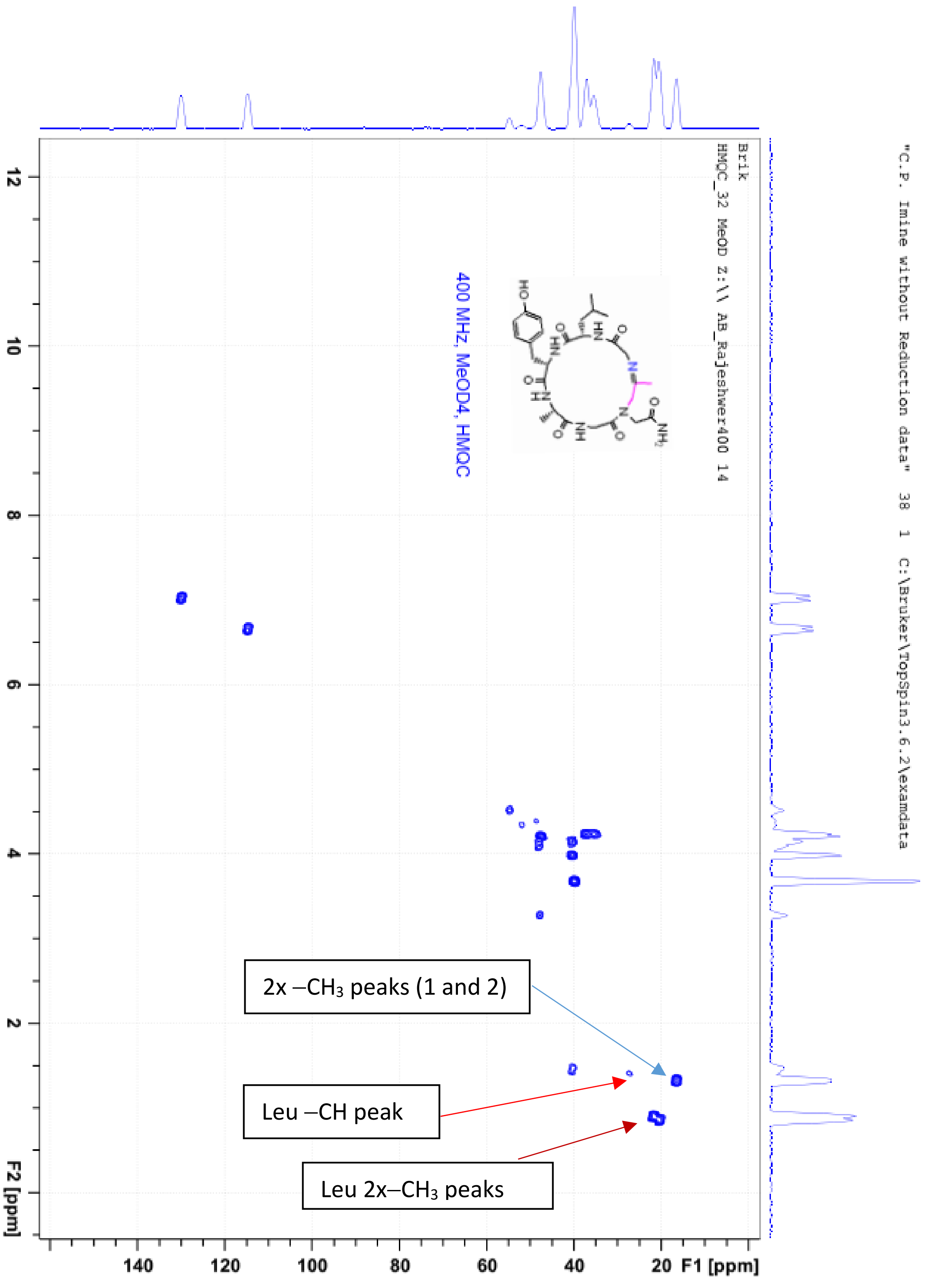
"C.P. Imine without Reduction data" 38 1 C:\Bruker\TopSpin3.6.2\examdata
Brik
HMQC_32 MeOD Z:\\ AB_Rajeshwer400 14
400 MHz, MeOD4, HMQC
2x –CH3 peaks (1 and 2)
Leu –CH peak
Leu 2x–CH3 peaks
F2 [ppm]
F1 [ppm]

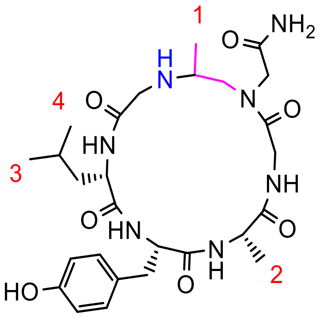

^{1}H NMR (400 MHz, MeOD–d$_4$): δ 6.94–6.92 (m, ArH), 6.57–6.55 (m, ArH), 4.47–4.41 (m), 4.33–4.31 (m), 4.25–4.20 (m), 4.00–3.90 (m), 3.63–3.56 (m), 3.19 (s), 3.13–3.07 (m), 2.92–2.87 (m), 2.77–2.69 (m), 1.47–1.39 (m), 1.26–1.24 (m, –CH, –2CH$_3$ peaks merged), 0.80–0.72 (m, –2CH$_3$); **^{13}C NMR (101 MHz, MeOD–d$_4$)**: δ 173.4, 173.0, 172.3, 171.8, 169.7, 166.3, 156.0, 155.9, 132.2, 132.0, 130.1, 129.9, 127.5, 117.2, 116.8, 114.9, 114.9, 55.0, 54.4, 52.4, 50.5, 49.5, 48.9, 48.8, 48.3, 42.4, 40.6, 40.2, 39.8, 36.5, 24.4, 21.9, 20.6, 17.3, 15.9, 11.7 (4x–CH$_3$, numbered 1-4 in red).

DEPT-135 (101 MHz, MeOD–d$_4$): 132.2, 132.0, 130.1, 129.9, 129.7, 114.9, 114.8, 56.7, 56.6, 55.6, 55.4, 54.9, 54.0, 52.4, 48.7, 48.7, 42.4, 38.0, 24.4, 17.3, 15.9, 11.7 (-CH$_2$ peaks are not resolved).

1H-1H COSY and HMQC analysis: Key correlations:

In the region of 1.26–1.24, three 3 diagonal spots present corresponding to Leu –CH (3), and two –CH$_3$ peaks (1 and 2) (circled in red color). The Leu –CH peaks are in correlation with methyl peaks evident from the presence off-diagonal spot. In 0.80–0.72 region, 2 methyl peaks of Leu are present (a and b) (circled in blue color). These correlations also can be seen in HMQC. 4 clear peaks for methyl groups present.

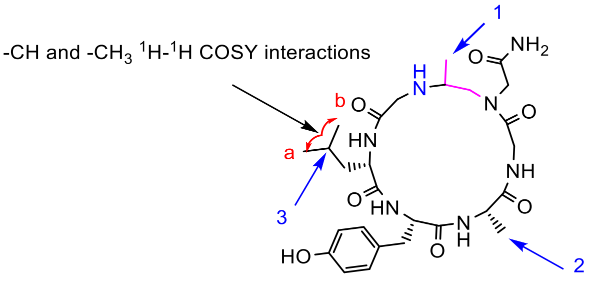

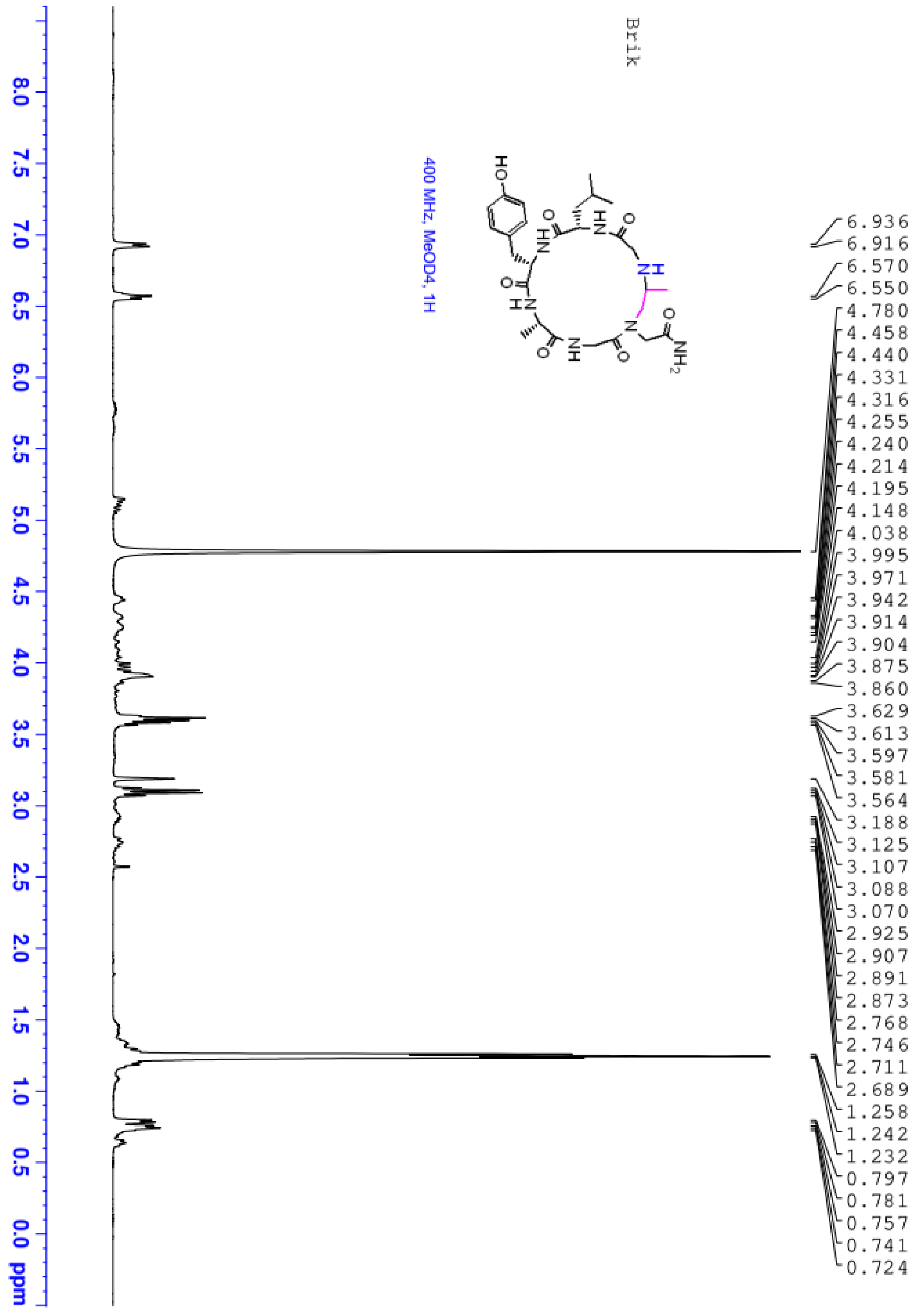
Brik
400 MHz, MeOD4, 1H
6.936
6.916
6.570
6.550
4.780
4.458
4.440
4.331
4.316
4.255
4.240
4.214
4.195
4.148
4.038
3.995
3.971
3.942
3.914
3.904
3.875
3.860
3.629
3.613
3.597
3.581
3.564
3.188
3.125
3.107
3.088
3.070
2.925
2.907
2.891
2.873
2.768
2.746
2.711
2.689
1.258
1.242
1.232
0.797
0.781
0.757
0.741
0.724
8.0
7.5
7.0
6.5
6.0
5.5
5.0
4.5
4.0
3.5
3.0
2.5
2.0
1.5
1.0
0.5
0.0 ppm

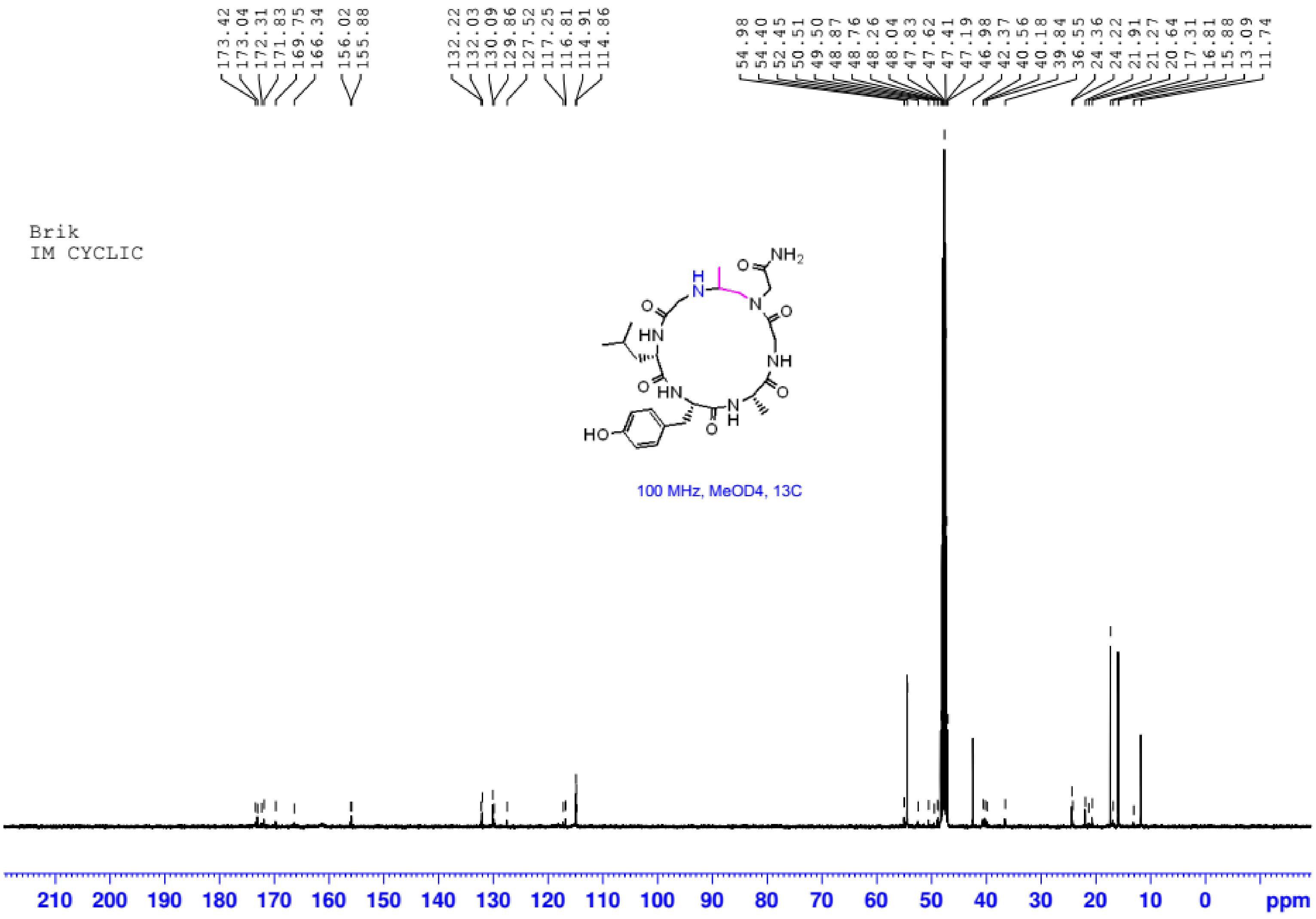
Brik
IM CYCLIC
173.42
173.04
172.31
171.83
169.75
166.34
156.02
155.88
132.22
132.03
130.09
129.86
127.52
117.25
116.81
114.91
114.86
54.98
54.40
52.45
50.51
49.50
48.87
48.76
48.26
48.04
47.83
47.62
47.41
47.19
46.98
42.37
40.56
40.18
39.84
36.55
24.36
24.22
21.91
21.27
20.64
17.31
16.81
15.88
13.09
11.74
100 MHz, MeOD4, 13C
210 200 190 180 170 160 150 140 130 120 110 100 90 80 70 60 50 40 30 20 10 0 ppm

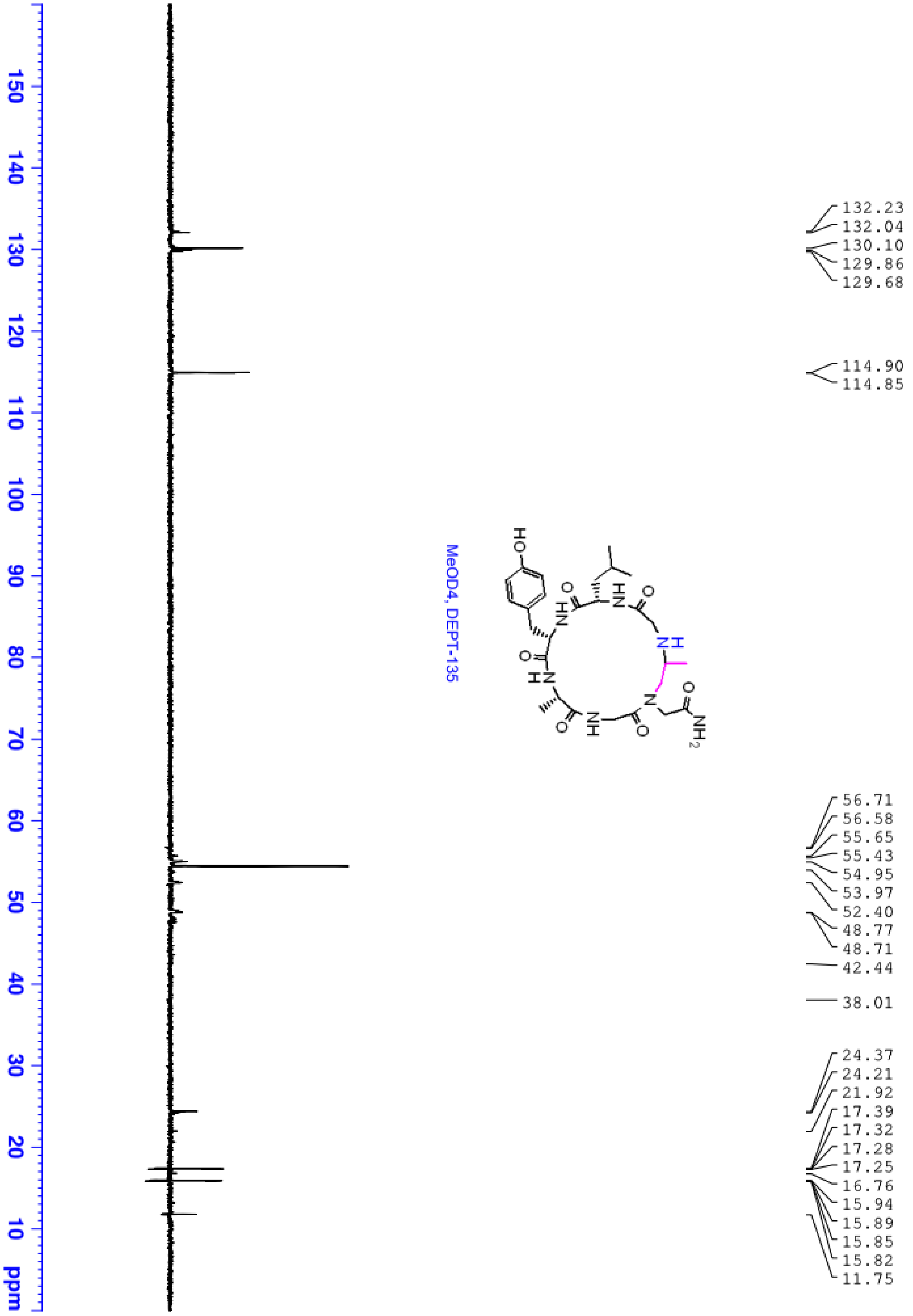

132.23
132.04
130.10
129.86
129.68
114.90
114.85
56.71
56.58
55.65
55.43
54.95
53.97
52.40
48.77
48.71
42.44
38.01
24.37
24.21
21.92
17.39
17.32
17.28
17.25
16.76
15.94
15.89
15.85
15.82
11.75
MeOD4, DEPT-135
150
140
130
120
110
100
90
80
70
60
50
40
30
20
10
ppm

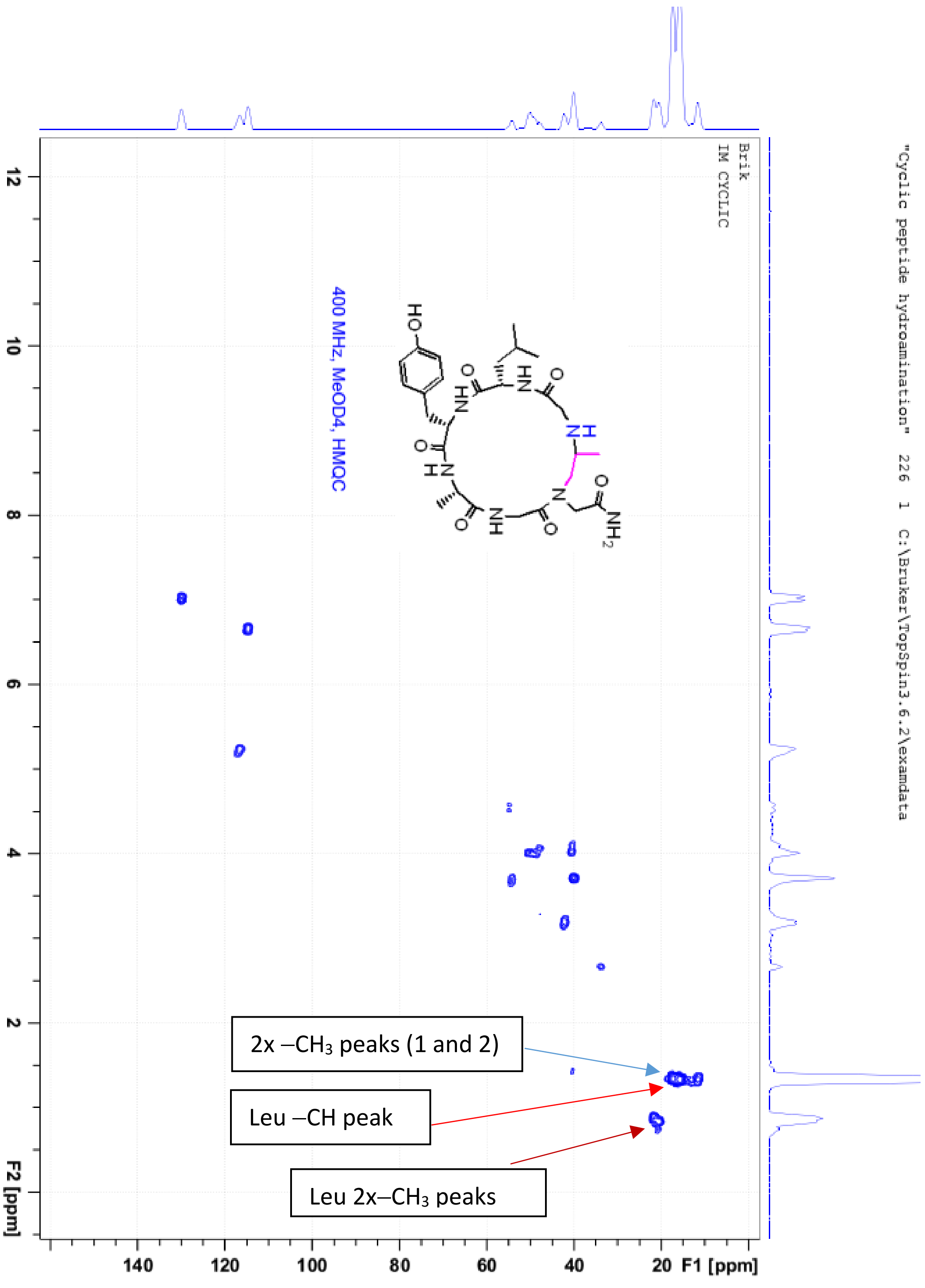
2x –CH3 peaks (1 and 2)
Leu –CH peak
Leu 2x–CH3 peaks
400 MHz, MeOD4, HMQC
Brik
IM CYCLIC
"Cyclic peptide hydroamination" 226 1 C:\Bruker\TopSpin3.6.2\examdata
F1 [ppm]
F2 [ppm]

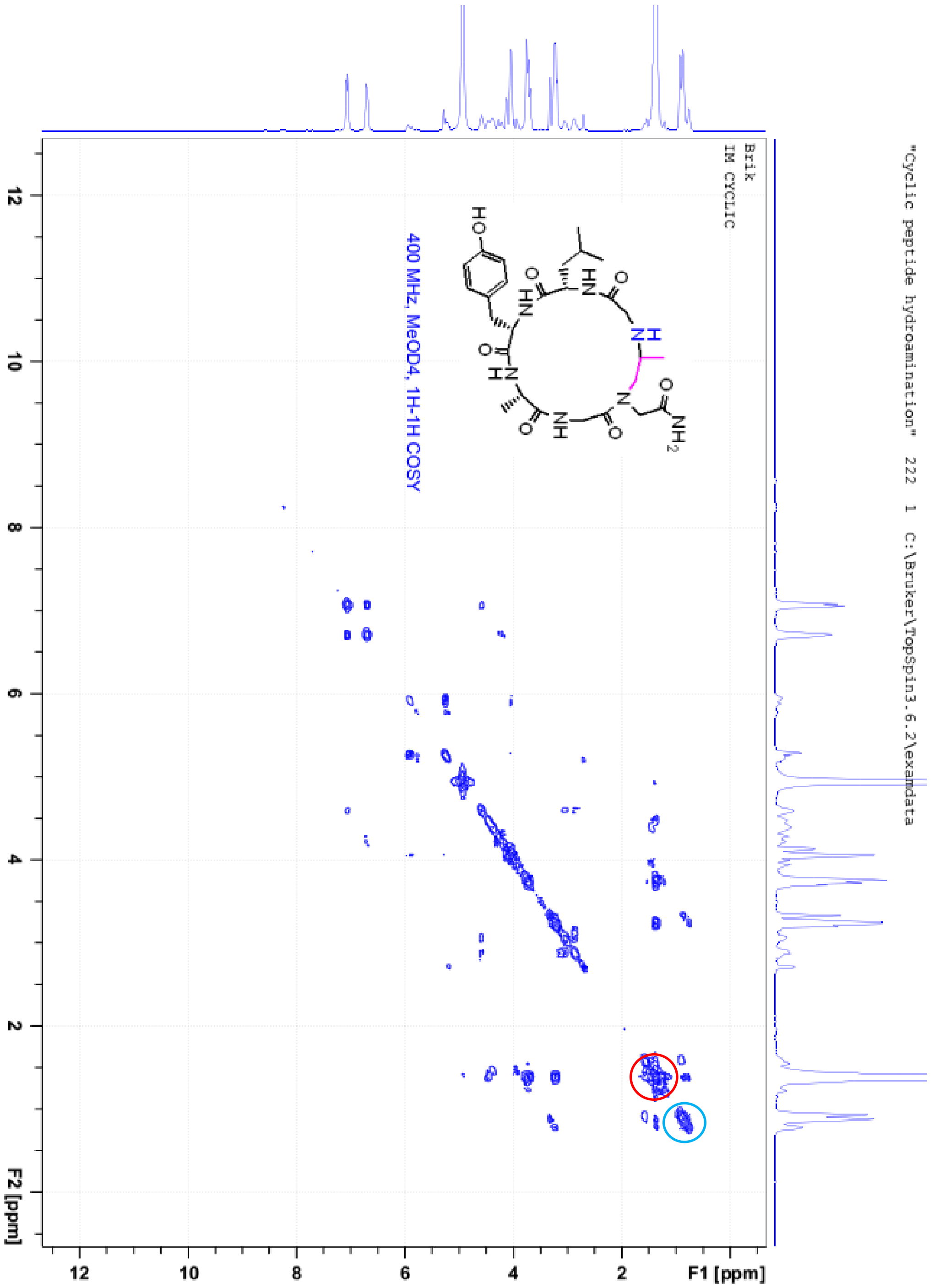
"Cyclic peptide hydroamination" 222 1 C:\Bruker\TopSpin3.6.2\examdata
Brik
IM CYCLIC
400 MHz, MeOD4, 1H-1H COSY
F1 [ppm]
F2 [ppm]

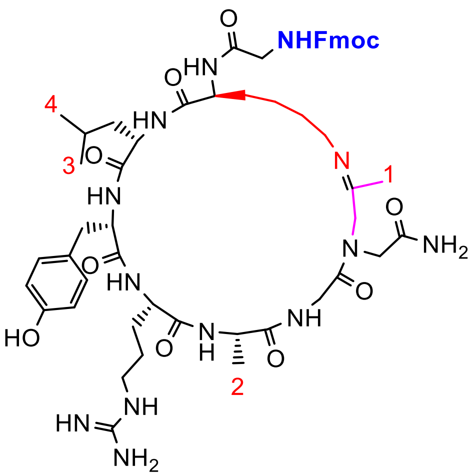

^{1}H NMR (600 MHz, MeOD–d$_4$): δ 8.25 (t, J = 6.0 Hz, 8.06–8.02 (m), 7.95 (t, J = 6.0 Hz), 7.86–7.83 (m), 7.73–7.67 (m), 7.53–7.49 (m), 7.28–7.26 (m), 7.21–7.16 (m), 6.90 (d, J = 12.0 Hz), 6.55 (d, J = 12.0 Hz), 4.22–3.90 (m), 3.75–3.66 (m), 3.06–3.03 (m), 2.80–2.74 (m), 1.82–1.78 (m, -CH_2), 1.68–1.61 (m, -CH_2), 1.57–1.51 (m, -CH_2), 1.44-1.38 (m, -CH_2), 1.36-1.35 (m, 2x-CH_3 peaks), 0.83-0.77 (m, 2x -CH_3 peaks).

DEPT-135 (151 MHz, MeOD–d$_4$): 131.5, 129.0, 128.3, 126.3, 121.1, 116.4, -68.6, -58.8, 56.9, 55.5, 55.4, 54.3, 54.2, 54.1, 50.6, -48.4, 48.3, -45.3, -44.5, -42.3, -42.1, -41.3, -40.7, 40.6, -37.8, 37.7, -30.5, 30.2, 28.1, -26.11, 26.06, -23.9, 23.5, -22.0.

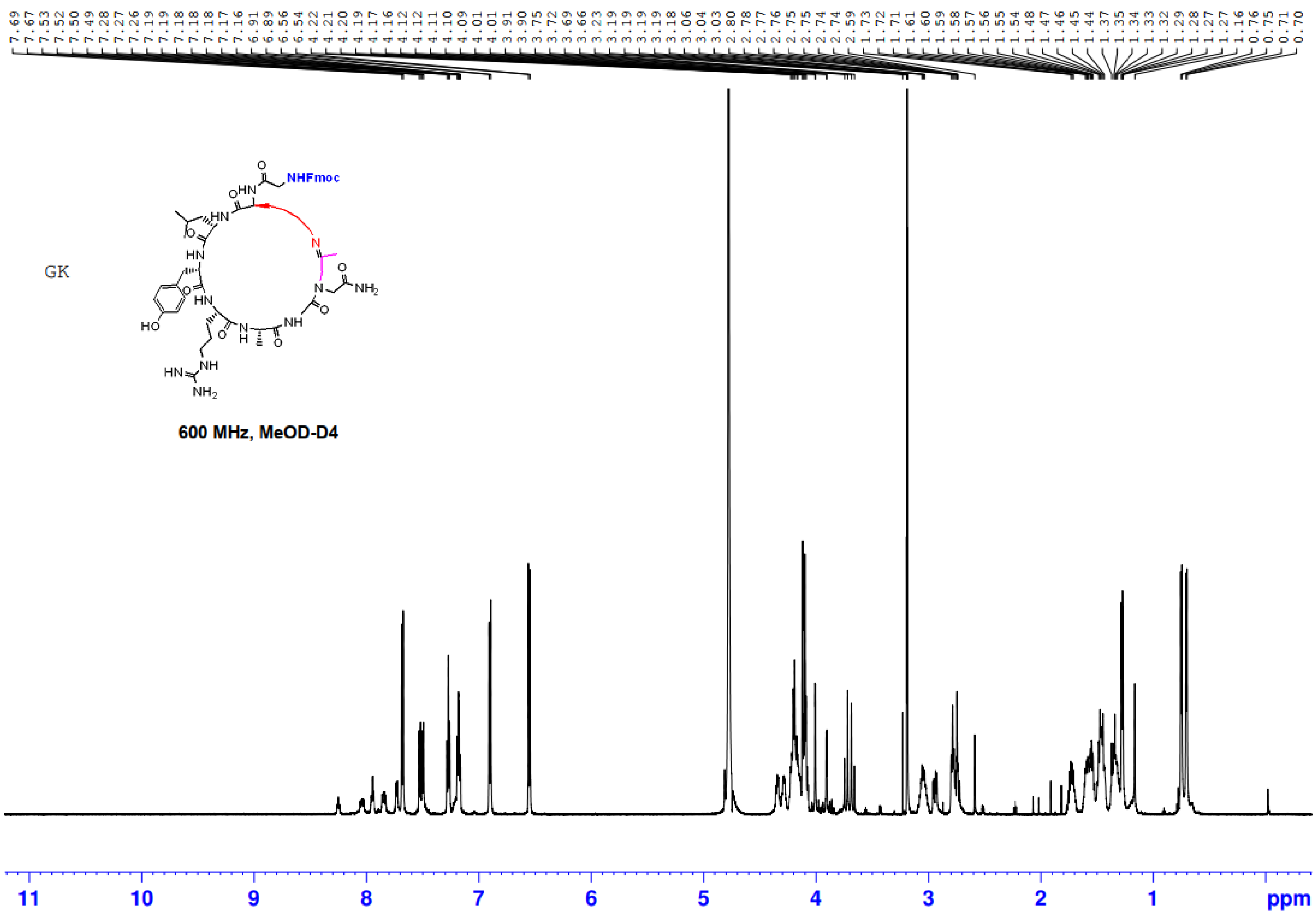
7.69
7.67
7.53
7.52
7.50
7.49
7.28
7.27
7.26
7.19
7.19
7.18
7.18
7.18
7.17
7.16
6.91
6.89
6.56
6.54
4.22
4.21
4.20
4.19
4.17
4.16
4.12
4.12
4.11
4.10
4.09
4.01
4.01
3.91
3.90
3.75
3.72
3.69
3.66
3.23
3.19
3.19
3.19
3.19
3.18
3.06
3.04
3.03
2.80
2.78
2.77
2.76
2.75
2.75
2.74
2.74
2.59
1.73
1.72
1.71
1.61
1.60
1.59
1.58
1.57
1.56
1.55
1.54
1.48
1.47
1.46
1.45
1.44
1.37
1.35
1.34
1.33
1.32
1.29
1.28
1.27
1.27
1.16
0.76
0.75
0.71
0.70
GK
NHFmoc
HN
O
N
NH2
NH
HO
HN
NH
NH2
600 MHz, MeOD-D4
11
10
9
8
7
6
5
4
3
2
1
ppm

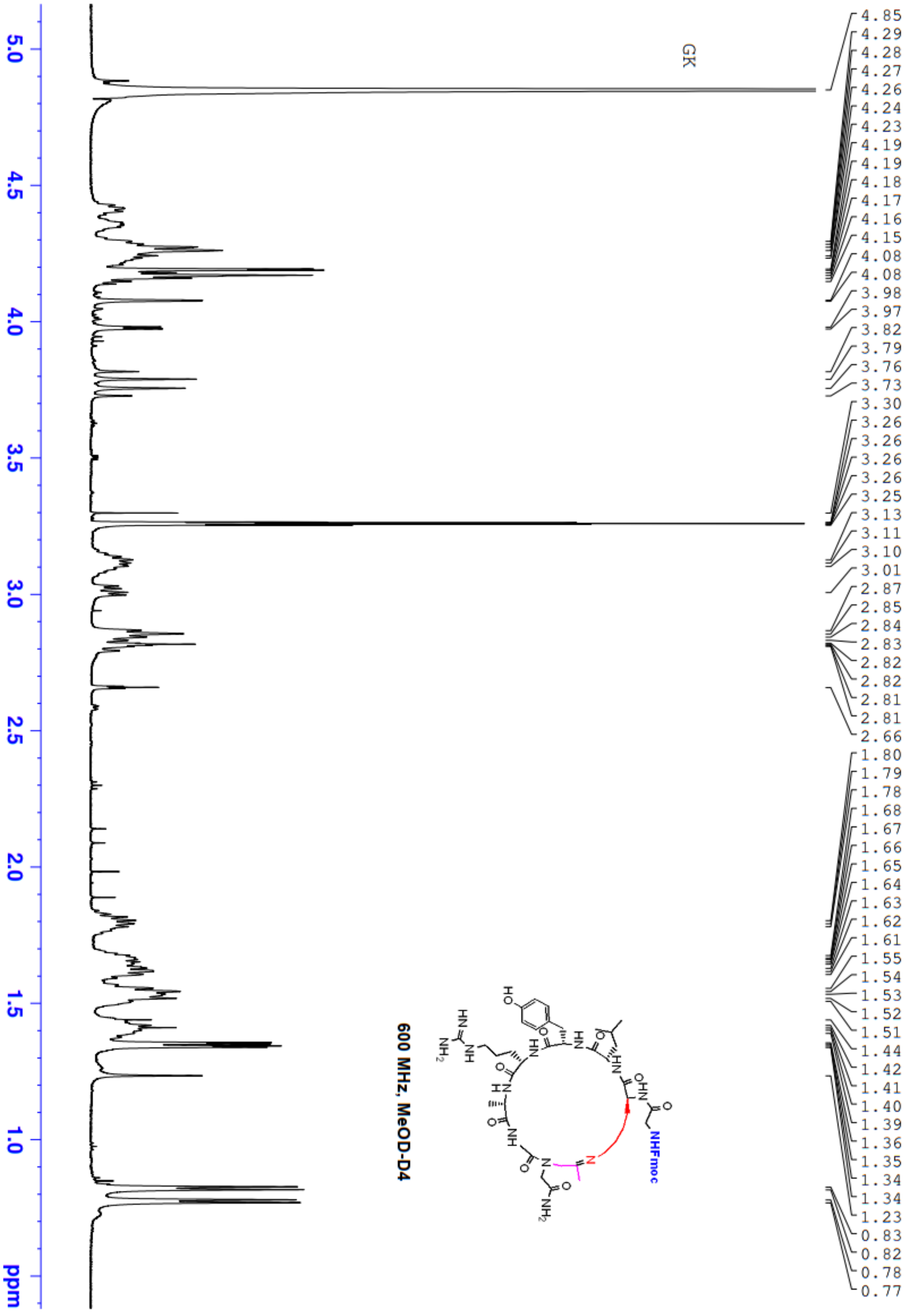
GK
600 MHz, MeOD-D4
NHFmoc
5.0
4.5
4.0
3.5
3.0
2.5
2.0
1.5
1.0
ppm
4.85 4.29 4.28 4.27 4.26 4.24 4.23 4.19 4.19 4.18 4.17 4.16 4.15 4.08 4.08 3.98 3.97 3.82 3.79 3.76 3.73 3.30 3.26 3.26 3.26 3.26 3.25 3.13 3.11 3.10 3.01 2.87 2.85 2.84 2.83 2.82 2.82 2.81 2.81 2.66 1.80 1.79 1.78 1.68 1.67 1.66 1.65 1.64 1.63 1.62 1.61 1.55 1.54 1.53 1.52 1.51 1.44 1.42 1.41 1.40 1.39 1.36 1.35 1.34 1.34 1.23 0.83 0.82 0.78 0.77

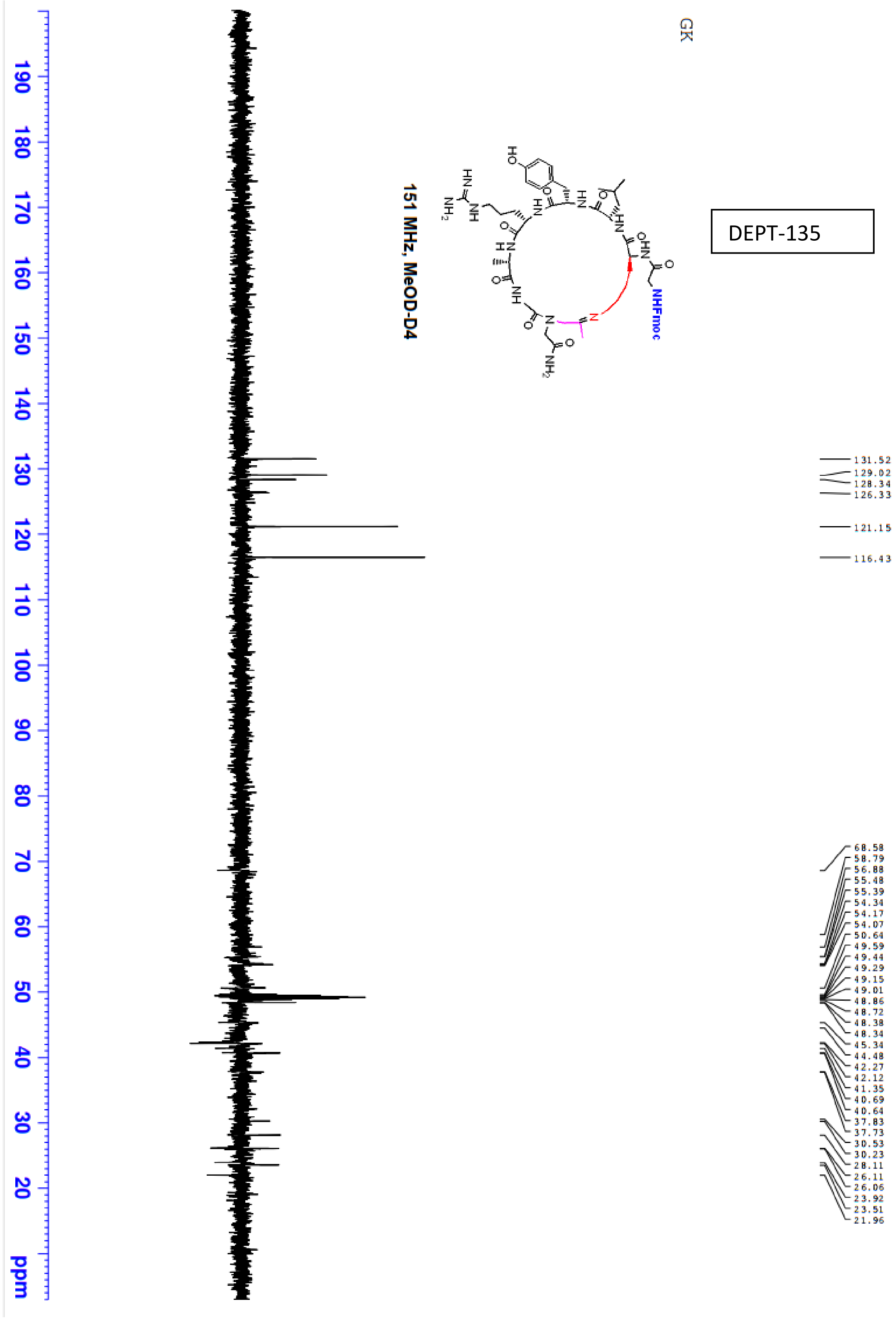
GK
DEPT-135
151 MHz, MeOD-D4
NHFmoc
131.52
129.02
128.34
126.33
121.15
116.43
68.58
58.79
56.88
55.48
55.39
54.34
54.17
54.07
50.64
49.59
49.44
49.29
49.15
49.01
48.86
48.72
48.38
48.34
45.34
44.48
42.27
42.12
41.35
40.69
40.64
37.83
37.73
30.53
30.23
28.11
26.11
26.06
23.92
23.51
21.96
ppm

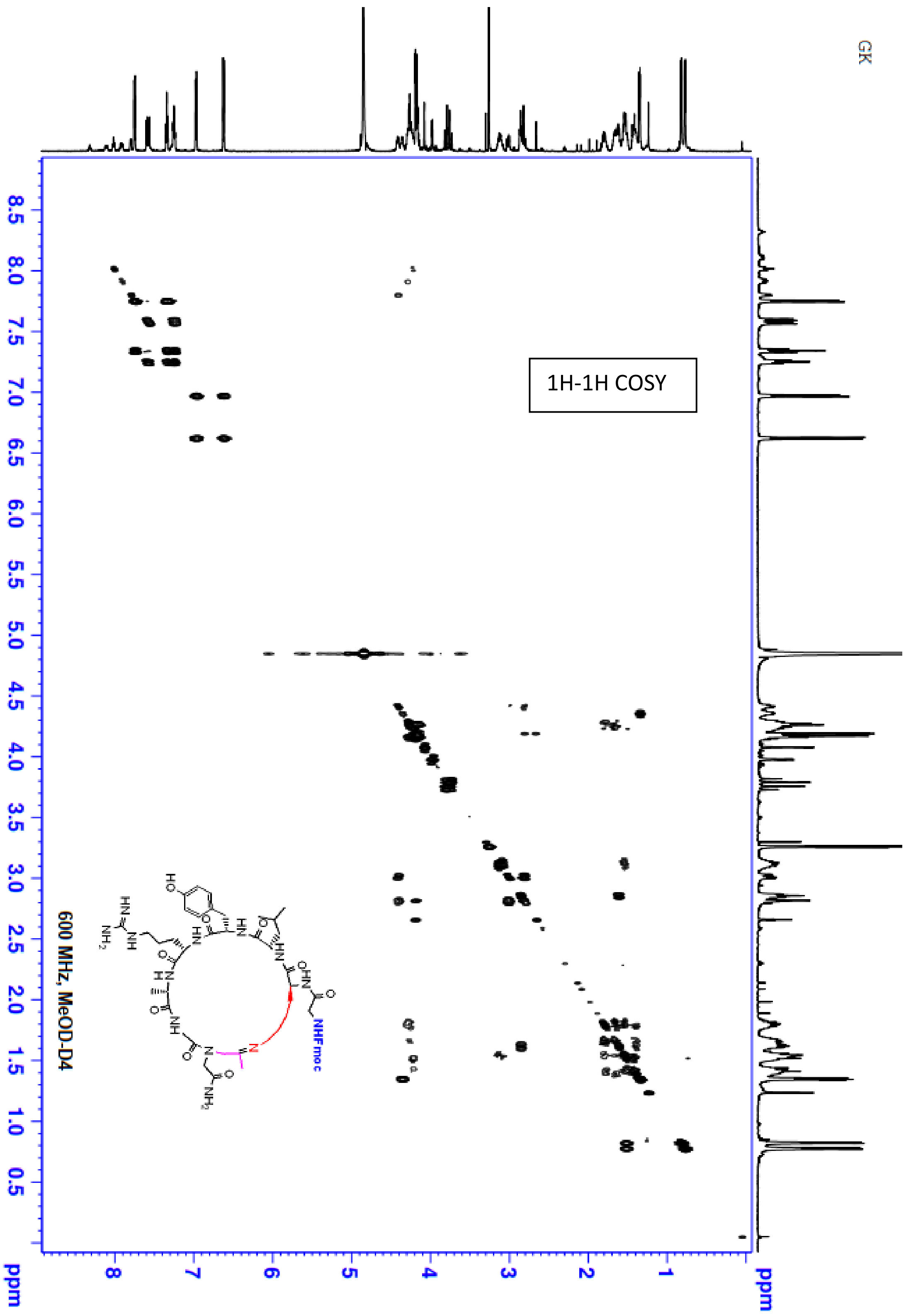
1H-1H COSY
600 MHz, MeOD-D4
NHFmoc
ppm
ppm
GK
